# Synthesis and Evaluation of Novel DNA Minor Groove Binders as Antiamoebic Agents

**DOI:** 10.3390/antibiotics11070935

**Published:** 2022-07-13

**Authors:** Hasan Y. Alniss, Naveed A. Khan, Anania Boghossian, Noor Akbar, Hadeel M. Al-Jubeh, Yousef A. Msallam, Balsam Q. Saeed, Ruqaiyyah Siddiqui

**Affiliations:** 1 College of Pharmacy, University of Sharjah, Sharjah P.O. Box 27272, United Arab Emirates; u18103456@sharjah.ac.ae; 2 Sharjah Institute for Medical Research, University of Sharjah, Sharjah P.O. Box 27272, United Arab Emirates; u00033543@sharjah.ac.ae; 3 Department of Clinical Sciences, College of Medicine, University of Sharjah, Sharjah P.O. Box 27272, United Arab Emirates; nakbar@sharjah.ac.ae (N.A.); bsaeed@sharjah.ac.ae (B.Q.S.); 4 College of Arts and Sciences, American University of Sharjah, University City, Sharjah P.O. Box. 26666, United Arab Emirates; g00073387@alumni.aus.edu (A.B.); rsiddiqui@aus.edu (R.S.)

**Keywords:** *Acanthamoeba castellanii*, trophozoites, cysts, sight-threatening, DNA minor groove binders, distamycin analogues, organic synthesis

## Abstract

The free-living amoeba *Acanthamoeba castellanii* is responsible for the central nervous infection granulomatous amoebic encephalitis and sight-threatening infection *Acanthamoeba keratitis*. Moreover, no effective treatment is currently present, and a combination drug therapy is used. In this study, twelve DNA minor groove binders (MGBs) were synthesized and tested for their antiamoebic activity via amoebicidal, encystation, excystation, and cytopathogenicity assays. It was found that the compounds MGB3, MGB6, MGB22, MGB24, and MGB16 significantly reduce amoeba viability to 76.20%, 59.45%, 66.5%, 39.32%, and 43.21%, respectively, in amoebicidal assays. Moreover, the compounds MGB6, MGB20, MGB22, MGB28, MGB30, MGB32, and MGB16 significantly inhibit *Acanthamoeba* cysts, leading to the development of only 46.3%, 39%, 30.3%, 29.6%, 27.8%, 41.5%, and 45.6% cysts. Additionally, the compounds MGB3, MGB4, MGB6, MGB22, MGB24, MGB28, MGB32, and MGB16 significantly reduce the re-emergence of cysts to trophozoites, with viable trophozoites being only 64.3%, 47.3%, 41.4%, 52.9%, 55.4%, 40.6%, 62.1%, and 51.7%. Moreover, the compounds MGB3, MGB4, and MGB6 exhibited the greatest reduction in amoeba-mediated host-cell death, with cell death reduced to 41.5%, 49.4%, and 49.5%. With the following determined, future in vivo studies can be carried out to understand the effect of the compounds on animal models such as mice.

## 1. Introduction

Commonly found in the environment all around the world is the free-living, opportunistic protozoa *Acanthamoeba* [1,2,3]. *Acanthamoeba castellanii* is responsible for the fatal central nervous infection granulomatous amoebic encephalitis (GAE) and the sight-threatening infection *Acanthamoeba* keratitis (AK) [1,4]. This protozoon exists in two stages: an active, infective trophozoite stage; and a dormant, inactive cyst stage [4]. Making matters worse is the rise of global temperatures, global warming, and water supply shortages, as the amoeba is likely to be found in lakes, swimming pools, and infected water tanks [2,4]. However, the amoeba may also be found in air-conditioning vents, contact-lens solution, ventilation vents, and heating vents [2]. The amoeba is capable of withstanding less hospitable conditions by transforming into cysts, as they are more resistant to harsh environmental conditions and treatments [2,4]. The high resistance of cysts towards drugs is possibly due to the high amount of cellulose present in the outer wall of the cysts [2]. Towards the end of a treatment, the cysts may then revert to trophozoites and re-infect the individual [4].

Currently, a combination drug therapy is used to fight against AK; a combination treatment of propamidine isethionate and neomycin is used as a topical treatment option [4]. Additionally, biguanide and diamidine antimicrobial, antiseptic, antiparasitic, antibiotic, and antifungal agents are used for clinically resistant cases [1]. However, more efficient, biologically safe treatments are needed. DNA minor groove binders (MGBs) are small molecules that are capable of disrupting DNA structure and function by targeting the minor groove of duplex DNA [5,6,7]. The majority of these compounds was synthesized as analogues of the natural DNA binding agent distamycin, which possesses a crescent shape that matches the curvature of DNA in the minor groove. The ability of these compounds to bind reversibly with the minor groove without causing permeant DNA damage, as encountered with the currently available toxic chemotherapeutics, offered them significant biological effects against multiple targets with good safety profile [8]; for instance, these compounds have shown therapeutic potential in the treatment of cancer [9,10], viral [11,12], bacterial [13], fungal [14], and parasitic diseases [15,16,17]. Hence, in this study, novel DNA minor groove binders were synthesized and tested for their antiamoebic properties via amoebicidal assays. The compounds’ ability to induce encystation and excystation were studied through the conduction of encystation and excystation assays. Additionally, the safety of the compounds against human cells was determined through cytotoxicity assays. Moreover, the amoeba-mediated host-cell death was studied through cytopathogenicity assays.

## 2. Materials and Methods

### 2.1. General

Solvents and chemical reagents were purchased from Sigma-Aldrich (Steinheim, Germany) and Santa Cruz Biotechnology Inc. (Santa Cruz, CA, USA) and were used without further purification. Both ^1^H and ^13^C NMR experiments were measured on a Bruker 500 MHz instrument with chemical shifts given in parts per million, ppm (δ-values) relative to the internal tetramethylsilane standard. NMR data were processed by using the academic edition of the ACD NMR software. The NMR data were presented as follows: s (singlet), d (doublet), t (triplet), q (quartet), m (multiplet), dd (double doublet), or bs (broad singlet); coupling constants, J, are reported in hertz (Hz). The structures of intermediates and the final MGB products were confirmed by NMR spectroscopy and mass spectrometry (Supplementary Materials). Electrospray ionization–mass spectrometry (ESI–MS) experiments were carried out on a triple-quadrupole tandem mass spectrometer (MicromassW Quattro microTM Waters Corp., Milford, MA, USA) coupled with an electrospray ionization (ESI) interface operated in positive ionization mode. Thin-layer chromatography (TLC) experiments were performed to monitor the reactions, using silica gel plates (0.25 mm, E. Merck, 60 F254) and a UV lamp for visualization. HPLC purification was carried out for all final products, using Agilent 1260 infinity liquid chromatography (Agilent Technologies, Palo Alto, CA, USA), a Zorbax-SB-C18 column (5 μm; 25 cm × 9.4 mm i.d) with the general gradient elution described below, and a detection wavelength of 260 nm.

HPLC gradient elution.

**Time (min)**

**Flow Rate (mL/min)**

**% Water (0.1% TFA)**

**% Acetonitrile (0.1% TFA)**
05802025550502650100305010031580203558020


### 2.2. Synthesis of 1-methyl-N-(3-morpholinopropyl)-4-nitro-1H-pyrrole-2-carboxamide (***2a***)

A solution of 4-(3-aminopropyl) morpholine (2.93 g, 20.40 mmol) in dry DCM (10 mL) was cooled in an ice-water bath and stirred while a solution of 4-nitro-1-methyl-2-trichloroacetylpyrrole **1** (5.0 g, 0.01852 mol) in dry DCM (15 mL) was added slowly, over a period of five minutes. Then the mixture was stirred at room temperature for four hours, during which a precipitate formed. The solvent was evaporated under reduced pressure. Diethyl ether (30 mL) was added, and the product was collected by filtration, washed with ether, and dried under reduced pressure to produce the product as white solid crystals. Yield (75%); ^1^H NMR (CHLOROFORM-d) δ 1.77 (2H, m, CH_2_), 2.37 (6H, s, NCH_3_), 2.56 (2H, t, NCH_2_), 3.50 (2H, q, CONH—CH_2_), 4.01 (3H, s, NCH_3_), 6.98 (1H, d, Ar—H), 7.53 (1H, d, Ar—H), 8.67 (1H, s, CONH); ^13^C NMR (CHLOROFORM-d): δ 24.60, 37.88, 40.19, 45.25, 59.37, 106.51, 126.28, 126.92, 134.91, 160.26; LC–MS (ESI): *m*/*z* calcd for C_11_H_18_N_4_O_3_, 254.14, found 255.21 [M + H]^+^.

### 2.3. Synthesis of N-(3-(dimethylamino) propyl)-1-methyl-4-nitro-1H-pyrrole-2-carboxamide (***2b***)

A solution of 4-nitro-1-methyl-2-trichloroacetylpyrrole (**1**) (5.0 g, 18.52 mmol) in dry DCM (15 mL) was cooled in an ice-water bath and stirred while a solution of dimethylaminopropyl amine (2.705 g, 26.48 mmol) in dry DCM (5 mL) was added slowly, during a period of 5 min; the mixture was then stirred at room temperature overnight. The solvent was evaporated under reduced pressure, and the crude product was re-crystallized by using water:ethanol (80:20%), filtered, and washed with hexane to obtain the product in the form of white solid crystals. Yield (70%); ^1^H NMR (CHLOROFORM-d) δ 1.79 (2H, m, CH_2_), 2.53 (4H, s, NCH_2_), 2.57 (2H, t, NCH_2_), 3.50 (2H, q, CONH--CH_2_), 3.76 (4H, t, OCH_2_), 4.00 (3H, s, NCH_3_), 7.10 (1H, d, Ar—H), 7.54 (1H, d, Ar—H), 7.94 (1H, s, CONH); ^13^C NMR (CHLOROFORM-d): δ 23.55, 37.51, 39.77, 53.61, 58.39, 66.58, 106.45, 126.06, 126.49, 134.62, 160.02; LC−MS (ESI): *m*/*z* calcd for C_13_H_20_N_4_O_4_, 296.15, found 297.14 [M + H]^+^.

### 2.4. Synthesis of 1-methyl-N-[1-methyl-5-(carbonyl)-1H-pyrrol-3-yl]-4-nitro-1H-pyrrole-2-carboxamide (***8***)

First, 1-methyl-*N*-[2-(4-morpholinyl)ethyl]-4-nitro-1*H*-pyrrole-2-carboxamide (3.0 g, 10.13 mmol) was dissolved in THF (12 mL) and cooled to 0 °C. Then 10%-Pd/C (0.5 g) was added in small portions, and the flask was repeatedly evacuated and refilled with hydrogen gas; the suspension was then stirred under hydrogen for 6 h. The suspension was then filtered, and the THF was removed under reduced pressure to produce an oily amine product which was then diluted with 2 mL THF and added dropwise to a solution of 1-methyl-4-nitro-1H-pyrrole-2-carboxylic acid **4a** (1.2918 g, 7.545 mmol), triethylamine (5.585 mL, 45.31 mmol), and HBTU (0.331 g, 8.73 mmol) in THF (10 mL) and left stirring overnight, at room temperature, under nitrogen gas. The solvent was removed under reduced pressure, and the crude product obtained was purified on basified silica, using a 5:94:1 solution of MeOH/EA/TEA to obtain the product as white solid. Yield (68%); ^1^H NMR (ACETIC ACID-d_4_) δ 2.01 (2H, t, NCH_2_), 3.04 (4H, s, NCH_2_), 3.51 (2H, q, CONH-CH_2_), 3.90 (4H, s, OCH_2_) 3.93 (3H, s, NCH_3_), 4.07 (3H, s, NCH_3_), 6.98 (1H, d, Ar—H), 7.28 (1H, d, Ar—H), 7.34 (1H, d, Ar—H), 7.94 (1H, d, Ar—H), 9.52 (1H, s, CONH); ^13^C NMR (ACETIC ACID-d_4_): δ15.16, 17.54, 25.98, 26.77, 27.14, 33.12, 45.90, 94.87, 97.26, 112.50, 114.20, 117.02, 117.18, 137.53, 149.71; LC−MS (ESI): *m*/*z* calcd for C_19_H_26_N_6_O_5_, 418.2, found 419.12 [M + H]^+^.

### 2.5. Synthesis of N-[3-(dimethylamino) propyl]-1-methyl-4-{[(1-methyl-4-nitro-1H-pyrrol-2-yl)carbonyl]amino}-1H-pyrrole-2-carboxamide (***7***)

*N*-[3-(Dimethylamino)propyl]-1-methyl-4-nitro-1*H*-pyrrole-2-carboxamide (0.40 g, 1.5739 mmol) was dissolved in methanol (10 mL) and cooled to 0 °C. Then 10%-Pd/C (0.1 g) was added in small portions, and the flask was repeatedly evacuated and refilled with hydrogen gas; the suspension was then stirred under hydrogen for 4 h The suspension was then filtered, and the solvent was removed under reduced pressure to produce an oily amine product which was diluted with 2 mL THF and added dropwise to 1-methyl-4-nitro-1H-pyrrole-2-carboxylic acid **4a** (0.2406 g, 1.411 mmol), triethylamine (1.179 g, 11.65 mmol), and HBTU (0.716 g, 1.887 mmol) in THF (10 mL) and left stirring overnight, at room temperature, under nitrogen gas. The solvent was removed under reduced pressure, and sodium bicarbonate solution (10 mL) was added to the crude product, which was then left stirring for 10 min. The desired product was then precipitated as yellow solid and collected by filtration and dried under vacuum. Yield (55%); ^1^H NMR (CHOLOROFORM-d) δ 1.84 (2H, m, CH_2_), 2.44 (6H, s, NCH_3_), 2.65 (2H, t, NCH_2_), 3.48 (2H, q, CONH--CH_2_), 3.93 (3H, s, NCH_3_), 4.04 (3H, s, NCH_3_), 6.58 (1H, d, Ar—H), 7.25 (1H, d, Ar—H), 7.32 (1H, d, Ar—H), 7.60 (1H, d, Ar—H), 7.76 (1H, s, CONH), 8.11 (1H, s, CONH); ^13^C NMR (CHLOROFORM-d): δ 14.27, 22.84, 25.58, 29.51, 29.85, 32.08, 36.88, 38.10, 45.19, 103.1, 107.15, 118.89, 120.64, 124.22, 127.03, 161.75; LC−MS (ESI): *m*/*z* calcd for C_17_H_24_N_6_O_4_, 376.09, found 377.02 [M + H]^+^.

### 2.6. Synthesis of methyl 1-methyl-4-nitro-1H-pyrrole-2-carboxylate (***4b***)

First, 4-nitro-1-methyl-2-trichloroacetylpyrrole **1** (2.0 g, 7.4 mmol) was dissolved in methanol (14 mL). Sodium methoxide (0.48 g, 8.8 mmol) was dissolved in methanol (10 mL) and then added to **1** while stirring, and the reaction was refluxed (70 °C) for 1 h. The solvent was evaporated under reduced pressure; then cold distilled water was added, and the product was precipitated as white powder, which was then filtered, washed with a 1:1 water:methanol solution, and dried under vacuum. Yield (45%); ^1^H NMR (CHLOROFORM-d) δ 3.87 (3H, s, OCH_3_), 4.00 (3H, s, NCH_3_), 7.42 (1H, d, Ar—H), 7.61 (1H, d, Ar—H); ^13^C NMR (CHLOROFORM-d): δ 37.97, 51.86, 112.78, 122.84, 127.53, 160.62; LC−MS (ESI): *m*/*z* calcd for C7H8N2O4, 184.85, found 184.89 [M + H]^+^.

### 2.7. Synthesis of methyl 1-methyl-4-(1-methyl-1H-imidazole-5-amido)-1H-pyrrole-2-carboxylate (***6a***)

Methyl 1-methyl-4-nitro-1H-pyrrole-2-carboxylate **4b** (0.40 g, 2.1733 mmol) was dissolved in THF (4 mL) and methanol (4 mL) and cooled to 0 °C. Then 10%-Pd/C (150 mg) was added portion-wise, while stirring. The flask was repeatedly evacuated and refilled with hydrogen gas, and the reaction was left stirring for 4 h. The Palladium on carbon was filtered through celite, and the solvent was then removed under reduced pressure to yield oily amine product **5a**, which was then diluted with 2 mL THF and added dropwise to a solution of 1-Methyl-Imdazole-5-Carboxylic Acid **5c** (0.274 g, 2.1733 mmol), triethylamine (1.305 g, 12.9 mmol), and HBTU (0.989 g, 2.608 mmol) in dry DMF (5 mL) and left stirring overnight, at room temperature, under nitrogen gas. The solvent was removed under reduced pressure, and a solution of sodium carbonate (15 mL) was added and left stirring for 10 min. The desired product was precipitated, filtered, and then washed with distilled water and dried under vacuum. Yield (60%); ^1^H NMR (ACETIC ACID-d_4_) δ 3.75 (3H, s, OCH_3_), 3.90 (3H, s, NCH_3_), 3.94 (3H, s, NCH_3_), 6.88 (1H, d, Ar—H), 7.45 (1H, d, Ar—H), 7.59 (1H, d, Ar—H), 7.65 (1H, d, Ar—H); ^13^C NMR (ACETIC ACID-d_4_): δ 24.43, 27.07, 41.53, 99.30, 99.36, 110.66, 111.82, 113.89, 123.00, 133.27, 148.66, 152.25; LC−MS (ESI): *m*/*z* calcd for C12H14N4O3, 262.11, found 263.11 [M + H]^+^.

### 2.8. Synthesis of methyl 1-methyl-4-(1-methyl-1H-imidazole-5-amido)-1H-pyrrole-2-carboxylic Acid (***9***)

Methyl 1-methyl-4-(1-methyl-1H-imidazole-5-amido)-1H-pyrrole-2-carboxylate **6a** (0.280 g, 1.0697 mmol) was dissolved in THF (3 mL) and methanol (1 mL). Lithium hydroxide (0.1537 g, 6.4186 mmol) was dissolved in distilled water (2 mL) and added to **6a** solution; the reaction was then left stirring for 4 h. The organic solvent was evaporated under reduced pressure, and the aqueous layer was acidified by using 1N HCl solution. The desired product was precipitated, filtered, and washed with distilled water; it was then dried under vacuum to obtain the desired product as an off-white solid. Yield (90%); ^1^H NMR (DMSO-d_6_) δ 3.82 (3H, s, NCH_3_), 3.98 (3H, s, NCH_3_), 6.98 (1H, d, Ar—H), 7.03 (1H, d, Ar—H), 7.38 (1H, d, Ar—H), 7.47 (1H, d, Ar—H), 10.46 (1H, s, COOH); ^13^C NMR (DMSO-d_6_): δ 35.10, 36.20, 99.53, 108.85, 119.81, 120.40, 121.91, 126.41, 127.02, 138.64, 156.09, 161.99; LC−MS (ESI): *m*/*z* calcd for C_11_H_12_N_4_O_3_, 248.09, found 249.04 [M + H]^+^.

### 2.9. Synthesis of methyl 1-methyl-4-(1,3-thiazole-4-amido)-1H-pyrrole-2-carboxylate (***6b***)

Methyl 1-methyl-4-nitro-1H-pyrrole-2-carboxylate (**4b**) (0.50 g, 2.7166 mmol) was dissolved in THF (4 mL) and LCMS methanol (4 mL) and cooled to 0 °C. Then 10%-Pd/C (200 mg) was added portion-wise, while stirring. The flask was repeatedly evacuated and refilled with H_2_ gas, and the reaction was left stirring for 4 h. The Palladium on carbon was filtered through celite, and the solvent was removed under reduced pressure to yield an oily amine product which was then diluted with 2 mL DCM and added dropwise to a solution of 4-Thizole-Carboxylic Acid **5b** (0.280 g, 2.173 mmol,), triethylamine (1.042 g, 10.299 mmol), and HBTU (1.236 g, 3.26 mmol) in dry DCM (5 mL) and left stirring overnight, at room temperature, under nitrogen gas. The solvent was removed under reduced pressure, and a solution of sodium carbonate (15 mL) was added and left stirring for 10 min. The desired product precipitated, was filtered, and then was washed with distilled water and dried under vacuum to obtain the product as a white powder. Yield (65%); ^1^H NMR (CHLOROFORM-d) δ 3.83 (3H, s, OCH_3_), 3.94 (3H, s, NCH_3_), 6.85 (1H, d, Ar—H), 7.54 (1H, d, Ar—H), 8.24 (1H, d, Ar—H), 8.81 (1H, d, Ar—H), 9.10 (1H, s, CONH); ^13^C NMR (CHLOROFORM-d): δ 36.86, 51.14, 108.20, 120.03, 121.02, 121.22, 123.35, 150.80, 152.79, 157.91, 161.48; LC−MS (ESI): *m*/*z* calcd for C_11_H_11_N_3_O_3_S, 265.15, found 266.18 [M + H]^+^.

### 2.10. Synthesis of methyl 1-methyl-4-(1,3-thiazole-4-amido)-1H-pyrrole-2-carboxylic Acid (***10***)

Methyl 1-methyl-4-(1,3-thiazole-4-amido)-1H-pyrrole-2-carboxylate **6b** (0.223 g, 9.303 mmol) was dissolved in Tetrahydrofuran (3 mL) and HPLC-grade methanol (1 mL). Lithium Hydroxide (0.223 g, 9.30 mmol) was dissolved in distilled water (2 mL) and added to **6b** solution, and the reaction was left stirring for 4 h. The organic solvents were evaporated under reduced pressure, and the aqueous layer was acidified by using 1N HCl solution. The desired product was precipitated, filtered, and washed with distilled water; it was then dried under vacuum to obtain the desired product as an off-white powder. Yield (85%); ^1^H NMR (DMSO-d_6_) δ 3.83 (3H, s, NCH_3_), 7.02 (1H, d, Ar—H), 7.50 (1H, d, Ar—H), 8.39 (1H, d, Ar—H), 9.23 (1H, d, Ar—H), 10.49 (1H, s, COOH), 12.21 (1H, s, CONH); ^13^C NMR (DMSO-d_6_): δ 25.13, 36.23, 109.04, 120.61, 121.99, 124.58, 150.71, 154.93, 157.76, 161.97; LC−MS (ESI): *m*/*z* calcd for C_10_H_9_N_3_O_3_S, 251.04, found 251.89 [M + H]^+^.

### 2.11. Synthesis of methyl 1-methyl-4-(1-methyl-1H-imidazole-5-amido)-1H-pyrrole-2-carboxylic Acid (***4a***)

Methyl 1-methyl-4-nitro-1H-pyrrole-2-carboxylate **4b** (5.0 g, 27.16 mmol) was dissolved in THF (10 mL). Sodium hydroxide (4.5 g, 112.5 mmol) was dissolved in distilled water (15 mL) and added to **4b** solution, and the reaction was left stirring overnight. The organic solvent was evaporated under reduced pressure, and the aqueous layer was acidified by using 1N HCl solution. The desired product was precipitated, filtered, and washed with distilled water; it was then dried under vacuum to obtain the desired product as a white solid. Yield (81%); ^1^H NMR (DMSO-d_6_) δ 3.91 (3H, s, NCH_3_), 7.26 (1H, d, Ar—H), 8.23 (1H, d, Ar—H); ^13^C NMR (DMSO-d_6_): δ 37.50, 111.41, 123.81, 129.21, 134.02, 160.99; LC−MS (ESI): *m*/*z* calcd for C_6_H_6_N_2_O_4_, 170.03, found 169.01 [M − H]^−^.

### 2.12. Synthesis of methyl 2-(1-methyl-4-nitro-1H-pyrrole-2-carboxamido)-5-isopropylthiazole-4-carboxylate (***3***)

Methhyl-2-amino-5-isopropyl thiazole-4-carboxylate **2** (0.400 g, 1.9994 mmol) was dissolved in anhydrous DCM (3–4 mL) and TEA (0.607 g, 5.998 mmol). This solution was added dropwise to a solution of *N*-Methyl-4-Nitro-2-trichloroacetylpyrrole **1** (0.404 g, 1.499 mmol) dissolved in anhydrous DCM (3 mL), and the reaction was left stirring under nitrogen gas for 1 h. The solvent was removed under reduced pressure, a solution of sodium carbonate (10 mL) was added, and the product was then filtered and washed with 40:60 MeOH:water and dried under reduced pressure. Yield (70%); ^1^H NMR (CHOLOROFORM-d) δ 1.38 (6H, d, CH_3_), 3.89 (3H, s, OCH_3_), 4.09 (3H, d, NCH_3_), 4.14 (1H, m, CH), 7.30 (1H, d, AR—H), 7.68 (1H, d, Ar—H); ^13^C NMR (CHLOROFORM-d): δ 24.85, 27.79, 38.52, 52.41, 100.13, 109.95, 123.69, 128.58, 135.69, 153.30, 154.46, 157.85, 161.83; LC−MS (ESI): *m*/*z* calcd for C_14_H_16_N_4_O_4_S, 352.08, found 353.02 [M + H]^+^.

### 2.13. Synthesis of 2-(1-methyl-4-nitro-1H-pyrrole-2-carboxamido)-5-isopropylthiazole-4-carboxylic Acid (***4***)

Methyl 2-(1-methyl-4-nitro-1H-pyrrole-2-carboxamido)-5-isopropylthiazole-4-carboxylate **3** (3.8 g, 10.793 mmol) was dissolved in THF (10 mL). Lithium hydroxide (1.551 g, 64.758 mmol) was dissolved in distilled water (6 mL) and added to **4b** solution, and the reaction was left stirring for 6 h. The organic solvent was evaporated under reduced pressure, and the aqueous layer was acidified by using 1N HCl solution. The desired product was precipitated, filtered, and washed with distilled water; it was then dried under vacuum to obtain the desired product as white solid. ^1^H NMR (DMSO-d_6_) δ 1.28 (6H, d, CH_3_), 3.98 (3H, s, NCH_3_), 4.06 (1H, m, CH), 7.98 (1H, s, AR—H), 8.28 (1H, s, Ar—H); ^13^C NMR (DMSO-d_6_): δ 24.72, 26.90, 37.99, 110.44, 123.88, 129.73, 134.18, 134.92, 149.92, 153.42, 158.34, 163.54; (LC−MS (ESI): *m*/*z* calcd for C_13_H_14_N_4_O_5_S, 338.70, found 339.23 [M + H]^+^.

### 2.14. Synthesis of 2-(1-methyl-4-nitro-1H-pyrrole-2-carboxamido)-N(3(dimethylamino)propyl)-5-isopropylthiazole-4-carboxamide (***5***)

First, 2-(1-methyl-4-nitro-1H-pyrrole-2-carboxamido)-5-isopropylthiazole-4-carboxylate **3** (0.402 g, 1.163 mmol) was dissolved in 4 mL of dimethylaminopropylamine, and the reaction was left stirring overnight at 90 °C. The solvent was removed under reduced pressure, and basic water was added; the crude product was then precipitated, collected by filtration, and purified by silica column chromatography, using 1:98:1 MeOH/EA/TEA as mobile phase. Yield (90%); ^1^H NMR (CHOLOROFORM-d) δ 1.33 (6H, d, CH_3_), 1.8 (2H, m, CH_2_), 2.26 (6H, s, NCH_3_), 2.41 (2H, t, NCH_2_), 3.45 (2H, q, CONH--CH_2_), 4.10 (3H, d, NCH_3_), 4.41 (1H, m, CH), 7.55 (1H, t, CONH), 7.64 (1H, d, Ar—H), 7.70 (1H, d, Ar—H); ^13^C NMR (CHLOROFORM-d): δ 25.12, 27.23, 27.47, 37.71, 38.59, 45.61, 57.56, 109.71, 124.19, 128.38, 135.55, 136.07, 148.99, 152.78, 157.92, 162.86; LC−MS (ESI): *m*/*z* calcd for C_18_H_26_N_6_O_4_S, 422.17, found 423.08 [M + H]^+^.

### 2.15. Synthesis of N-(3-(dimethylamino)propyl)-5-isopropyl-2-(1-methyl-4-(1-methyl-4-(1-methyl-1H-imidazole-2-carboxamido)-1H-pyrrole-2-carboxamido)-1H-pyrrole-2-carboxamido)thiazole-4-carboxamide (MGB3)

First, 2-(1-methyl-4-nitro-1H-pyrrole-2-carboxamido)-*N*-(3-(dimethylamino)propyl)-5-isopropylthiazole-4-carboxamide (0.1593 g, 0.3773 mmol) was dissolved in methanol (2 mL) and THF (4 mL), and the solution then cooled to 0 °C. Then 10%-Pd/C (0.08 g) was added in small portions, and the flask was repeatedly evacuated and refilled with hydrogen gas; the suspension was stirred under hydrogen for 4 h. The suspension was then filtered, and the solvent was removed under reduced pressure to produce an oily amine product which was then diluted with 2 mL DMF and added dropwise to a solution of 1-methyl-4-(1-methyl-1H-imidazole-5-amido)-1H-pyrrole-2-carboxylic acid (0.078 mg, 0.31440 mmol), triethylamine (0.191 g, 1.8864 mmol), and HBTU (0.1431 g, 0.3773 mmol) in DMF (5 mL) and left stirring overnight, at room temperature, under nitrogen gas. The solvent was removed under reduced pressure, sodium bicarbonate solution (10 mL) was added, and the crude product was extracted twice (2 × 15 mL) with ethylacetate. The organic solvent was evaporated under reduced pressure, and the crude product was purified by reverse-phase HPLC, using a gradient elution method as described below. The product peaks were collected and freeze-dried to obtain the desired product in the form of a white solid. Yield (35%); ^1^H NMR (ACETIC ACID-d_4_) δ 1.32 (6H, d, CH_3_), 2.12 (2H, m, CH_2_), 3.02 (6H, s, NCH_3_), 3.31 (2H, t, NCH_2_), 3.49 (2H, q, CONH--CH_2_), 3.97 (3H, s, NCH_3_), 4.00 (3H, s, NCH_3_), 4.11 (3H, s, NCH_3_), 4.34 (1H, m, Ar—CH), 7.07 (1H, d, Ar—H), 7.09 (1H, d, Ar—H), 7.33 (1H, d, Ar—H), 7.35 (1H, d, Ar—H), 7.39 (1H, d, Ar—H), 7.49 (1H, d, Ar—H); ^13^C NMR (ACETIC ACID-d_4_): δ 15.30, 17.71, 26.28, 26.98, 27.34, 33.33, 46.07, 95.08, 97.47, 97.53, 109.76, 112.41, 114.43, 117.22, 126.70, 129.49, 137.79, 146.05, 149.83; LC−MS (ESI): *m*/*z* calcd for C_29_H_38_N_10_O_4_S, 622.28, found 623.27 [M + H]^+^.

HPLC method for the purification of MGB3.

**Time (min)**

**Flow Rate (mL/min)**

**% Water (0.1% TFA)**

**% Acetonitrile (0.1% TFA)**
0375252530100


### 2.16. Synthesis of N-(3-(dimethylamino)propyl)-5-isopropyl-2-(1-methyl-4-(1-methyl-4-(thiazole-4-carboxamido)-1H-pyrrole-2-carboxamido)-1H-pyrrole-2-carboxamido)thiazole-4-carboxamide (MGB4)

First, 2-(1-methyl-4-nitro-1H-pyrrole-2-carboxamido)-*N*-(3-(dimethylamino)propyl)-5-isopropylthiazole-4-carboxamide (0.1593 g, 0.37728 mmol) was dissolved in methanol (2 mL) and THF (4 mL), and the solution then cooled to 0 °C. Then 10%-Pd/C (0.08 g) was added in small portions, and the flask was repeatedly evacuated and refilled with hydrogen gas; the suspension was then stirred under hydrogen for 4 h. The suspension was filtered, and the solvent was removed under reduced pressure to produce an oily amine product which was then diluted with 2 mL DMF and added dropwise to a solution of 1-methyl-4-(1-methyl-1H-imidazole-5-amido)-1H-pyrrole-2-carboxylic acid (0.078 g, 0.31440 mmol), triethylamine (0.191 g, 1.8864 mmol), and HBTU (0.1431 mg, 0.37728 mmol) in DMF (5 mL) and left stirring overnight, at room temperature, under nitrogen gas. The solvent was removed under reduced pressure, sodium bicarbonate solution (10 mL) was added, and the crude product was extracted twice (2 × 15 mL) with ethyl acetate. The organic solvent was evaporated under reduced pressure, and the crude product was purified by reverse-phase HPLC, using a gradient elution method as described below. The product peaks were collected and freeze-dried to obtain the desired product in the form of a white solid. Yield (40%); ^1^H NMR (ACETIC ACID-d_4_) δ 1.32 (6H, d, CH_3_), 2.12 (2H, m, CH_2_), 3.03 (6H, s, NCH_3_), 3.32 (2H, t, NCH_2_), 3.49 (2H, q, CONH—CH_2_), 3.97 (3H, s, NCH_3_), 3.99 (3H, s, NCH_3_), 4.34 (1H, m, Ar—CH), 7.15 (1H, d, Ar—H), 7.41 (1H, d, Ar—H), 7.43 (1H, d, Ar—H), 7.48 (1H, d, Ar—H), 8.32 (1H, d, Ar—H), 9.10 (1H, d, Ar—H), 9.52 (1H, s, CONH), 9.68 (1H, s, CONH); ^13^C NMR (ACETIC ACID-d_4_): δ 15.14, 17.56, 26.04, 26.79, 27.15, 36.89, 45.93, 95.13, 97.42, 106.04, 108.35, 111.35, 112.65, 114.33, 126.47, 137.70, 142.24, 144.95, 148.55, 149.70, 149.78, 150.32, 154.38; LC−MS (ESI): *m*/*z* calcd for C_28_H_35_N_9_O_4_S_2_, 625.23, found 626.23 [M + H]^+^.

HPLC method for the purification of MGB4.

**Time (min)**

**Flow Rate (mL/min)**

**% Water (0.1% TFA)**

**% Acetonitrile (0.1% TFA)**
037525253595


### 2.17. Synthesis of N-(5-((5-((5-((3-(dimethylamino)propyl)carbamoyl)-1-methyl-1H-pyrrol-3-yl)carbamoyl)-1-methyl-1H-pyrrol-3-yl)carbamoyl)-1-methyl-1H-pyrrol-3-yl)-1-methyl-1H-imidazole-2-carboxamide (MGB5)

*N*-[3-(dimethylamino)propyl]-1-methyl-4-{[(1-methyl-4-nitro-1H-pyrrol-2-yl)carbonyl] amino}-1H-pyrrole-2-carboxamide (0.1819 g, 0.4836 mmol) was dissolved in methanol (2 mL) and THF (4 mL), and the solution then cooled to 0 °C. Then 10%-Pd/C (0.095 g) was added in small portions, and the flask was repeatedly evacuated and refilled with hydrogen gas; the suspension was stirred under hydrogen for 4 h. The suspension was then filtered, and the solvent was removed under reduced pressure to produce an oily amine product, which was diluted with 2 mL DMF and added dropwise to a solution of methyl 1-methyl-4-(1-methyl-1H-imidazole-5-amido)-1H-pyrrole-2-carboxylic acid **9** (0.100 g, 0.4031 mmol), triethylamine (0.2447 g, 2.4184 mmol), and HBTU (0.1834 g, 0.4836 mmol) in DMF (2 mL) and left stirring overnight, at room temperature, under nitrogen gas. The solvent was removed under reduced pressure, sodium bicarbonate solution (10 mL) was added, and the crude product was extracted twice (2 × 15 mL) with ethyl acetate. The organic solvent was evaporated under reduced pressure, and the crude product was purified by reverse-phase HPLC, using a gradient elution method, as described below. The product peaks were collected and freeze-dried to obtain the desired product as a yellow solid. Yield (41%); ^1^H NMR (ACETIC ACID-d_4_) δ 2.11 (2H, m, CH_2_), 3.04 (6H, s, NCH_3_), 3.33 (2H, t, NCH_2_), 3.36 (6H, s, NCH_3_), 3.49 (2H, q, CONH—CH_2_), 3.90 (3H, s, NCH_3_), 3.93 (3H, s, NCH_3_), 3.96 (3H, s, NCH_3_), 4.13 (1H, s, NH) 6.93 (1H, d, Ar—H), 6.97 (1H, d, Ar—H), 7.04 (1H, d, Ar—H), 7.23 (1H, d, Ar—H), 7.26 (1H, d, Ar—H), 7.34 (1H, d, Ar—H), 7.83 (1H, s, CONH), 9.26 (1H, s, CONH), 9.34 (1H, s, CONH); ^13^C NMR (ACETIC ACID-d_4_): δ 16.21, 20.81, 20.90, 26.30, 26.54, 26.92, 27.01, 27.10, 33.40, 46.02, 95.02, 95.38, 109.79, 112.63, 113.78, 113.95, 114.32, 114.77, 116.53, 117.42, 145.87, 149.78, 149.92, 154.01; LC−MS (ESI): *m*/*z* calcd for C_27_H_38_N_10_O_4_, 576.29, found 577.33 [M + H]^+^.

HPLC method for the purification of MGB5.

**Time (min)**

**Flow Rate (mL/min)**

**% Water (0.1% TFA)**

**% Acetonitrile (0.1% TFA)**
039010253595


### 2.18. Synthesis of N-(5-((5-((5-((3-(dimethylamino)propyl]carbamoyl)-1-methyl-1H-pyrrol-3-yl)carbamoyl)-1-methyl-1H-pyrrol-3-yl}carbamoyl)-1-methyl-1H-pyrrol-3-yl)-1,3-thiazole-4-carboxamide (MGB6)

*N*-[3-(dimethylamino)propyl]-1-methyl-4-{[(1-methyl-4-nitro-1H-pyrrol-2-yl)carbonyl] amino}-1H-pyrrole-2-carboxamide **7** (0.2823 g, 0.750 mmol) was dissolved in methanol (2 mL) and THF (4 mL), and the solution was then cooled to 0 °C. Then 10%-Pd/C (0.141 g) was added in small portions, and the flask was repeatedly evacuated and refilled with hydrogen gas; the suspension was stirred under hydrogen for 4 h. The suspension was then filtered, and the solvent was removed under reduced pressure to produce an oily amine product, which was diluted with 2 mL DMF and added dropwise to a solution of methyl 1-methyl-4-(1,3-thiazole-4-amido)-1H-pyrrole-2-carboxylic acid **10** (0.157 g, 0.6254 mmol), triethylamine (0.3830 g, 3.785 mmol), and HBTU (0.2846 g, 0.750 mmol) in DMF (2 mL) and left stirring overnight, at room temperature, under nitrogen gas. The solvent was removed under reduced pressure, sodium bicarbonate solution (10 mL) was added, and the crude product was extracted twice (2 × 15 mL) with ethyl acetate. The organic solvent was evaporated under reduced pressure, and the crude product was purified by reverse-phase HPLC, using a gradient elution method, as described below. The product peaks were collected and freeze-dried to obtain the desired product as a white solid. Yield (31%); ^1^H NMR (ACETIC ACID-d_4_) δ 2.08 (2H, m, CH_2_), 2.99 (6H, s, NCH_3_), 3.30 (2H, t, NCH_2_), 3.45 (2H, m, CONH--CH_2_), 3.89 (3H, s, NCH_3_), 3.94 (3H, s, NCH_3_), 3.97 (3H, s, NCH_3_), 6.89 (1H, s, Ar—H), 6.97 (1H, s, Ar—H), 7.09 (1H, d, Ar—H), 7.15 (1H, d, Ar—H), 7.23 (1H, s, Ar—H), 7.27 (1H, d, Ar—H), 7.39 (1H, s, Ar—H), 7.41 (1H, s, Ar—H), 8.32 (1H, s, CONH), 8.34 (1H, s, CONH), 9.10 (1H, s, CONH), 9.11 (1H, s, CONH); (CHLOROFORM-d): δ 25.19, 36.62, 36.78, 36.97, 34.22, 104.10, 104.37, 104.50, 111.11, 118.46, 119.69, 121.14, 122.06, 123.43, 123.52, 124.54, 125.10, 127.76, 132.23, 143.48, 150.88, 153.11, 158.33, 159.17, 162.72; LC−MS (ESI): *m*/*z* calcd for C_27_H_33_N_9_O_4_S, 579.24, found 580.32 [M + H]^+^.

HPLC method for the purification of MGB6.

**Time (min)**

**Flow Rate (mL/min)**

**% Water (0.1% TFA)**

**% Acetonitrile (0.1% TFA)**
039010253595


### 2.19. General Procedure for the Synthesis of MGB16, MGB20, MGB22, MGB24, MGB26, MGB28, MGB30, and MGB32

A variety of carboxylic acid compounds (stated below) were coupled with *N*-[3-(dimethylamino)propyl]-1-methyl-4-{[(1-methyl-4-nitro-1H-pyrrol-2-yl)carbonyl] amino}-1H-pyrrole-2-carboxamide **7** or 1-methyl-4-(1-methyl-4-nitro-1H-pyrrole-2-carboxamido)-*N*-(3-morpholinopropyl)-1H-pyrrole-2 carboxamide **8** to generate the final product. Equimolar amounts of **7** or **8** were dissolved in methanol (2 mL) and THF (4 mL), and the solution then cooled to 0 °C. Then 10%-Pd/C (0.1 g) was added in small portions, the flask was repeatedly evacuated and refilled with hydrogen gas, and the suspension was stirred under hydrogen for 4 h. The suspension was then filtered, and the solvent was removed under reduced pressure to produce an oily amine product which was then diluted with 1 mL DMF and added dropwise to a solution containing equimolar amount the acid, triethylamine (6 equivalent), and HBTU (1.1 equivalent) in DMF (1 mL) and left stirring overnight, at room temperature, under nitrogen gas. The solvent was removed under reduced pressure, sodium bicarbonate solution (10 mL) was added, and the crude product was extracted twice (2 × 15 mL) with ethyl acetate. The organic solvent was evaporated under reduced pressure, and the crude product was purified by reverse-phase HPLC, using a gradient elution method, as described below. The product peaks were collected and freeze-dried to obtain the desired product as solid TFA salts.

### 2.20. Synthesis of 1-methyl-N-(1-methyl-5-{[3-(morpholin-4-yl)propyl]carbamoyl}-1H-pyrrol-3-yl)-4-(2-oxo-2H-chromene-3-amido)-1H-pyrrole-2-carboxamide (MGB16)

Reactants: coumarin-3-carboxylic acid and **8**. Purified by silica column chromatography. Yield (55%); ^1^H NMR (ACETIC ACID-d_4_) δ 2.18 (2H, m, CH_2_), 3.32 (4H, t, NCH_2_), 3.43 (2H, m, NCH_2_), 3.59 (2H, q, CONH--CH_2_), 3.75 (4H, d, OCH_2_), 3.93 (3H, s, NCH_3_), 3.98 (3H, s, NCH_3_), 6.95 (1H, d, Ar—H), 7.04 (1H, d, Ar—H), 7.30 (1H, d, Ar—H), 7.44 (1H, d, Ar—H), 7.49 (1H, s, Ar—H), 7.50 (1H, d, Ar—H), 7.81 (1H, t, Ar—H), 8.00 (1H, d, Ar—H), 8.08 (1H, t, Ar—H), 8.98 (1H, s, CONH), 9.26 (1H, s, CONH), 10.57 (1H, s, CONH); ^13^C NMR (DMSO-d_6_): δ 8.48, 30.54, 35.89, 36.11, 45.61, 51.05, 54.05, 63.37, 104.27, 116.08, 118.41, 119.24, 122.44, 123.08, 125.15, 130.10, 134.02, 146.86, 153.66, 175.95, 158.04, 160.58, 161.49; LC−MS (ESI): *m*/*z* calcd for C_29_H_32_N_6_O_6_, 560.24, found 561.27 [M + H]^+^.

### 2.21. Synthesis of 1-methyl-4-(1-methyl-4-(3-(naphthalen-2-ylthio)propanamido)-1H-pyrrole-2-carboxamido)-N-(3-morpholinopropyl)-1H-pyrrole-2-carboxamide (MGB20)

Reactants: coumarin-3-carboxylic acid and **8**. Yield (31%); ^1^H NMR (ACETIC ACID-d_4_) δ, 2.14 (2H, m, CH_2_), 2.76 (2H, t, SCH_2_), 3.22 (4H, t, NCH_2_), 3.35 (2H, t, NCH_2_), 3.39 (2H, t, NHCO--CH_2_), 3.49 (2H, q, CONH--CH_2_), 3.66 (4H, t, OCH_2_), 3.90 (3H, s, NCH_3_), 3.90 (3H, s, NCH_3_), 6.80 (1H, d, Ar—H), 6.91 (1H, s, Ar—H), 7.13 (1H, d, Ar—H), 7.26 (1H, s, Ar—H), 7.46 (1H, m, Ar—H), 7.48 (1H, d, Ar—H), 7.51 (1H, t, Ar—H), 7.86 (4H, q, Ar—H), 9.11 (1H, s, CONH), 9.20 (1H, s, CONH); ^13^C NMR (DMSO-d_6_): δ 8.48, 30.54, 35.89, 36.11, 45.61, 51.05, 54.05, 63.37, 104.27, 116.08, 118.41, 119.24, 120.62, 123.08, 125.15, 130.10, 134.02, 146.86, 153.66, 175.95, 158.04, 160.58, 161.49; LC−MS (ESI): *m*/*z* calcd for C_32_H_38_N_6_O_4_S, 602.27, found 603.29 [M + H]^+^.

HPLC method for the purification of MGB20.

**Time (min)**

**Flow Rate (mL/min)**

**% Water (0.1% TFA)**

**% Acetonitrile (0.1% TFA)**
0375252532575


### 2.22. Synthesis of N-(3-(dimethylamino)propyl)-1-methyl-4-(1-methyl-4-(3-((4-methylbenzyl)thio)propanamido)-1H-pyrrole-2-carboxamido)-1H-pyrrole-2-carboxamide (MGB22)

Reactants: 4-Methylbenzyl-3-thio-propionic acid and 7. Yield (55%); ^1^H NMR (ACETIC ACID-d_4_) δ 2.11 (2H, m, CH_2_), 2.28 (3H, s, CH_3_), 2.54 (2H, t, SCH_2_), 2.71 (2H, t, NHCO—CH_2_), 3.33 (2H, s, NCH_2_), 3.49 (2H, q, CONH--CH_2_), 3.72 (2H, s, SCH_2_—Ar), 3.89 (3H, s, NCH_3_), 3.90 (3H, s, NCH_3_), 6.81 (1H, s, Ar—H), 6.91 (1H, s, Ar—H), 7.10 (1H, s, Ar—H), 7.12 (1H, s, Ar—H), 7.21 (2H, d, Ar—H), 7.24 (1H, s, Ar--H), 7.84 (1H, s, CONH), 9.04 (1H, d, CONH), 9.19 (1H, d, CONH); ^13^C NMR (ACETIC ACID-d_4_): δ 11.22, 16.12, 17.83, 26.04, 26.32, 27.17, 33.29, 45.84, 94.44, 95.30, 113.32, 113.43, 114.14, 119.87, 119.99, 126.77, 127.27, 149.68, 154.01, 158.79; LC−MS (ESI): *m*/*z* calcd for C_28_H_38_N_6_O_3_S, 538.27, found 539.42 [M + H]^+^.

HPLC method for the purification of MGB22.

**Time (min)**

**Flow Rate (mL/min)**

**% Water (0.1% TFA)**

**% Acetonitrile (0.1% TFA)**
057030255455525.250100285010028.2570303257030


### 2.23. Synthesis of 1-methyl-4-(1-methyl-4-(3-(p-tolylthio)propanamido)-1H-pyrrole-2-carboxamido)-N-(3-morpholinopropyl)-1H-pyrrole-2-carboxamide (MGB24)

Reactants: 4-Methylbenzyl-3-thio-propionic acid and **8**. Yield (21%); ^1^H NMR (ACETIC ACID-d_4_) δ, 2.14 (2H, m, CH_2_), 2.28 (3H, s, CH_3_), 2.54 (2H, t, SCH_2_), 2.71 (2H, t, NHCO--CH_2_), 3.22 (2H, t, NCH_2_), 3.36 (2H, t, NCH_2_), 3.49 (2H, q, CONH--CH_2_), 3.67 (2H, d, NCH_2_), 3.72 (2H, s, SCH_2_—Ar), 3.89 (3H, s, NCH_3_), 3.90 (3H, s, NCH_3_), 3.94 (2H, d, OCH_2_), 4.08 (2H, d, OCH_2_), 6.80 (1H, s, Ar—H), 6.91 (1H, s, Ar—H), 7.10 (1H, s, Ar—H), 7.12 (1H, s, Ar—H), 7.21 (1H, s, Ar—H), 7.23 (1H, s, Ar—H), 7.25 (1H, s, Ar—H), 7.86 (1H, s, CONH), 9.04 (1H, s, CONH), 9.18 (1H, s, CONH); ^13^C NMR (ACETIC ACID-d_4_): δ 11.07, 14.85, 17.67, 26.17, 26.60, 27.01, 27.06, 42.48, 45.05, 54.72, 94.30, 95.22, 108.98, 113.12, 113.26, 113.98, 119.72, 119.84, 119.98, 126.61, 127.12, 149.54, 153.87, 158.65; LC−MS (ESI): *m*/*z* calcd for C_30_H_40_N_6_O_4_S, 580.28, found 581.45 [M + H]^+^.

HPLC method for the purification of MGB24.

**Time (min)**

**Flow Rate (mL/min)**

**% Water (0.1% TFA)**

**% Acetonitrile (0.1% TFA)**
058020255507025.2509028509028.2580203258020


### 2.24. Synthesis of N-(3-(dimethylamino)propyl)-1-methyl-4-(1-methyl-4-(4(phenylethynyl)benzamido)-1H-pyrrole-2-carboxamido)-1H-pyrrole-2-carboxamide (MGB26)

Reactants: 4-(phenylethynyl)benzoic acid and **7**. Yield (35%); ^1^H NMR (ACETIC ACID-d_4_) δ 2.94 (6H, s, NCH_3_), 3.27 (2H, t, NCH_2_), 3.45 (2H, q, CONH--CH_2_), 3.89 (3H, s, NCH_3_), 3.95 (3H, s, NCH_3_), 6.87 (1H, s, Ar—H), 7.02 (1H, s, Ar—H), 7.27 (1H, d, Ar—H), 7.34 (1H, d, Ar—H), 7.43 (2H, d, Ar—H), 7.44 (1H, d, Ar—H), 7.57 (2H, m, Ar--H), 7.64 (2H, d, Ar—H), 7.98 (2H, d, Ar—H), 9.25 (1H, s, CONH), 9.71 (1H, s, CONH); ^13^C NMR (ACETIC ACID-d_4_): δ 15.94, 26.92, 27.03, 33.18, 46.04, 79.70, 82.37, 95.06, 95.18, 109.53, 109.94, 113.64, 113.79, 113.95, 117.05, 118.70, 119.86, 122.59, 122.75, 125.87, 149.84, 153.38, 154.10; LC−MS (ESI): *m*/*z* calcd for C_32_H_34_N_6_O_3_, 550.27, found 551.25 [M + H]^+^.

HPLC method for the purification of MGB26.

**Time (min)**

**Flow Rate (mL/min)**

**% Water (0.1% TFA)**

**% Acetonitrile (0.1% TFA)**
058020255554525.250100285010028.2580203258020


### 2.25. Synthesis of 1-methyl-4-(1-methyl-4-(4-(phenylethynyl)benzamido)-1H-pyrrole-2-carboxamido)-N-(3-morpholinopropyl)-1H-pyrrole-2-carboxamide (MGB28)

Reactants: 4-(phenylethynyl)benzoic acid and **8**. Yield (46%); ^1^H NMR (ACETIC ACID-d_4_) δ 2.10 (2H, m, CH_2_), 3.16 (4H, s, NCH_2_), 3.29 (2H, t, NCH_2_), 3.46 (2H, q, CONH—CH_2_), 3.58 (2H, s, OCH_2_), 3.89 (3H, s, NCH_3_), 3.95 (3H, s, NCH_3_), 4.00 (2H, s, OCH_2_), 6.88 (1H, s, Ar—H), 7.01 (1H, s, Ar—H), 7.27 (1H, d, Ar—H), 7.33 (1H, s, Ar—H), 7.43 (2H, d, Ar—H), 7.44 (1H, d, Ar—H), 7.57 (2H, m, Ar--H), 7.64 (2H, d, Ar—H), 7.98 (2H, d, Ar—H), 9.25 (1H, s, CONH), 9.70 (1H, d, CONH); ^13^C NMR (ACETIC ACID-d_4_): δ10.99, 14.69, 26.49, 26.65, 26.69, 27.01, 42.41, 45.38, 54.55, 79.38, 82.07, 94.80, 94.85, 103.79, 109.29, 109.61, 113.32, 113.43, 113.63, 116.75, 118.38, 119.56, 122.29, 122.44, 125.55, 128.94, 149.53, 153.14, 153.79; LC−MS (ESI): *m*/*z* calcd for C_34_H_36_N_6_O_4_, 592.28, found 593.49 [M + H]^+^.

HPLC method for the purification of MGB28.

**Time (min)**

**Flow Rate (mL/min)**

**% Water (0.1% TFA)**

**% Acetonitrile (0.1% TFA)**
058020255505025.250100285010028.2580203258020


### 2.26. Synthesis of (E)-N-(3-(dimethylamino)propyl)-1-methyl-4-(1-methyl-4-(4-styrylbenzamido)-1H-pyrrole-2-carboxamido)-1H-pyrrole-2-carboxamide (MGB30)

Reactants: 4-stilbenecarboxylic acid and **7**. Yield (48%); ^1^H NMR (ACETIC ACID-d_4_) δ 2.08 (2H, m, CH_2_), 2.95 (6H, s, NCH_3_), 3.26 (2H, t, NCH_2_), 3.44 (2H, q, CONH--CH_2_), 3.89 (3H, s, NCH_3_), 3.95 (3H, s, NCH_3_), 6.88 (1H, s, Ar—H), 7.02 (1H, s, Ar—H), 7.28 (2H, d, Ar—H), 7.30 (1H, d, Ar—H), 7.35 (2H, m, Ar—H), 7.37 (1H, s, HC = CH), 7.38 (1H, d, HC = CH), 7.63 (2H, d, Ar—H), 7.71 (2H, d, Ar—H), 7.97 (2H, d, Ar—H), 9.27 (1H, d, CONH), 9.67 (1H, d, CONH); ^13^C NMR (ACETIC ACID-d_4_): δ 15.65, 26.28, 26.62, 26.67, 32.89, 45.74, 94.79, 94.89, 113.46, 113.48, 114.20, 117.26, 117.61, 119.62, 121.29, 124.67, 128.06, 131.21, 149.57, 153.13, 154.22; LC−MS (ESI): *m*/*z* calcd for C_32_H_36_N_6_O_3_, 552.28, found 553.29 [M + H]^+^.

HPLC method for the purification of MGB30.

**Time (min)**

**Flow Rate (mL/min)**

**% Water (0.1% TFA)**

**% Acetonitrile (0.1% TFA)**
057030255455525.250100285010028.2570303257030


### 2.27. Synthesis of (E)-1-methyl-4-(1-methyl-4-(4-styrylbenzamido)-1H-pyrrole-2-carboxamido)-N-(3-morpholinopropyl)-1H-pyrrole-2-carboxamide (MGB32)

Reactants: 4-stilbenecarboxylic acid and **8**. Yield (36%); ^1^H NMR (ACETIC ACID-d_4_) δ 2.11 (2H, m, CH_2_), 3.10 (4H, s, NCH_2_), 3.30 (2H, t, NCH_2_), 3.46 (2H, q, CONH—CH_2_), 3.59 (2H, s, OCH_2_), 3.89 (3H, s, NCH_3_), 3.95 (3H, s, NCH_3_), 4.01 (2H, s, OCH_2_), 6.88 (1H, s, Ar—H), 7.01 (1H, s, Ar—H), 7.27 (1H, s, Ar—H), 7.31 (1H, d, Ar—H), 7.33 (2H, s, Ar—H), 7.36 (1H, d, Ar—H), 7.39 (1H, s, HC = CH), 7.40 (1H, d, HC = CH), 7.62 (2H, d, Ar--H), 7.71 (2H, d, Ar—H), 7.97 (2H, d, Ar—H), 9.25 (1H, s, CONH), 9.60 (1H, s, CONH); ^13^C NMR (ACETIC ACID-d_4_): δ 14.84, 26.64, 26.81, 26.83, 42.57, 45.54, 54.70, 94.96, 95.00, 113.62, 113.63, 114.36, 117.42, 117.77, 119.78, 121.46, 124.83, 128.21, 131.38, 149.72, 153.31, 154.36; LC−MS (ESI): *m*/*z* calcd for C_34_H_38_N_6_O_4_, 594.30, found 595.44 [M + H]^+^.

HPLC method for the purification of MGB32.

**Time (min)**

**Flow Rate (mL/min)**

**% Water (0.1% TFA)**

**% Acetonitrile (0.1% TFA)**
057030255455525.250100285010028.2570303257030


### 2.28. Acanthamoeba Culturing

*Acanthamoeba castellanii* genotype T4 (ATCC 50492) was cultivated in 10 mL growth medium of peptone yeast glucose (PYG) in tissue flasks (Baig, et al., 2021; Anwar, et al., 2019). The growth medium, PYG, consists of 0.75% proteose peptone, 0.75% yeast extract, and 1.5% glucose. Next, the amoebae were kept in tissue flasks and incubated at 30 °C. After 48 h, the flasks were found to reach approximately 90% confluency, and the amoebae were utilized in experiments [18].

### 2.29. Henrietta Lacks (HeLa) Cervical Cancer Cells

HeLa cells were acquired from American Type Culture Collection (ATCC) of identification ATCC CCL-2, Singapore. The cells are utilized to conduct cytotoxicity and cytopathogenicity assays [19]. Through their cultivation in complete media, the cells were grown, and then they were placed in a 95% humidifying incubator at 37 °C and 5% CO_2_. Complete medium comprises Roswell Park Memorial Media (RPMI), 1% penicillin–streptomycin, 1% minimum essential medium amino acids, 1% L-glutamine, and 10% fetal bovine serum (FBS) [19].

### 2.30. Amoebicidal Assay

To determine the compounds’ antiamoebic effects, amoebicidal assays were conducted. The 5 × 10^5^ amoebae were placed in a 24-well plate and brought to a final volume of 500 µL. The amoebae were then treated with the compounds at 50 µM. The 24-well plate was then placed in a 30 °C incubator for 24 h. Positive and negative controls were added to the assay in order to ensure reliable results. Serving as the negative control was the amoebae alone, with RPMI [4]. Meanwhile, amoebae, along with 0.25% sodium dodecyl sulphate (SDS), were used as the positive control. Through the addition of 0.1% methylene blue, live and dead amoebae were distinguished. Additionally, significant compounds were identified by conducting a Student’s *t*-test with a two-tailed distribution [19]. Furthermore, the MIC_50_ values of three compounds were determined by using concentrations of 50, 75, and 100 µM.

### 2.31. Encystation Assay

The effects of the compounds on amoeba encystation were determined through the conduction of encystation assays. Briefly, 1 × 10^6^ trophozoites were incubated with the compounds in a final concentration of 16% filter-sterilized glucose [4]. Following a 48 h incubation period, 0.1% SDS was added per well for 20 min. The cysts were then counted by using a haemocytometer. To ensure accuracy, *Acanthamoeba castellanii* was cultured in 16% glucose, serving as the negative control.

### 2.32. Excystation Assay

Amoeba culture was suspended in 3 mL phosphate-buffered saline (PBS) and placed on non-nutrient bacteriological agar plates to allow for the growing of *Acanthamoeba castellanii* cysts [4]. After two weeks, PBS was applied to plates containing amoeba cysts, which were then scraped off. The amoebae were centrifuged at 3000× *g* for 10 min, and the resulting pellet was resuspended in RPMI. The 5 × 10^5^ cysts were then incubated in PYG, along with 50 µM of the compounds. To ensure accuracy, amoeba cysts in PYG alone were considered as the negative control, whereas amoeba cysts in RPMI alone were taken as the positive control, as the cysts would not be able to revert back to trophozoites. The amoeba trophozoites were then numbered by using a haemocytometer.

### 2.33. Cytotoxicity Assay

To determine the toxicity of the compounds against human HeLa cells, cytotoxicity assays were conducted. In short, HeLa cells were cultured in 96-well plates and treated with the compounds. The plate was then placed in a humidifying incubator with 95% humidity and 5% CO_2_, at 37 °C, for 24 h [19]. After 24 h, the supernatant was collected, and the cytotoxicity of the compounds was determined through a cytotoxicity detection kit that measured the quantity of lactate dehydrogenase (LDH) release [20]. Accurate results were ensured through the addition of positive and negative controls. Untreated HeLa cells served as the negative control, whereas HeLa cells treated with 1% Triton X-100 served as the positive control. Finally, the cytotoxic properties were numerically determined by following the necessary calculations: (absorbance of media from cells treated with the drugs—absorbance of media from cells of negative control)/(absorbance of media from cells of positive control—absorbance of media from cells of negative control) × 100 [19].

### 2.34. Cytopathogenicity Assay

Amoeba-mediated host-cell death was determined by treating 5 × 10^5^ amoebae with 50 µM of compounds. The amoeba-containing plate was then placed in the incubator for 2 h at 30 °C. Following the 2 h incubation period, the treated amoebae were put on confluent HeLa cell monolayers [19]. The plate containing the treated amoebae and HeLa cells was incubated for 24 h [19]. Progressing the incubation period, the supernatant was collected, and the cytotoxicity was determined [19]. Accurate results were ensured through the addition of negative and positive controls. The untreated amoebae, alone, on the cells served as the negative control, while the cells treated with Triton X-100 served as the positive control [19].

### 2.35. Statistical Analysis

The data presented are illustrative of the mean ± standard error of at least three different independent experiments. A two-tailed distribution *t*-test was conducted to determine the statistical significance of the results. Additionally, *p*-values were determined to further examine and elaborate the results [19].

## 3. Results

### 3.1. Chemical Synthesis

**Scheme 1:**
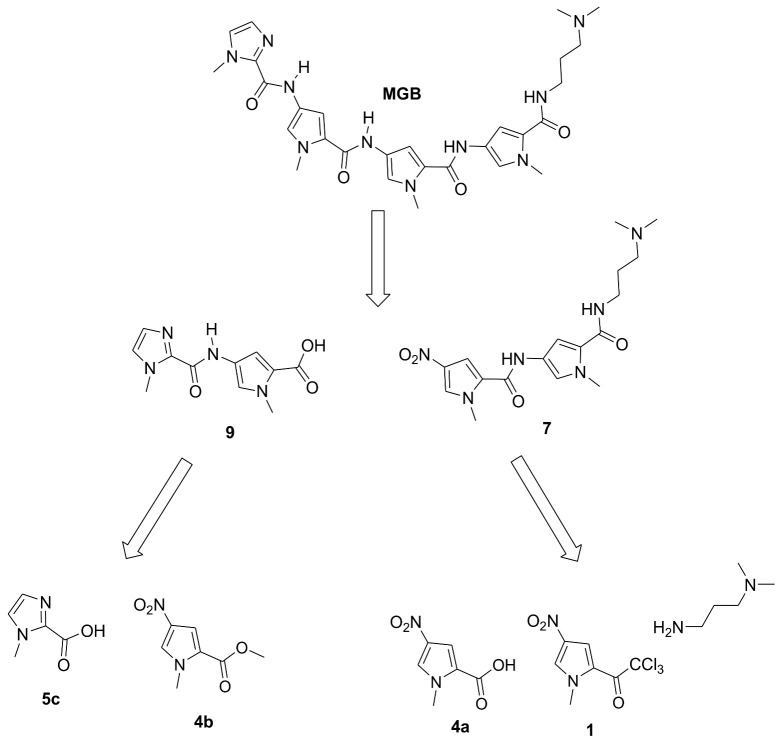
Retrosynthetic analysis of an amide-linked minor groove binder.

Twelve structurally related DNA minor groove binders were prepared by using the convergent synthesis that is illustrated in the retrosynthetic planning (Scheme 1) for one of the MGBs as an example. This compound was prepared through the coupling of imidazole containing carboxylic acid **9** with compound **7** after the reduction of the nitro group. Compound **9** was prepared by coupling the acid **5c** with **4b** after the reduction of its nitro group followed by the hydrolysis of the methyl ester. Compound **7** was generated by reacting the dimethylaminopropyl amine tail with the trichloroactyl group in 1 (a good leaving group), followed by the reduction of its nitro group and subsequent coupling with acid **4a**. 

#### 3.1.1. Synthesis of Isopropylthiazole Containing Intermediates

**Scheme 2:**
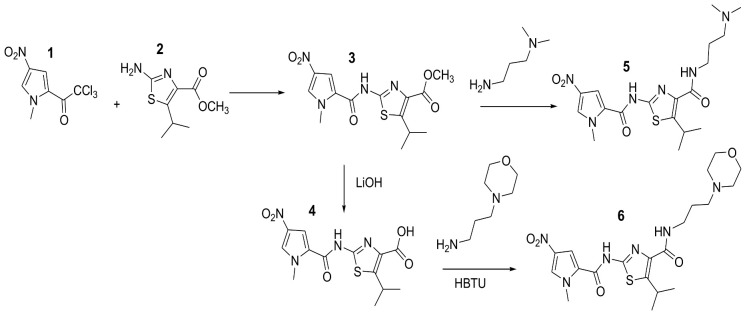
Synthesis of isopropylthiazole containing intermediates (**5** and **6**) with dimethylaminopropyl and morpholinepropyl tails.

Compounds **1** and **2** (Scheme 2) were prepared as before [8] and reacted under anhydrous conditions to produce **3** in a good yield (70%). The methyl ester of **3** was easily removed by aminolysis through refluxing **3** with dimethylaminopropylamine to produce the major intermediate **5** in an excellent yield (90%). The aminolysis reaction was not successful, using 3-morpholinopropylamine with the methyl ester (**3**); therefore, **3** was hydrolyzed by using lithium hydroxide to generate the carboxylic acid intermediate (yield 85%), which was then coupled with 3-morpholinopropylamine, using HBTU to produce the major intermediate **6** in a very good yield (80%).

#### 3.1.2. Synthesis of N-Methylpyrrole Containing Intermediates

**Scheme 3:**
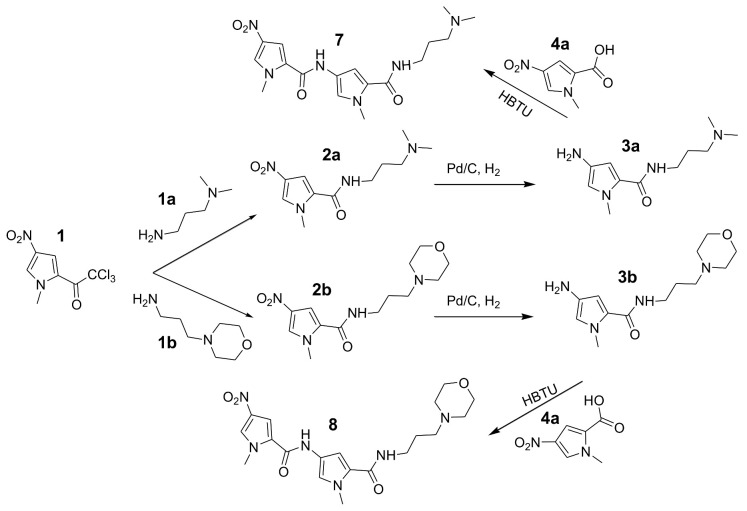
Synthesis of *N*-methylpyrrole containing intermediates (**7** and **8**) with dimethylaminopropyl and 3-morpholinopropylamine tails.

The trichloroacetyl moiety in **1** (Scheme 3) is a good leaving group; it was therefore directly reacted with dimethylaminopropylamine (**2a**) and 3-morpholinopropylamine (**2b**) to generate the intermediates **2a** and **2b**, respectively, in a good yield (75%). The two intermediates were then reduced by catalytic hydrogenation to generate the amino intermediates **3a** and **3b**. These intermediates were not isolated, due to the lack of stability and coupled directly after the reduction reaction was finished with 4-nitropyrrole carboxylic acid (**4a**) to generate the major intermediates **7** and **8** with dimethylaminopropylamine and 3-morpholinopropylamine tails, respectively.

#### 3.1.3. Synthesis of Imidazole and Thiazole Containing Intermediates

**Scheme 4:**
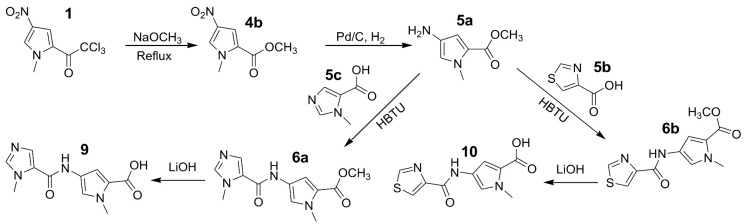
Synthesis of imidazole and thiazole containing intermediates (**9** and **10**) with carboxylic acid functional groups.

First, 2-trichloroacetyl-*N*-methylpyrrole **1** (Scheme 4) was reacted with sodium methoxide under reflux conditions to generate **4b** in a good yield (75%), which was then reduced by catalytic hydrogenation to generate the amino intermediate **5a**, which was directly coupled with imidazole carboxylic acid 5c and thiazole carboxylic acid, using HBTU to generate the intermediates **6a** (**60%**) and **6b** (**65%**), respectively. The methyl esters of these intermediates were then hydrolyzed by lithium hydroxide to produce the major carboxylic acid intermediates **9** and **10** in 90% and 85% yield, respectively (Scheme 4).

#### 3.1.4. Synthesis of MGB Final Products

The major intermediate **5** was reduced by catalytic hydrogenation with 10%-Pd/C in hydrogen gas and then directly coupled by using HBTU with the carboxylic acid intermediates **9** and **10** to obtain the final products MGB3 and MGB4 in 35% and 40% yield, respectively (Table 1). The nitro group of intermediate **7** was reduced by using 10%-Pd/C in hydrogen gas to give the amine, which was coupled with the carboxylic acid intermediates **9** and **10** and other commercially available carboxylic acids, namely 4-methylbenzyl-3-thio-propionic acid, 4-phenylethynylbenzoic acid, and 4-stilbenecarboxylic acid, to generate the final MGB products MGB5 (41%), MGB6 (27%), MGB22 (55%), MGB26 (35%), and MGB30(48%), respectively, after HPLC purification and lyophilization. The remaining final products were prepared by the reduction of intermediate **8**, using 10%-Pd/C in hydrogen gas to produce the amine, which was directly coupled by using HBTU with the following commercially available carboxylic acids: coumarin-3-carboxylic acid, 3-(2-naphthylthio)propionic acid, 4-methylbenzyl-3-thio-propionic acid, 4-phenylethynylbenzoic acid, and 4-stilbenecarboxylic acid to obtain the final MGB products MGB16 (55%), MGB20 (31%), MGB24 (21%), MGB28 (46%), and MGB32(36%), respectively, after being HPLC purified and subjected to lyophilization. Partition coefficient (log P) and pk_a_ (−log K_a_) values were calculated by using the Marvin application (https://www.chemaxon.com (accessed on 15 May 2022); see Table 1). Log P measures the molecule’s overall hydrophobicity, which is relevant to hydrophobic binding interactions with the target and, more important, relates to the absorption and the ability of molecule to cross lipophilic membranes. Log D measures the hydrophobicity of the molecule at a specific pH, which is affected by the ionization of the molecule if it possesses ionizable acidic or basic groups. All the prepared MGBs possess at least one basic amino group, which is ionized at physiological pH (7.4), and the degree of ionization is dictated by the pk_a_ of the ionizable moieties; for instance, the pk_a_ of MGBs with dimethylaminopropylamine tail is 9.3, while the pk_a_ of MGBs with morpholinepropylamine is 7.03. Therefore, all MGBs with dimethylaminopropylamine tail exist in the ionized state at physiological pH, and that was reflected on their Log D values (at pH 7.4), which were substantially decreased compared to the MGBs with morpholinepropylamine moiety. Moreover, the presence of alkene- or alkyne-linked phenyls in MGBs (e.g., MGBs: 26, 28, 30, and 32) tremendously increased their Log P and Log D values, which also might contribute to the ligand’s absorption and activity.

### 3.2. Bioassays

#### 3.2.1. MGB Compounds Were Evaluated for Amoebicidal Effects against *Acanthamoeba castellanii*

**Figure 1:**
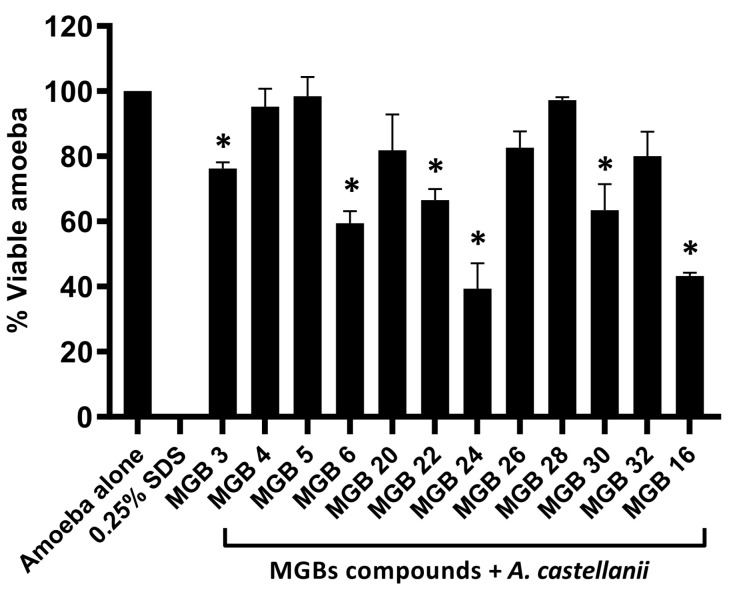
MGB3, MGB6, MGB22, MGB24, and MGB16 showed significant amoebicidal effects against *Acanthamoeba castellanii*. Data are illustrative of independent experiments pursued and are presented with a mean ± standard error. In addition, *p*-values were determined through the conduction of a two-sample *t*-test, two-tailed distribution; * is *p* ≤ 0.05.

The antiamoebic effects of the compounds were determined through the conduction of amoebicidal assays against *A. castellanii.* The amoeba was treated with 50 µM of the compounds and incubated overnight. The following day, the compounds exhibiting significant (*t*-test, two-tail distribution, *p* ≤ 0.05) amoebicidal activity were determined. Significant amoebicidal activity was recorded for the compounds MGB3, MGB6, MGB22, MGB24, and MGB16 (Figure 1). In the negative control, amoeba viability was found to be 100%; however, the compounds MGB3, MGB6, MGB22, MGB24, and MGB16 were found to significantly reduce amoeba viability to 76.20%, 59.45%, 66.5%, 39.32%, and 43.21%, respectively (Figure 1). The remaining compounds, MGB4, MGB5, MGB20, MGB26, MGB28, MGB30, and MGB32, did not exhibit any significant reductions; these compounds were found to reduce amoeba viability from 100% (according to the negative control) to 95.25%, 98.42%, 81.88%, 82.67%, 97.2%, 63.41%, and 80.08%, respectively (Figure 1).

Additionally, the minimum inhibitory concentration required to inhibit 50% of amoeba growth (MIC_50_) was determined to be 65.91, 58.34, and 83.62 µM for the compounds MGB3, MGB6, and MGB20 respectively (Table 2). The MIC_50_ of the compounds was determined through amoebicidal assays conducted against different concentrations of the compounds: 50, 75, and 100 µM.

#### 3.2.2. MGB Compounds Were Assessed for Their Effects on *A. castellanii* Encystation

**Figure 2:**
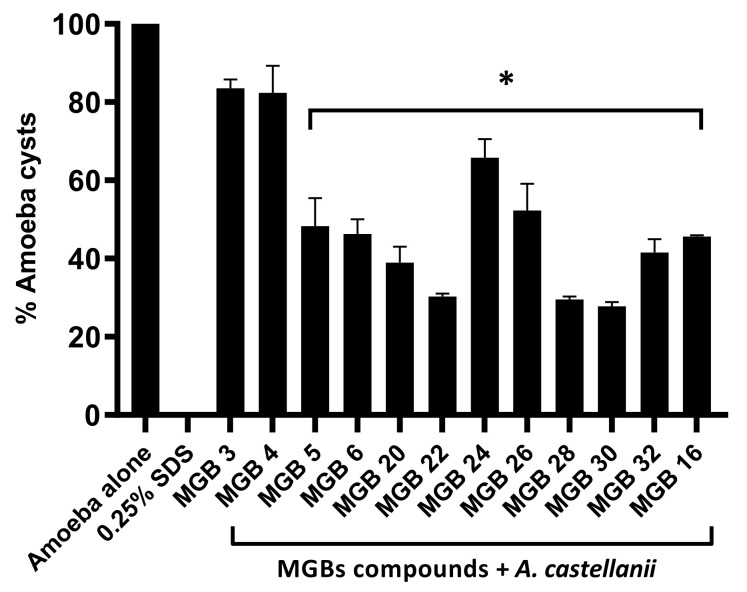
MGB6, MGB20, MGB22, MGB28, MGB30, MGB32, and MGB16 significantly inhibited *A. castellanii* encystation. Significant inhibitory effects of cyst development were recorded by the compounds MGB6, MGB20, MGB22, MGB28, MGB30, MGB32, and MGB16. Data are illustrative of independent experiments pursued and are presented with a mean ± standard error. In addition, *p*-values were determined through the conduction of a two-sample *t*-test, two-tailed distribution; * *p* ≤ 0.05.

The effects of the compounds on *Acanthamoeba* encystation were studied through the conduction of encystation assays. According to the results obtained, MGB6, MGB20, MGB22, MGB28, MGB30, MGB32, and MGB16 were found to significantly inhibit *Acanthamoeba* encystation. In the negative control, 100% cysts were present, indicating that all *Acanthamoeba* trophozoites had transformed into cysts; however, after treatment of amoeba with the compounds MGB6, MGB20, MGB22, MGB28, MGB30, MGB32, and MGB16, *Acanthamoeba* cysts were significantly inhibited, with the presence of only 46.3%, 39%, 30.3%, 29.6%, 27.8%, 41.5%, and 45.6% cysts present (Figure 2). Additionally, the compounds MGB3, MGB4, MGB5, MGB24, and MGB26 did not significantly inhibit *A. castellanii* encystation: 83.5%, 82.3%, 48.3%, 65.8%, and 52.3% cysts were present (Figure 2).

#### 3.2.3. MGB Compounds Were Assessed for Their Effects on *A. castellanii* Excystation

**Figure 3:**
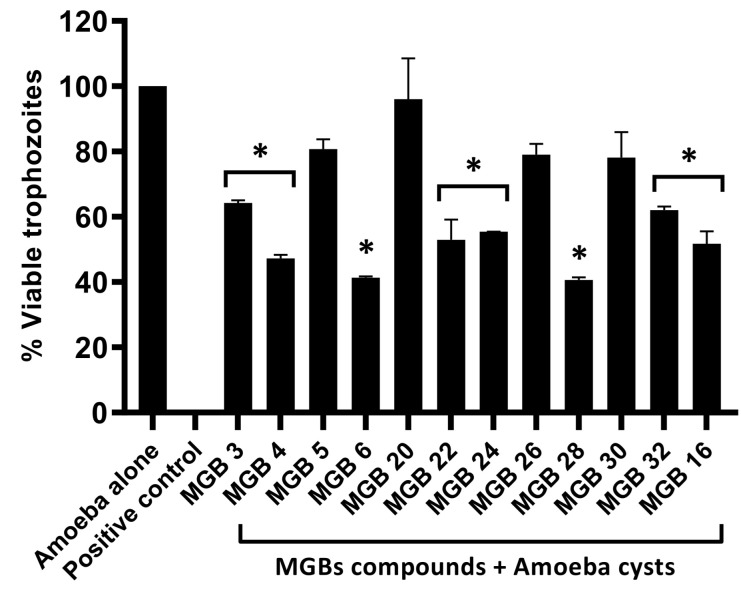
MGB3, MGB4, MGB6, MGB22, MGB24, MGB28, MGB32, and MGB16 significantly inhibited *A. castellanii* excystation. Significant inhibitory effects of cyst re-emergence to trophozoites were recorded for the compounds MGB3, MGB4, MGB6, MGB22, MGB24, MGB28, MGB32, and MGB16. Data are illustrative of independent experiments pursued and are presented with a mean ± standard error. In addition, *p*-values were determined through the conduction of a two-sample *t*-test, two-tailed distribution; * *p* ≤ 0.05.

To study the impact of compounds on *Acanthamoeba castellanii* cyst re-emergence, excystation assays were conducted. The compounds MGB3, MGB4, MGB6, MGB22, MGB24, MGB28, MGB32, and MGB16 were found to significantly inhibit *Acanthamoeba* cyst re-emergence to viable trophozoites (Figure 3). According to the data presented, amoeba cysts in PYG serving as the negative control were able to completely re-emerge as viable trophozoites; hence, 100% viable trophozoites were found in the negative control, whereas the compounds MGB3, MGB4, MGB6, MGB22, MGB24, MGB28, MGB32, and MGB16 significantly reduced the re-emergence of cysts to trophozoites, as the number of viable trophozoites was reduced to 64.3%, 47.3%, 41.4%, 52.9%, 55.4%, 40.6%, 62.1%, and 51.7%, respectively (Figure 3). The remaining compounds, MGB5, MGB20, MGB26, and MGB30, reduced the re-emergence of cysts to trophozoites from 100% to 80.8%, 96.0%, 79.1%, and 78.1%; however, these reductions were not found to be significant.

#### 3.2.4. Limited Cytotoxic Activity Was Observed against Human Cells

**Figure 4:**
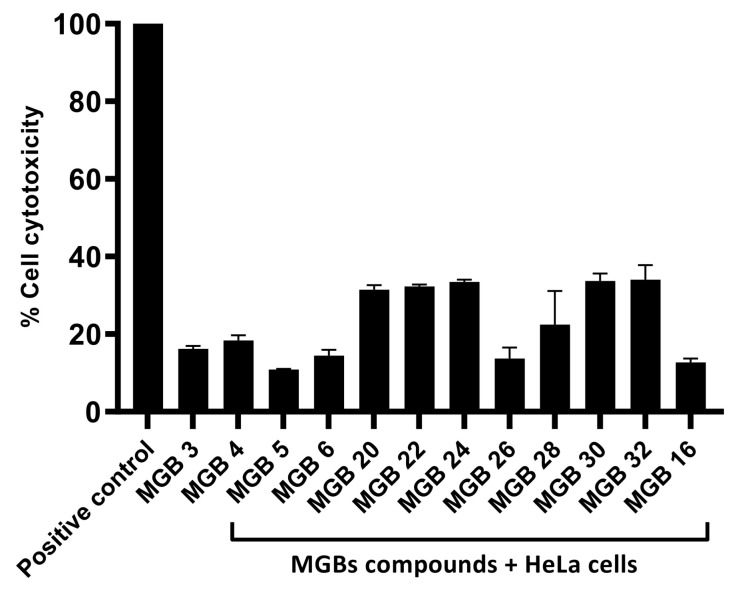
Limited cytotoxic activity was observed against human cells. Negligible and limited cytotoxic activity was found upon treatment of HeLa cell monolayers with 50 µM of the compounds.

Lactate dehydrogenase (LDH) assays were conducted to determine the cytotoxicity of the compounds against human HeLa cells. The compounds were found to exhibit negligible and limited cytotoxic properties at a concentration of 50 µM. Within the positive control, 100% toxic activity was presented due to treatment of cells with Triton X-100 (Figure 4). In comparison, cells treated with MGB3, MGB4, MGB5, MGB6, MGB28, and MGB16 exhibited negligible cytotoxicity, as per the International Organization for Standardization (ISO) 10993-5; the compounds were found to exhibit 16.2%, 18.4%, 10.9%, 14.4%, 13.7%, and 12.7%, respectively (Figure 4) (ISO, 2009; Lopez-Garcia, et al., 2014). Moreover, the compounds MGB20, MGB22, MGB24, MGB26, MGB30, and MGB32 exhibited 31.4%, 33.7%, 32.3%, 33.5%, 34.0%, and 22.4%, respectively (Figure 4).

#### 3.2.5. MGB Compound Effects on Amoeba-Mediated Cytotoxicity against Human Cells Were Determined

*Acanthamoeba*-mediated host-cell death was determined through the conduction the of cytopathogenicity assays. *Acanthamoeba* was pretreated with the various compounds and incubated for 2 h. Next the pretreated amoeba was introduced to the human cells, and following a 24 h incubation period, the amoeba-mediated cell cytotoxicity was determined.

**Figure 5:**
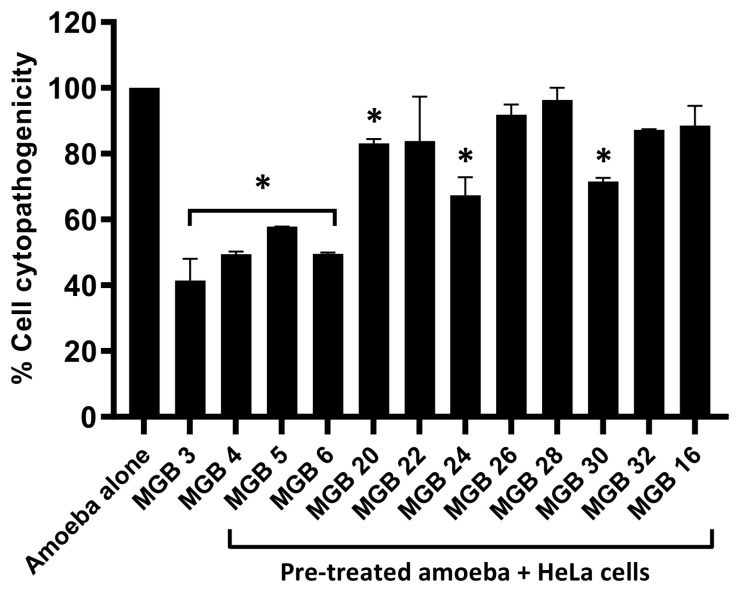
Compounds exhibited decrease in amoeba-mediated cytotoxicity against human cells. The compounds exhibited a decrease in amoeba-mediated host-cell death at varying degrees upon the treatment of 5 × 10^5^ amoebae with 50 µM of the compound for 2 h. The amoebae were introduced to HeLa cell monolayers overnight, and the host-cell death was measured; * *p* ≤ 0.05.

According to the results, the compounds were found to reduce the host-cell death; however, certain compounds exhibited greater reduction compared to others. For example, the compounds MGB5, MGB20, MGB22, MGB24, MGB26, MGB28, MGB30, MGB32, and MGB16 were found to reduce amoeba-mediated host-cell death between the range of 3.7% and 42.2%, upon comparison with the negative control (Figure 5).

MGB5, MGB20, MGB22, MGB24, MGB26, MGB28, MGB30, MGB32, and MGB16 presented 57.8%, 83.2%, 71.5%, 83.8%, 67.3%, 91.9%, 87.3%, 96.3%, and 88.6% cytotoxic activity due to amoeba, respectively, while the negative control (amoeba alone with cells) exhibited 100% amoebicidal activity (Figure 5). Furthermore, the compounds MGB3, MGB4, and MGB6 exhibited the greatest reduction in amoeba-mediated host-cell death, with cell death reduced to 41.5%, 49.4%, and 49.5%, respectively, while untreated amoeba-mediated host-cell death (negative control) was 100% (Figure 5).

## 4. Discussion

### 4.1. MGBs Activity against Acanthamoeba castellanii

*Acanthamoeba castellanii* is a free-living protozoon that is found globally in the environment, such as in soil, water, atmosphere, and dust [2]. This free-living amoeba exists in two stages, namely a trophozoite stage and a cyst stage, and is responsible for the fatal central nervous infection granulomatous amoebic encephalitis and the sight-threatening infection *Acanthamoeba keratitis* [2,4]. Individuals who depend on water-tanks, and those participating in water-related activities such as swimming in lakes and swimming pools, as well as contact-lens wearers, are more prone to encountering this amoeba [4]. Additionally, the amoeba is a threat to public health by acting as the Trojan horse of the microbial world; *Acanthamoeba* is capable of hosting a variety of pathogens within it and acting as a reservoir for them [21,22]. However, no efficient treatment is currently present. Current treatment options involve a combination drug therapy of diamidines, biguanides, and antifungals to kill the amoeba trophozoites [2]. The amoeba cysts are harder to kill, as they are capable of withstanding the harsh conditions of treatments [2]. It is of note that cysts are incapable of infecting an individual, and, hence, drugs that can lead to the encystment of amoeba and prevent their re-emergence can provide new medication options. Of course, the establishment of a drug that is effective against both amoeba trophozoites and cysts is much greater.

In this study, the compounds were tested for their antiamoebic properties against *Acanthamoeba castellanii*. A series of amoebicidal, encystation, excystation, cytopathogenicity, and cytotoxicity assays were carried out. The compounds MGB6, MGB16, and MGB22 were found to exhibit significant amoebicidal activity and inhibit the process of encystation and excystation of *A. castellanii*. These three compounds were the only compounds proving to be significant in all three assays. Moreover, all compounds reduced amoeba-mediated host-cell death, with the highest reduction being that of MGB3 at 41.5%. Additionally, the compounds were found to possess negligible and limited cytotoxic activity against human cells; the compound MGB30 exhibited the highest cytotoxic activity, at 34%. Moreover, the MIC_50_ values of the compounds MGB3, MGB6, and MGB20 were determined to be 65.91, 58.34, and 83.62 µM, respectively. The results further revealed that MGB compounds showed MIC_50_ at micromolar concentrations that highlight their importance as potent anti-parasitic drugs against *A. castellanii*.

Various studies by different researchers exploring different compounds are being conducted against *A. castellanii*, with the aim to develop a more efficient treatment strategy against *A. castellanii* infections. For example, a group of researchers have studied the effects of povidone-iodine against *Acanthamoeba* cysts and trophozoites, and they found that a 0.5 to 2.5% solution exhibits a good amount of antiamoebic activity [23]. Another group of researchers studied the effects of dihydropyridines synthesized by cyclic dimerization of alkylidene malononitrile derivatives for their antiamoebic activity; their results concluded the efficacy of the compounds against *A. castellanii* cysts and trophozoites [24].

Further in vivo studies to understand the effects of the compounds in animal models such as mice are necessary. Further research regarding the use of the compounds as disinfectants can also be carried out.

### 4.2. The Rationale of MGBs Design

The discovery of the minor groove binder, distamycin, paved the way for the design and synthesis of sequence-specific DNA minor groove binders. These efforts have been led by Lown and co-workers, who developed crosslinked polyamide dimers [25], and Dervan’s group, who developed hairpin-linked [26] and cyclic polyamide dimers [27] to target specific DNA sequences. However, due to the high polarity and large molecular weight of these molecules, their cell permeability was adversely affected. The absorption studies carried out on these MGBs showed low Caco-2 permeability, suggesting that these molecules may not be orally bioavailable [27]. Other research groups attempted to incorporate alkylating functional groups (e.g., nitrogen mustard and bromoamide) to the *N*-terminus of the MGB structure to enhance the specificity and potency of the drug. Two examples on this strategy are brostallicin [28] and tallimustine [29], which act by targeting the minor groove and alkylating the DNA bases, causing irreversible DNA damage. Due to the toxicities associated with alkylation, this strategy was not successful in developing MGBs as commercial drugs. Suckling and co-workers prepared a series of distamycin derivatives as antibacterial, antifungal, and antiparasitic agents [30,31]. Suckling’s approach aimed to enhance the lipophilcity of these compounds by introducing lipophilic moieties in the “head” substructure, adding alkyl groups at the heterocyclic rings and modifying the “tail” substructure. This approach has proved to be very successful in developing a new antibacterial drug, MGB-BP-3, which has recently completed Phase IIa clinical trial for the treatment of *C. difficile* infections [31]. In the current study, we followed Suckling’s approach to design novel MGBs with improved physical properties by enhancing their lipophilicity via the introduction of new lipophilic moieties at the “head” position, such as 4-stilbenecarboxylic acid and 4-(phenylethynyl)benzoic acid, which had not been incorporated into the MGB structures previously. Moreover, thiazole ring was used to replace *N*-methylpyrrole/or imidazole at different parts of the MGB molecule (at the “head” or close to the “tail” group, e.g., MGB4), not only to enhance the lipophilicity, but also to allow these MGBs to target GC-rich DNA sequences, contrary to the MGBs prepared by Suckling’s group that target AT-rich sites [31]. This helps to expand the library of MGB structures and provides structurally diverse derivatives with different properties that could be exploited in the development of MGBs as therapeutic drugs. Furthermore, the 3-carbon spacer between the amide link and morpholine ring in the tail group hugely affected the basicity of all MGBs with *N*-(3-aminopropyl)morpholine tail in our study. The measured pKa of these MGBs is 7.01 (Table 1), which makes them soluble in water at physiological pH (~7), a characteristic that is essential for systemic oral drugs. However, the MGBs prepared by Suckling’s group with 2-carbon spacer between the amide link and morpholine ring, e.g., MGB-BP-3, has a pKa of 5.26 [31], making it insoluble in water at a pH of 7, thus indicating that such MGBs might be used as non-systemic oral drugs. The *N*-(3-aminopropyl)morpholine tail does not only affect the pKa and the solubility of MGBs in aqueous solutions, but also enhances their lipophilicity. All MGBs with morpholine tail showed higher logD and logP values compared with the counterpart MGBs with dimethylaminopropyl tail (Table 1).

The prepared MGBs are expected to bind reversibly in the minor groove via non-covalent bonds. Such interactions might alter the DNA structure and lead to the inhibition of DNA gyrases and topoisomerases [32,33]. However, the varied activity against *Acanthamoeba castellanii* that was noticed among the MGBs can be attributed to the ability of these molecules to target different DNA sequences with different affinities. MGBs with imidazole/thiazole rings prefer to target GC-rich sites, while MGBs with *N*-methylpyrrole and other aromatic rings such as benzene, pyridine, furane, thiophene, and quinoline bind to AT-rich DNA sites [34]. Furthermore, the binding affinity of different MGBs with DNA is not expected to be equivalent; a slight variation in the structure might massively alter the binding affinity, and that will be reflected on the ligand’s bioactivity. Another crucial factor that affects the activity is the ligand’s physical properties and its ability to cross biological membranes. Some polar MGBs can interact with DNA; however, the inability to cross lipophilic membranes renders them inactive. For instance, all MGBs that showed activity against *Acanthamoeba castellanii* have logP values higher than 1, while MGB5 (with imidazole ring at the head position), which has a measured logP of 0.56, showed no activity, although its structure is similar to the active compound MGB6 (with thiazole ring at the head position), and our biophysical analysis also showed the ability of MGB5 to bind with DNA. These findings indicate that the lipophilicity of MGBs plays a crucial role in their biological activity.

## Figures and Tables

**Table 1:** Synthesis of the final MGB products. The pKa’s, Log P’s, and Log D’s of MGBs were determined by using the Marvin application (https://www.chemaxon.com (accessed on 15 May 2022)).

ID	Reactants		Reagents	Product Structure	Log D pH 7.4	Log P	pka
**MGB3**	**9**	**5**	1. Pd/C, H_2_ 2. HBTU	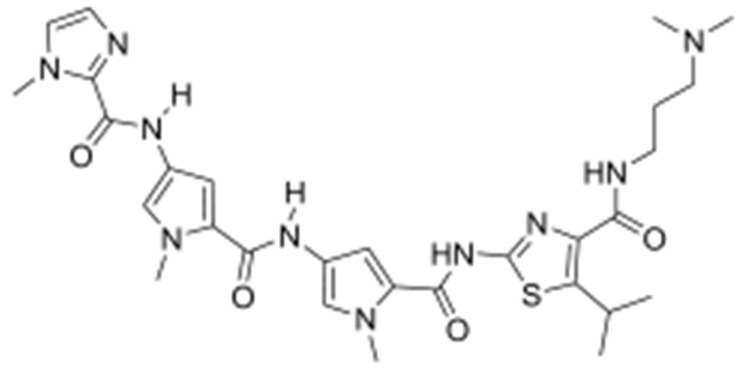	0.55	2.44	9.3
**MGB4**	**10**	**5**	1. Pd/C, H_2_ 2. HBTU	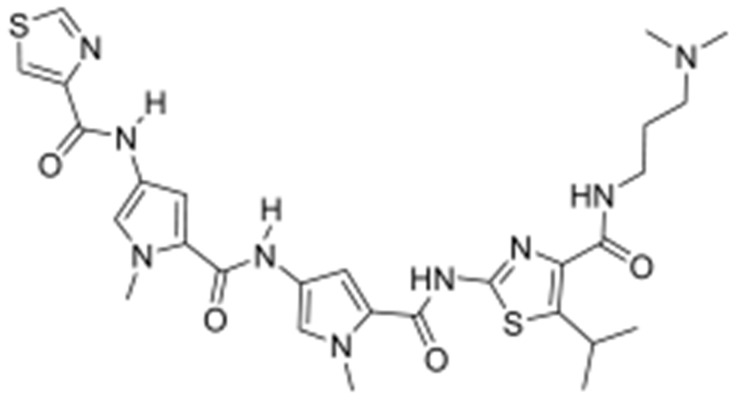	1.11	3.00	9.3
**MGB5**	**9**	**7**	1. Pd/C, H_2_ 2. HBTU	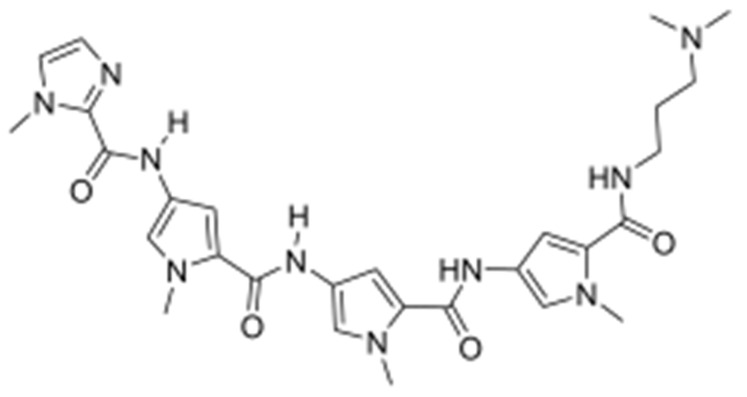	−1.34	0.56	9.30
**MGB6**	**10**	**7**	1. Pd/C, H_2_ 2. HBTU	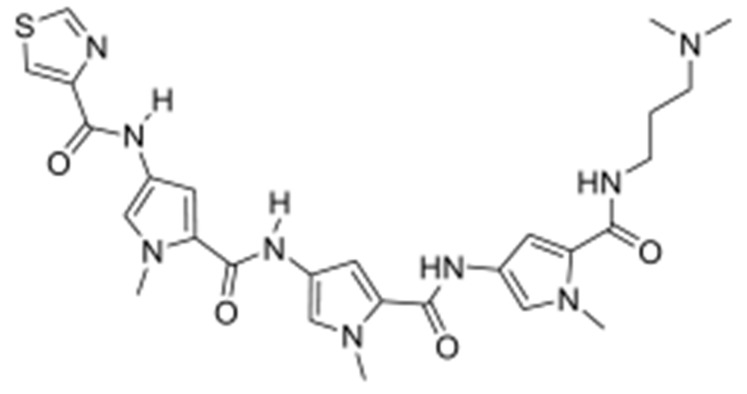	−0.78	1.12	9.30
**MGB16**	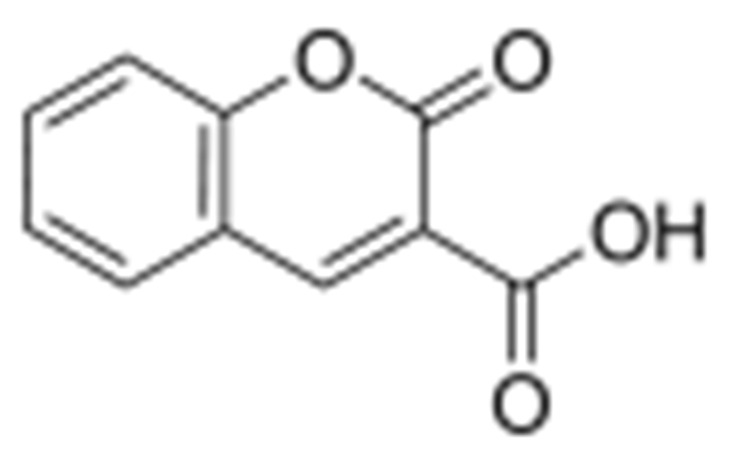	**8**	1. Pd/C, H_2_ 2. HBTU	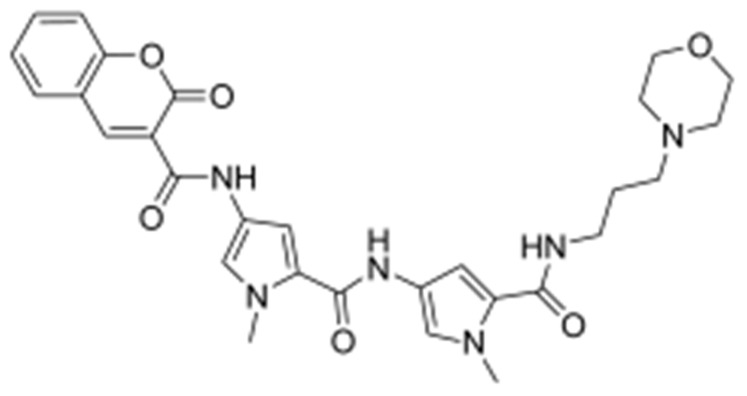	1.55	1.69	7.01
**MGB20**	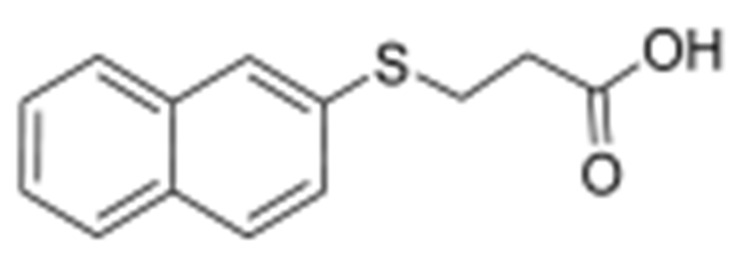	**8**	1. Pd/C, H_2_ 2. HBTU	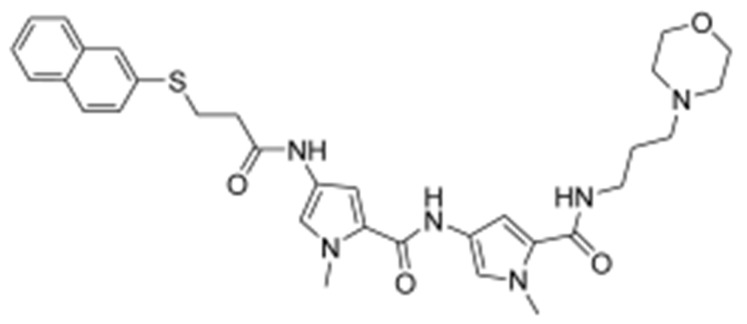	2.96	3.11	7.01
**MGB22**	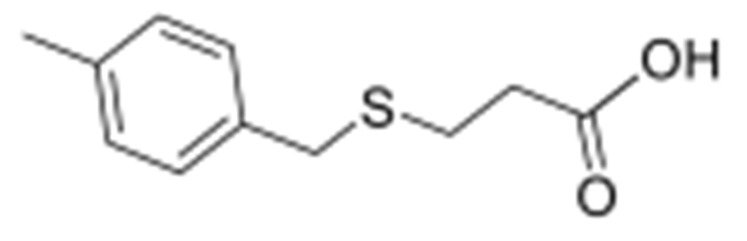	**7**	1. Pd/C, H_2_ 2. HBTU	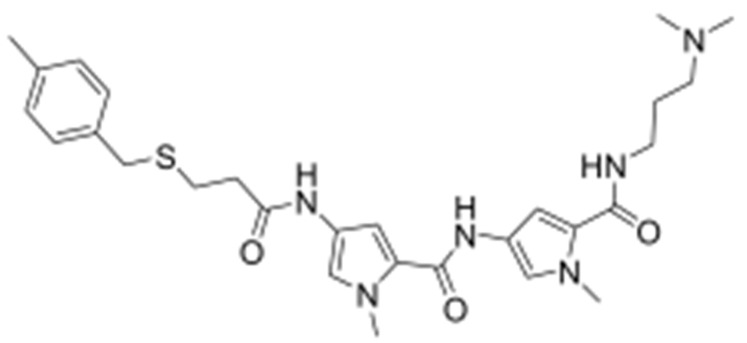	1.2	3.10	9.30
**MGB24**	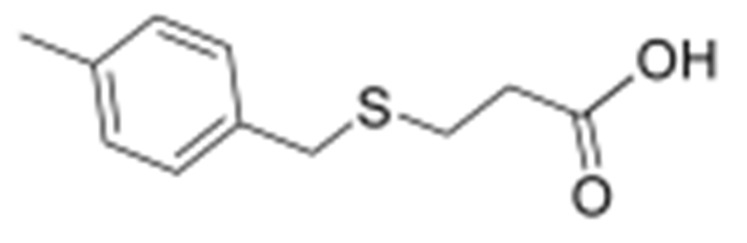	**8**	1. Pd/C, H_2_ 2. HBTU	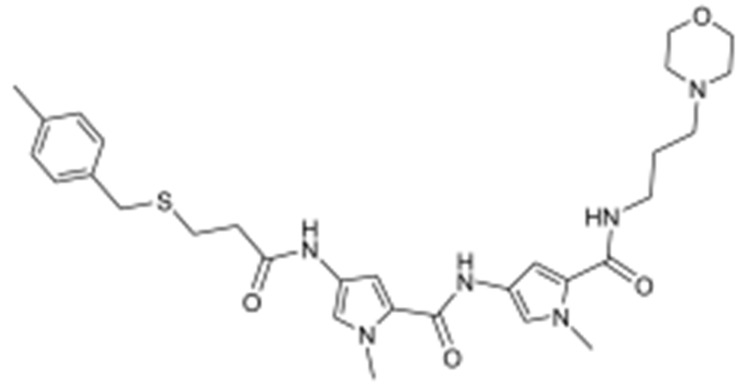	2.73	2.88	7.01
**MGB26**	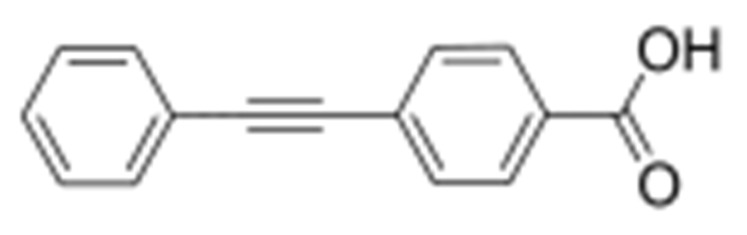	**7**	1. Pd/C, H_2_ 2. HBTU	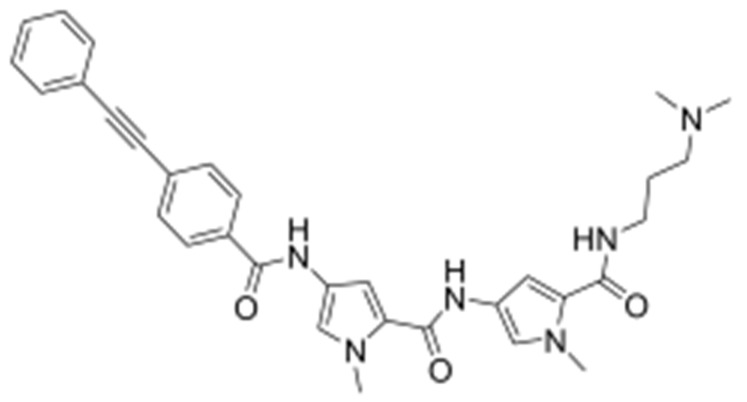	1.99	3.88	9.30
**MGB28**	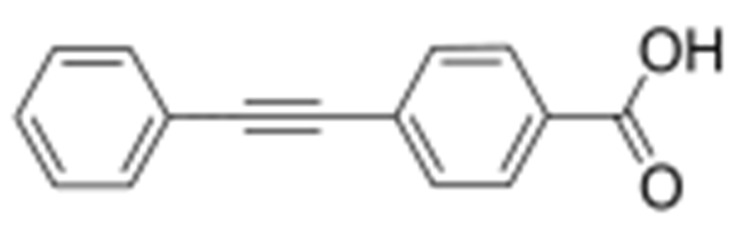	**8**	1. Pd/C, H_2_ 2. HBTU	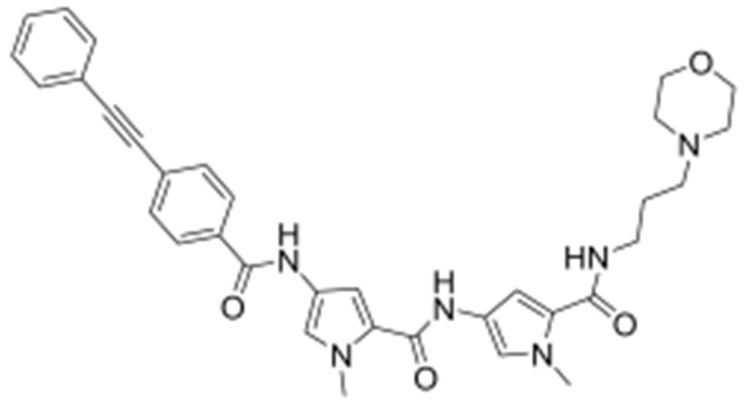	3.51	3.66	7.01
**MGB30**	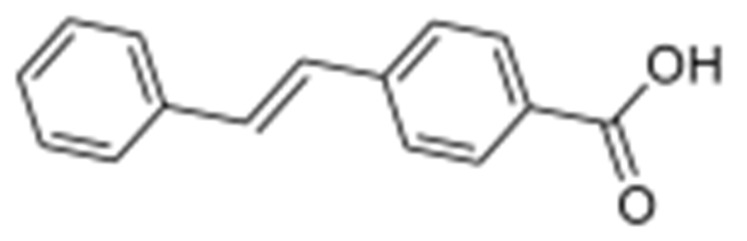	**7**	1. Pd/C, H_2_ 2. HBTU	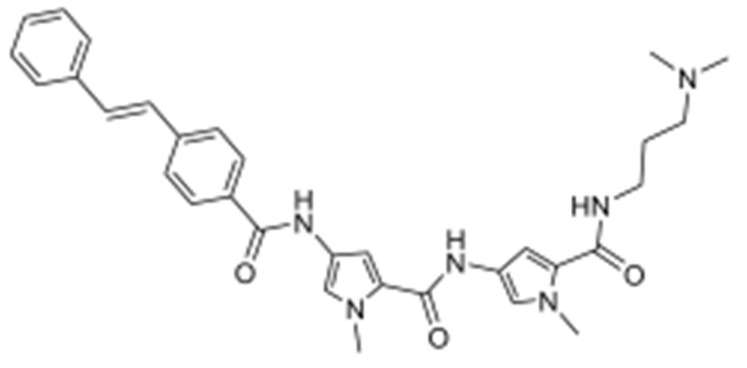	2.2	4.1	9.3
**MGB32**	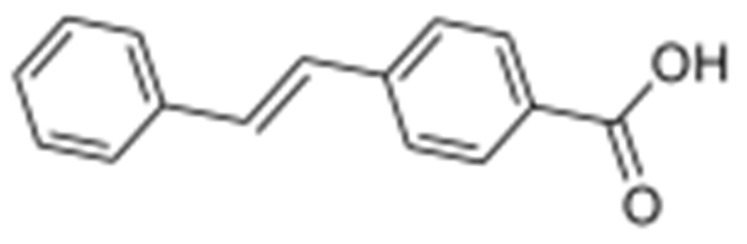	**8**	1. Pd/C, H_2_ 2. HBTU	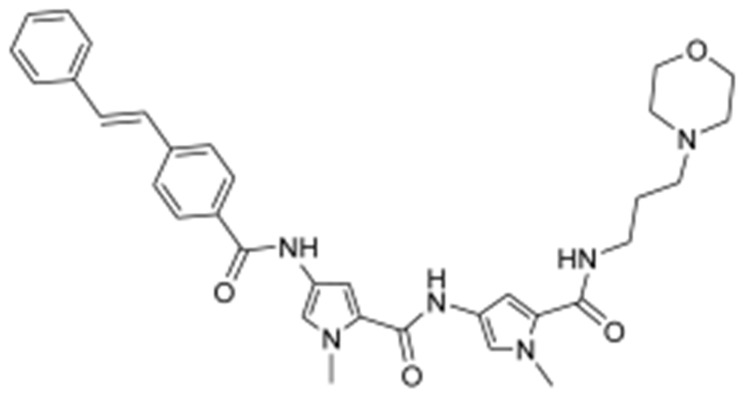	3.73	3.88	7.01

**Table 2:** The MIC_50_ of the MGBs compounds against *A. castellanii*.

*A. castellanii*					
	25 µM	50 µM	75 µM	100 µM	MIC_50_
**Amoeba Alone**	100				
**MGB3**	43.7 ± 3.8	64 ± 6.7	51 ± 4.7	46 ± 4.8	65.91
**MGB6**	57 ± 2.4	51 ± 1.1	48 ± 5.5	45 ± 1.4	58.34
**MGB20**	64 ± 3.2	58 ± 2.0	54.6 ± 5.8	48 ± 1.1	83.62

## Data Availability

Not applicable.

## Supplementary Materials

The following are available online at https://www.mdpi.com/article/10.3390/antibiotics11070935/s1, NMR and mass spectra of the prepared compounds.

## Abbreviations

HBTU, 2-(1H-benzotriazol-1-yl)-1,1,3,3-tetramethyluronium hexafluorophosphate, Hexafluorophosphate Benzotriazole Tetramethyl Uronium; MGB, minor groove binder; DMF, dimethylformamide; TEA, trimethylamine; THF, tetrahydrofuran; DMSO, dimethylsulfoxide; DCM, dichloromethane; MeOH, methanol; PD/C, palladium on carbon; LogP, partition coefficient; LogD, partition coefficient at specific pH; p*ka*, logarithm acid dissociation constant.
