# Supplementary material for: Synthesis and Evaluation of Novel DNA Minor Groove Binders as Antiamoebic Agents
Source: Antibiotics (Basel). 2022 Jul 13;11(7):935. doi: 10.3390/antibiotics11070935 (PMC9312114; doi:10.3390/antibiotics11070935)

Supporting information

Synthesis and evaluation of novel DNA minor groove binders as antiamoebic agents

Hasan Y. Alniss[1,2]*, Naveed A. Khan[3]*, Anania Boghossian[4], Noor Akbar[4], Hadeel M. Al-Jubeh[2], Yousef A. Msallam[1,2], Balsam Q. Saeed[3], Ruqaiyyah Siddiqui[4]

[1] College of Pharmacy, University of Sharjah, Sharjah, P.O. Box 27272, United Arab Emirates Arab Emirates; [2]Sharjah Institute for Medical Research, University of Sharjah, Sharjah, P.O. Box 27272, United Arab Emirates; [3]College of Medicine, Department of Clinical Sciences, University of Sharjah, Sharjah, P.O. Box 27272, United Arab Emirates; [4]College of Arts and Sciences, American University of Sharjah, University City, Sharjah, P.O. Box 26666, United Arab Emirates

Contents: NMR and mass spectra of the prepared compounds

* **Correspondence:** N. A. Khan, Department of Clinical Sciences, College of Medicine, University of Sharjah, Sharjah, United Arab Emirates 27272. Tel: +971-6505-7722. E-mail: nkhan@sharjah.ac.ae or naveed5438@gmail.com

Hasan Y. Alniss, College of Pharmacy, University of Sharjah, P.O. Box 27272, Sharjah, United Arab Emirates Arab Emirate 27272. Tel: +(971)-6-5057427. E-mail: halniss@sharjah.ac.ae

^{1}H NMR (CHLOROFORM-d) δ 1.79 (2H, m, CH_2), 2.53 (4H, s, NCH_2), 2.57 (2H, t, NCH_2), 3.50 (2H, q, CONH--CH_2), 3.76 (4H, t, OCH_2), 4.00 (3H, s, NCH_3), 7.10 (1H, d, Ar—H), 7.54 (1H, d, Ar—H), 7.94 (1H, s, CONH) ; **(2b)**

AB-3-1H.esp

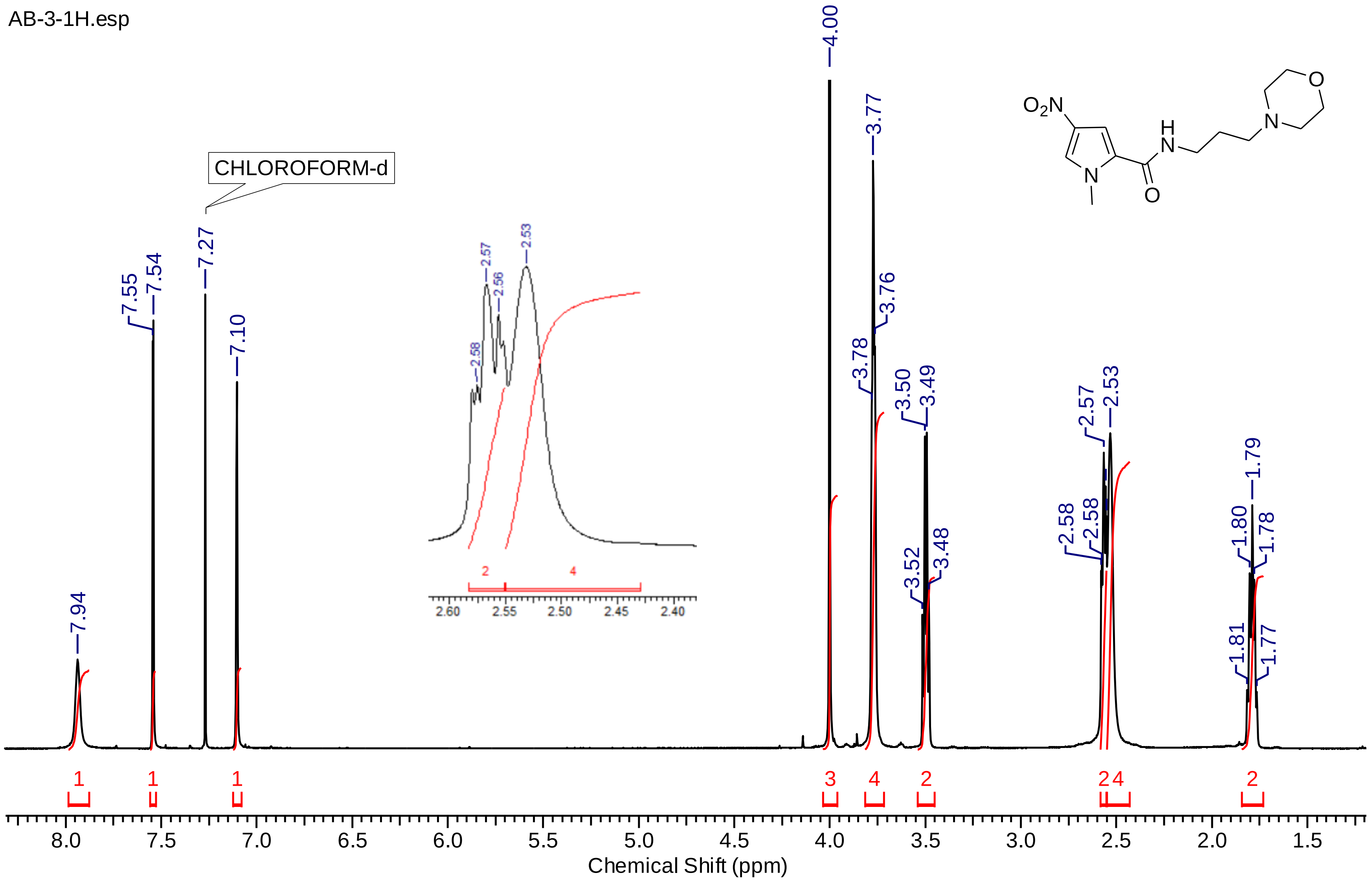

^{13}C NMR (CHLOROFORM-d): δ 23.55, 37.51, 39.77, 53.61, 58.39, 66.58, 106.45, 126.06, 126.49, 134.62, 160.02; **(2b)**

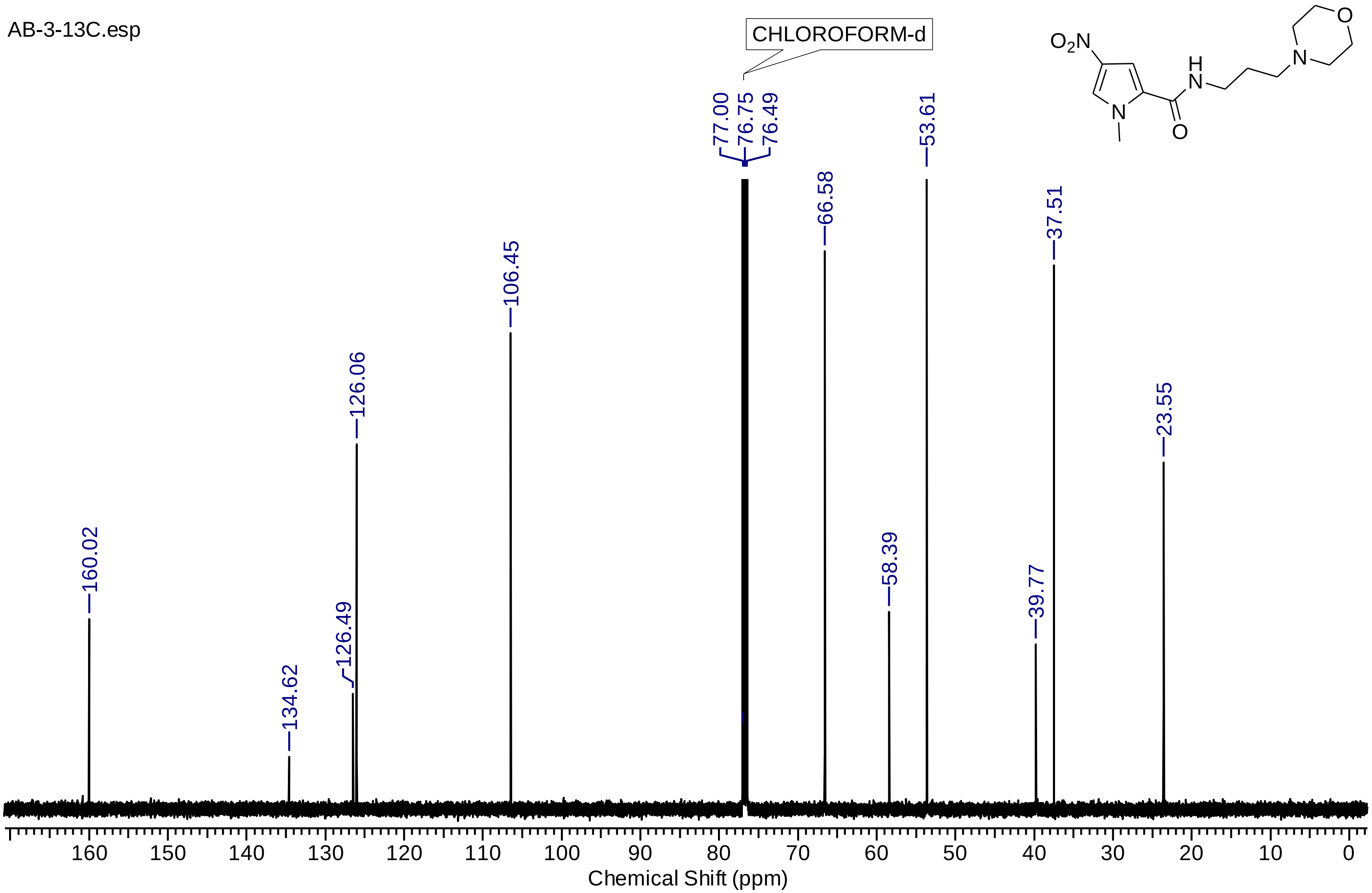

LC−MS (ESI): m/z calcd for $C_{13}H_{20}N_4O_4$, 296.15, found 297.14 [M + H]+. **(2b)**

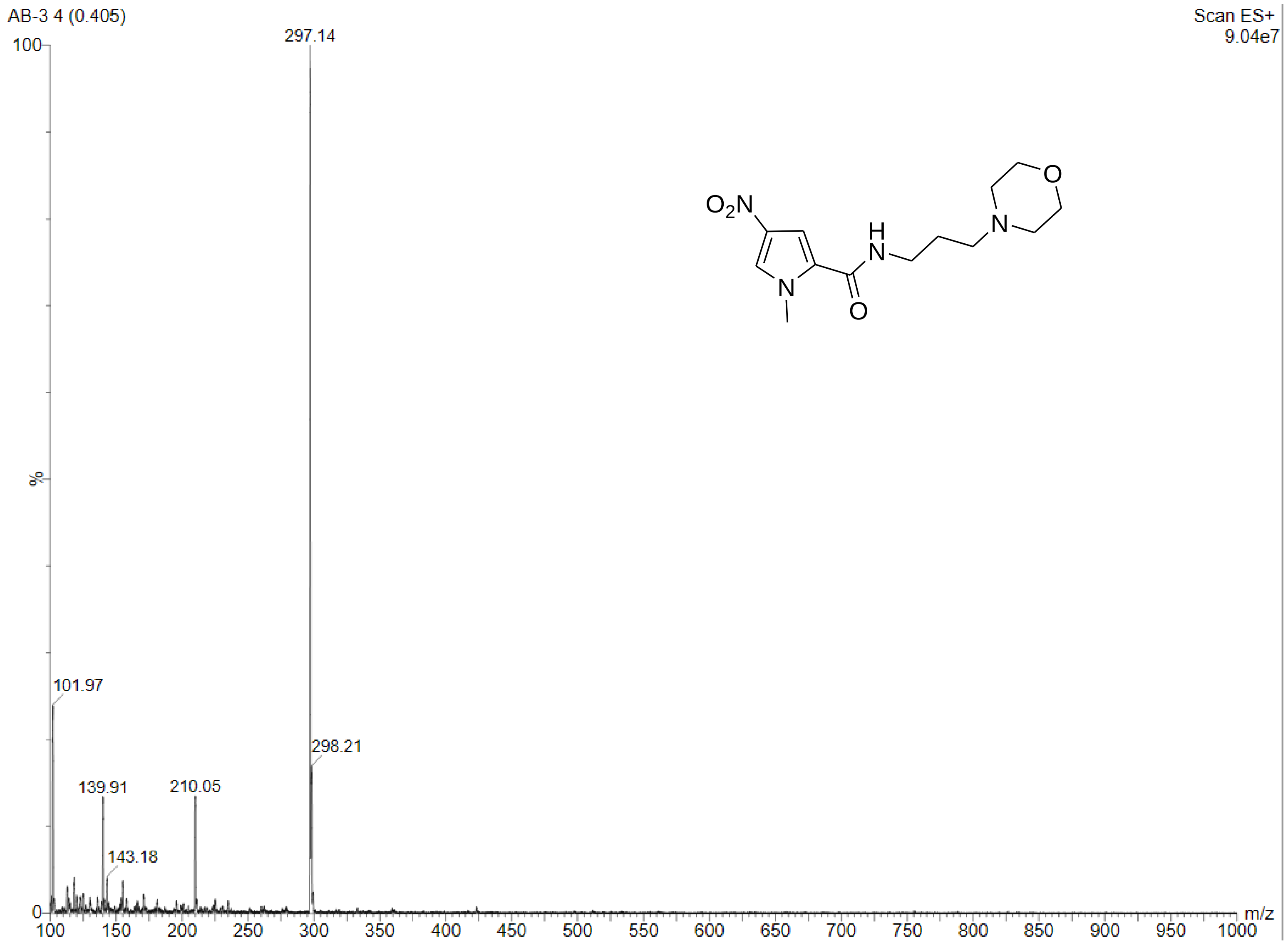

^{1}H NMR (CHLOROFORM-d) δ 1.77 (2H, m, CH_2), 2.37 (6H, s, NCH_3), 2.56 (2H, t, NCH_2), 3.50 (2H, q, CONH--CH_2), 4.01 (3H, s, NCH_3), 6.98 (1H, d, Ar—H), 7.53 (1H, d, Ar—H), 8.67 (1H, s, CONH) ; **(2a)**

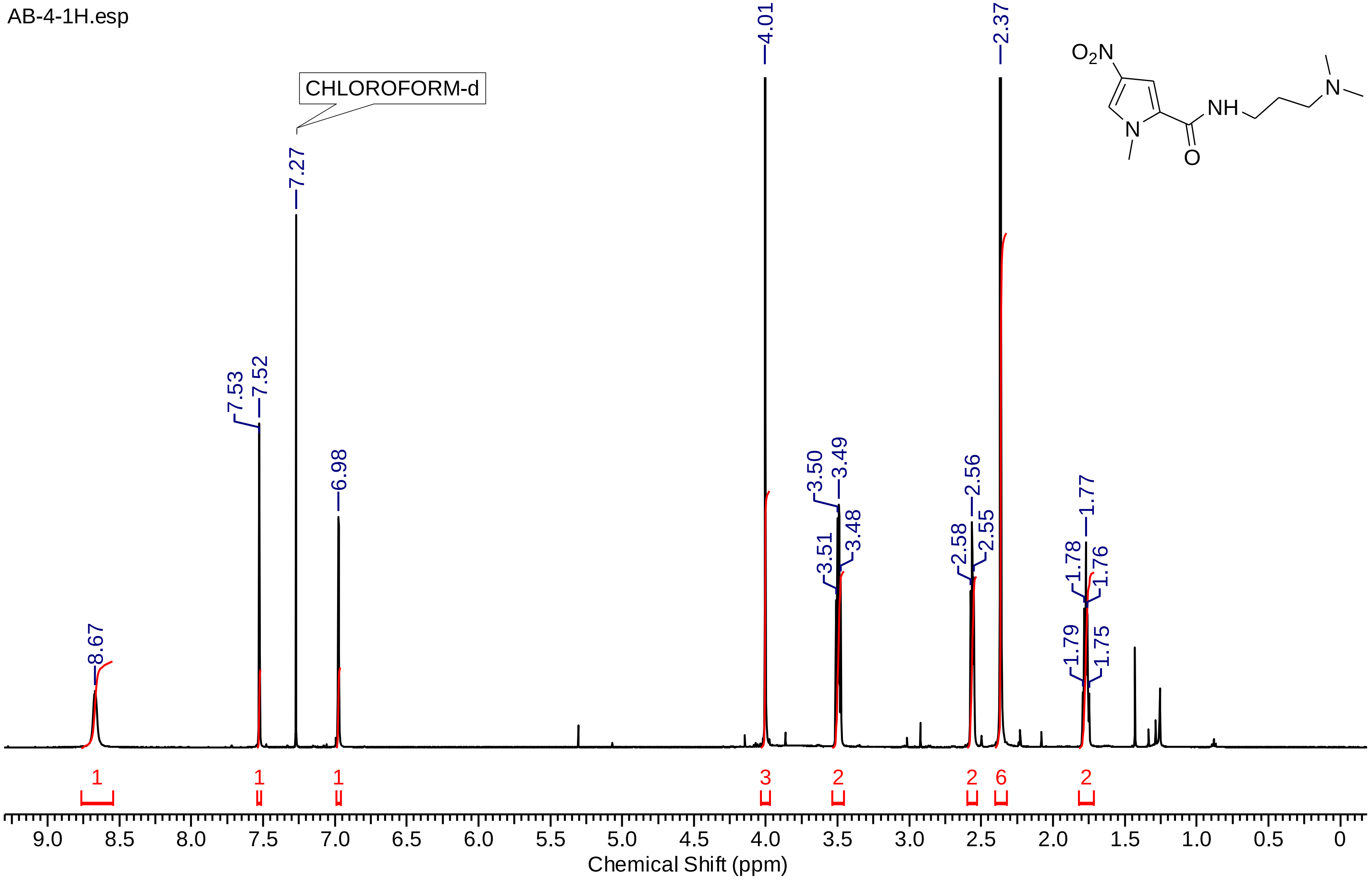

^{13}C NMR (CHLOROFORM-d): δ 24.60, 37.88, 40.19, 45.25, 59.37, 106.51, 126.28, 126.92, 134.91, 160.26; **(2a)**

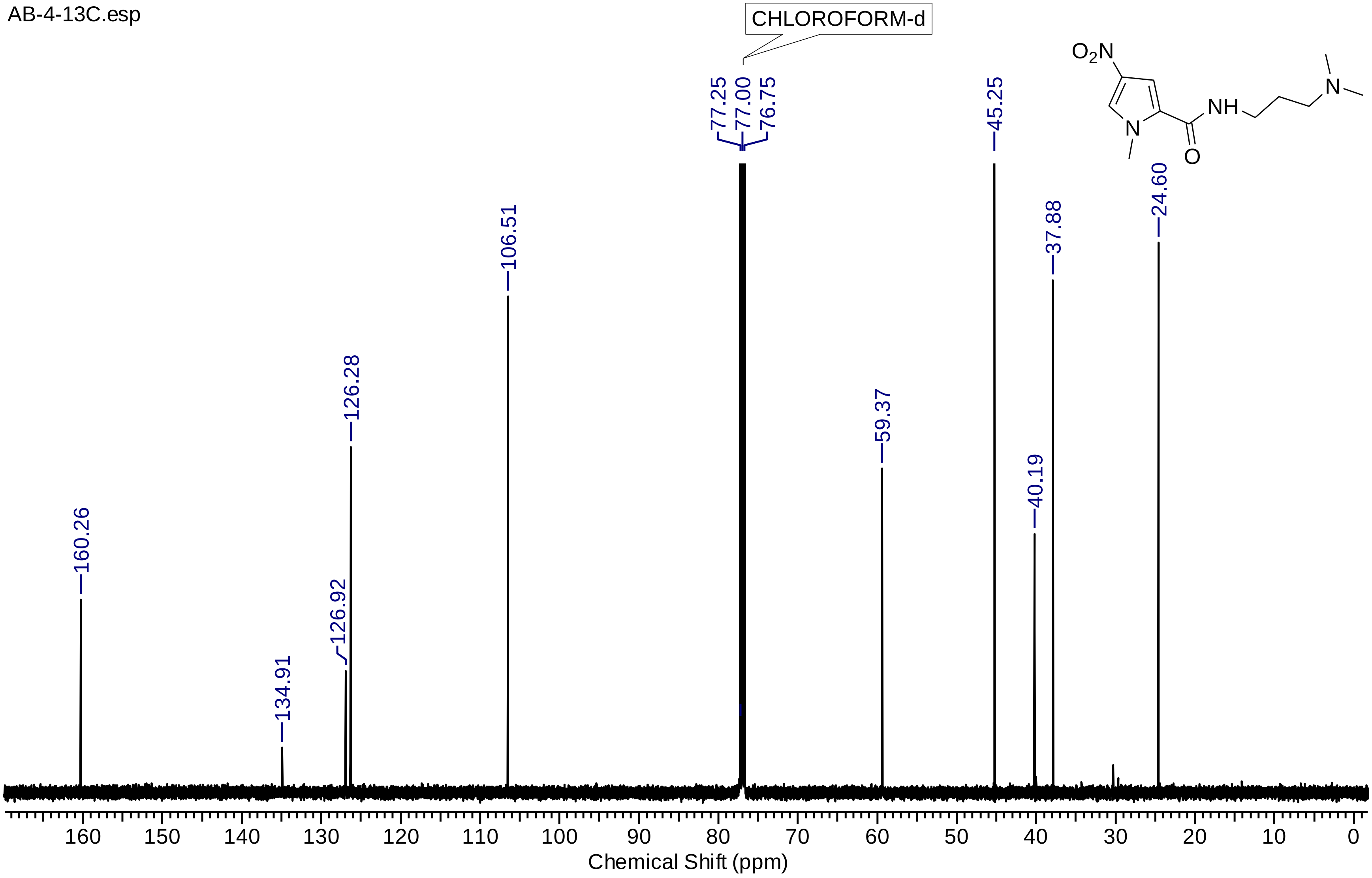

LC−MS (ESI): m/z calcd for $C_{11}H_{18}N_4O_3$, 254.14, found 255.21 [M + H]+. **(2a)**

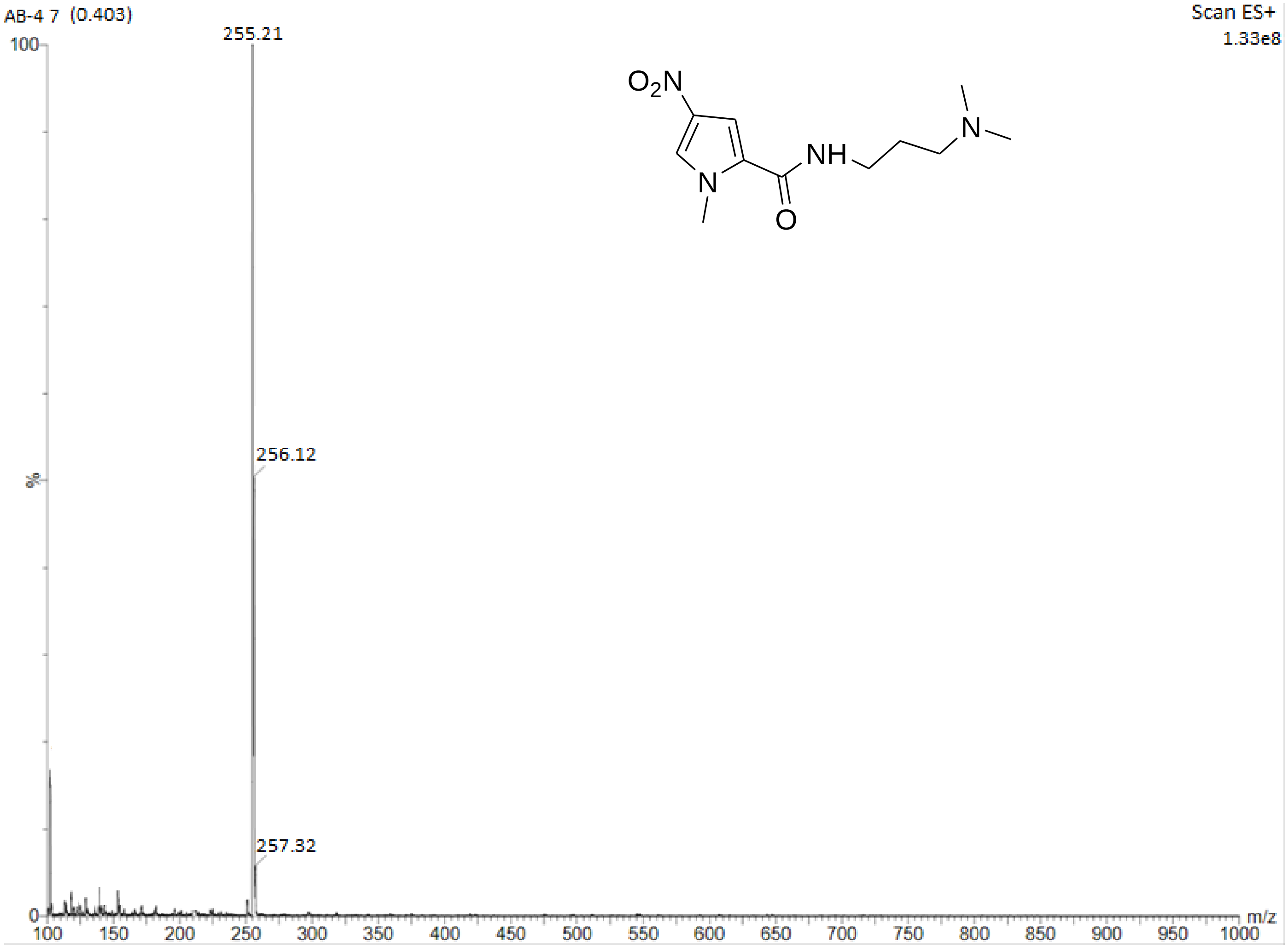

^{1}H NMR (CHLOROFORM-d) δ 3.87 (3H, s, OCH_3), 4.00 (3H, s, NCH_3), 7.42 (1H, d, Ar—H), 7.61 (1H, d, Ar—H) ; **(4b)**

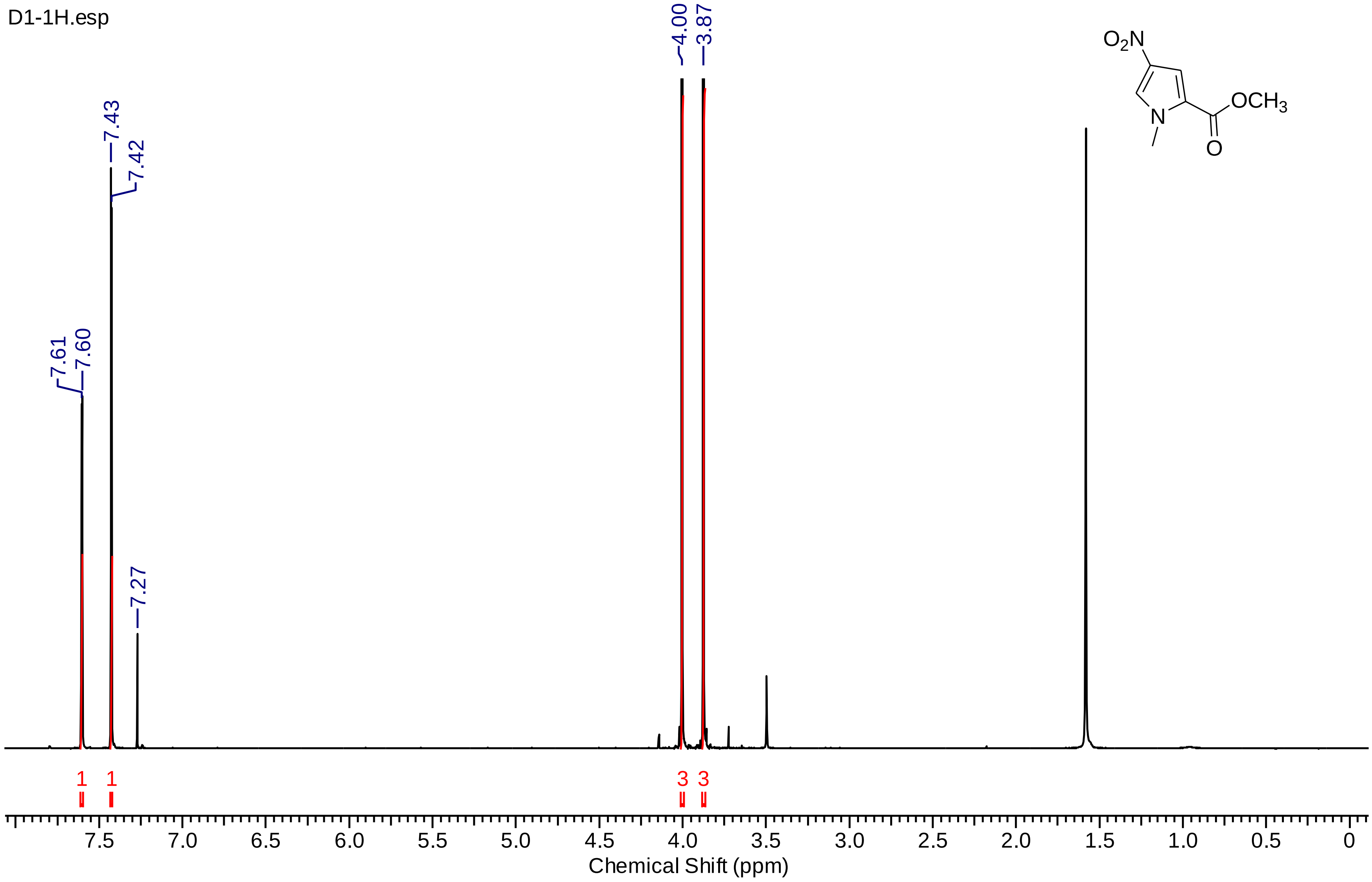

^{13}C NMR (CHLOROFORM-d): δ 37.97, 51.86, 112.78, 122.84, 127.53, 160.62; **(4b)**

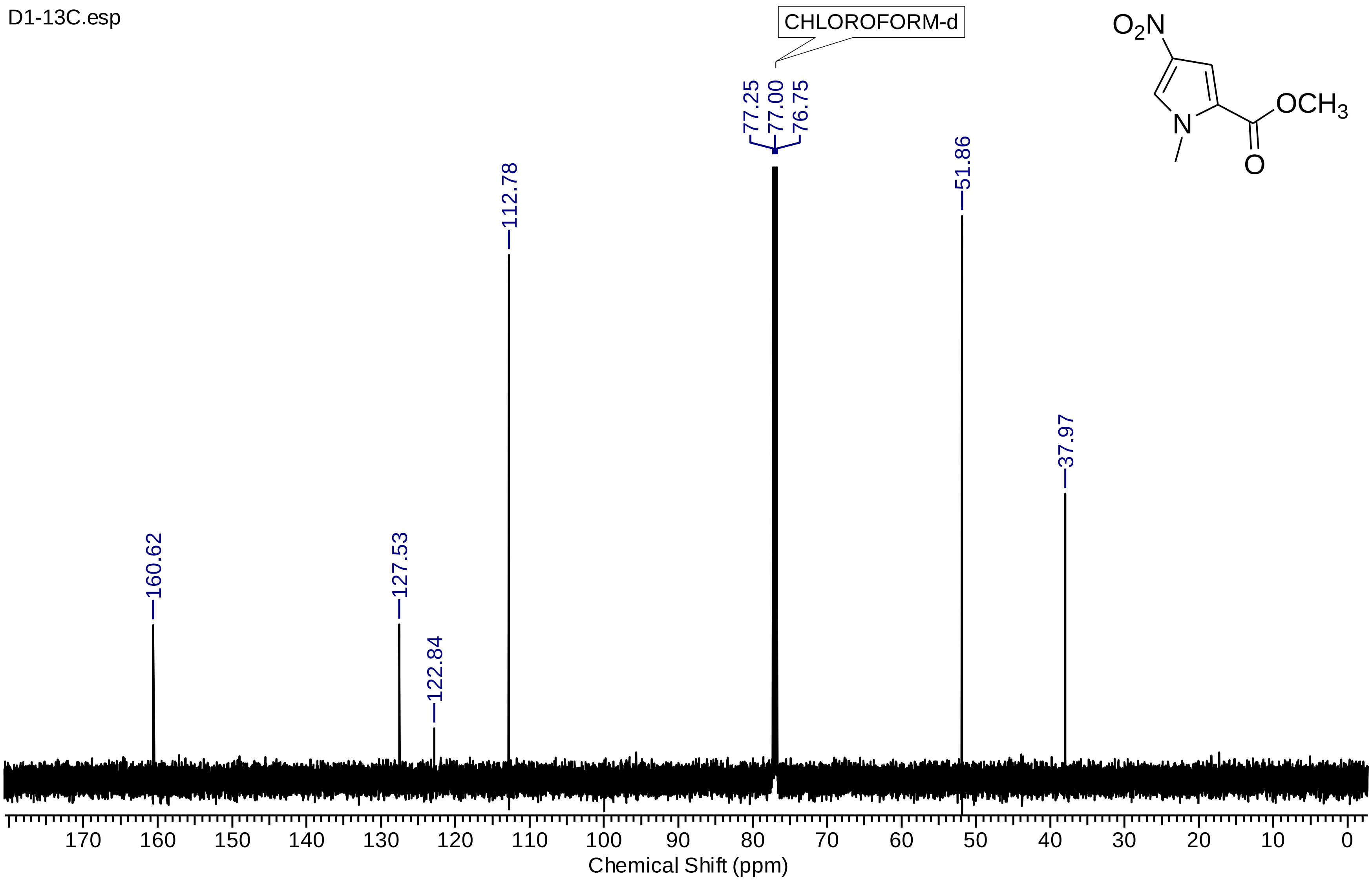

LC–MS (ESI): m/z calcd for $C_7H_8N_2O_4$, 184.85, found 184.89 [M + H]+. **(4b)**

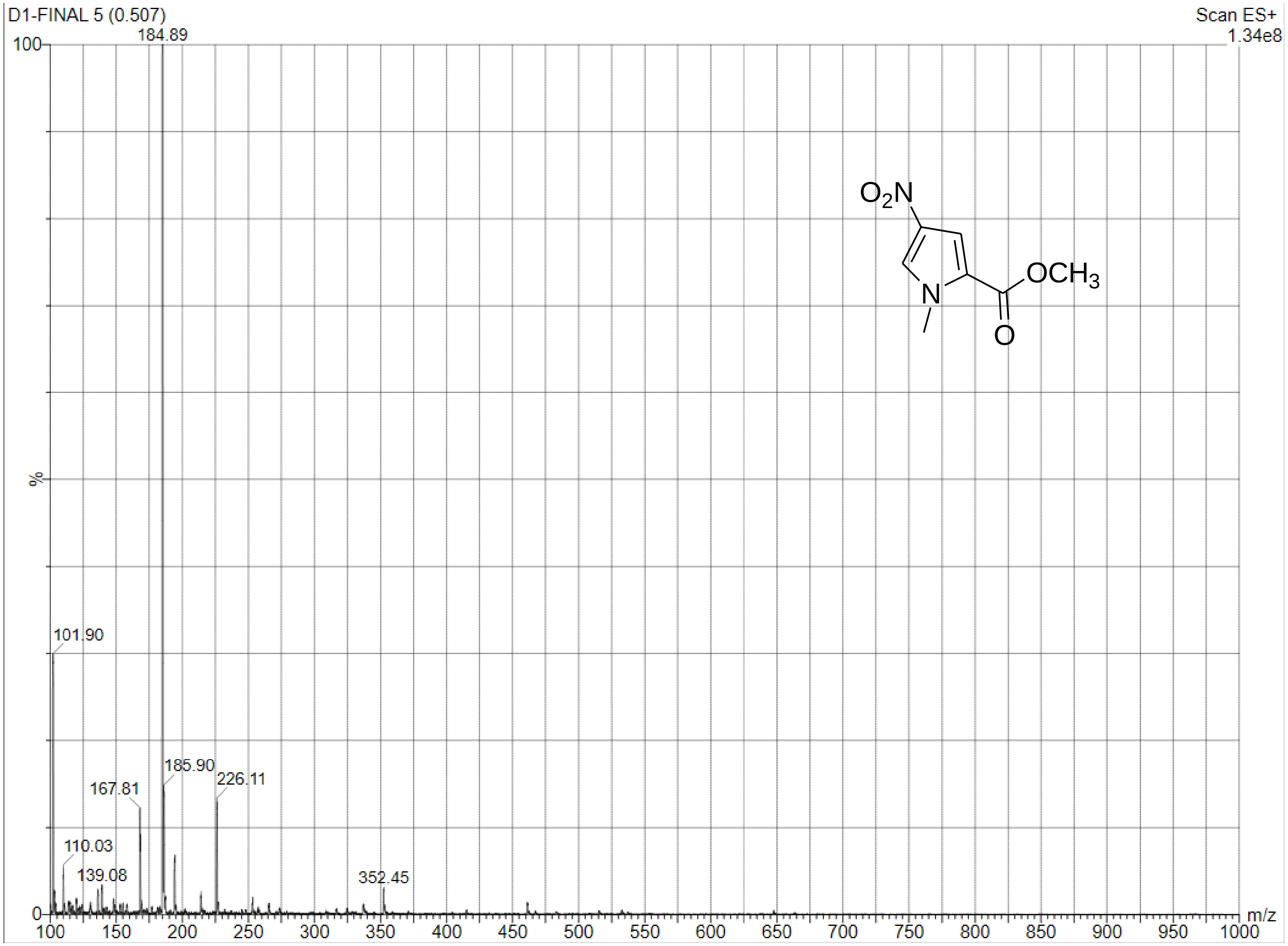

^{1}H NMR (ACETIC ACID-d_4) δ 3.75 (3H, s, OCH_3), 3.90 (3H, s, NCH_3), 3.94 (3H, s, NCH_3), 6.88 (1H, d, Ar—H), 7.45 (1H, d, Ar—H), 7.59 (1H, d, Ar—H), 7.65 (1H, d, Ar—H) ; **(6a)**

DIM-1H.esp

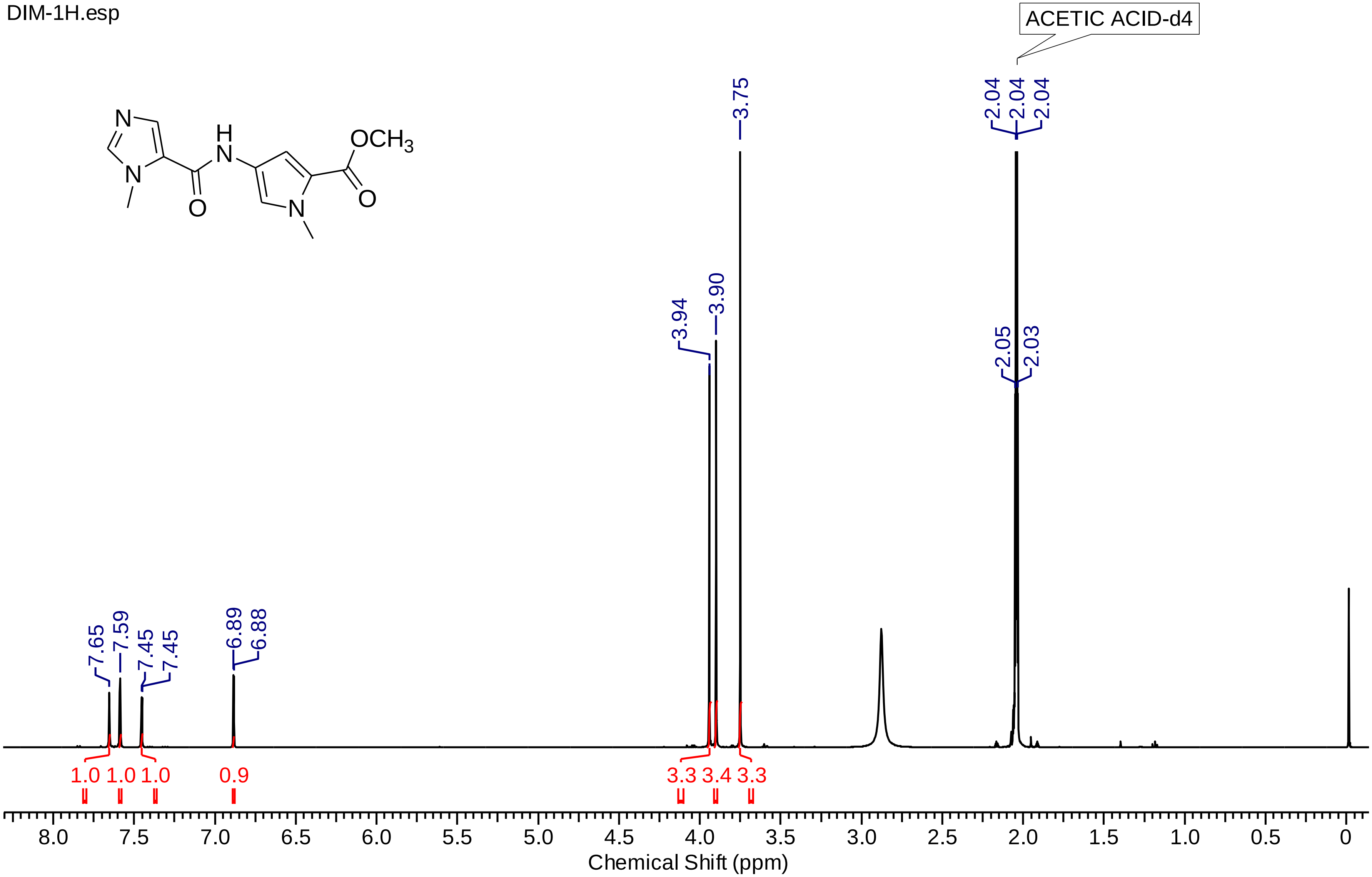

^{13}C NMR (ACETIC ACID-d_4): δ 24.43, 27.07, 41.53, 99.30, 99.36, 110.66, 111.82, 113.89, 123.00, 133.27, 148.66, 152.25; **(6a)**

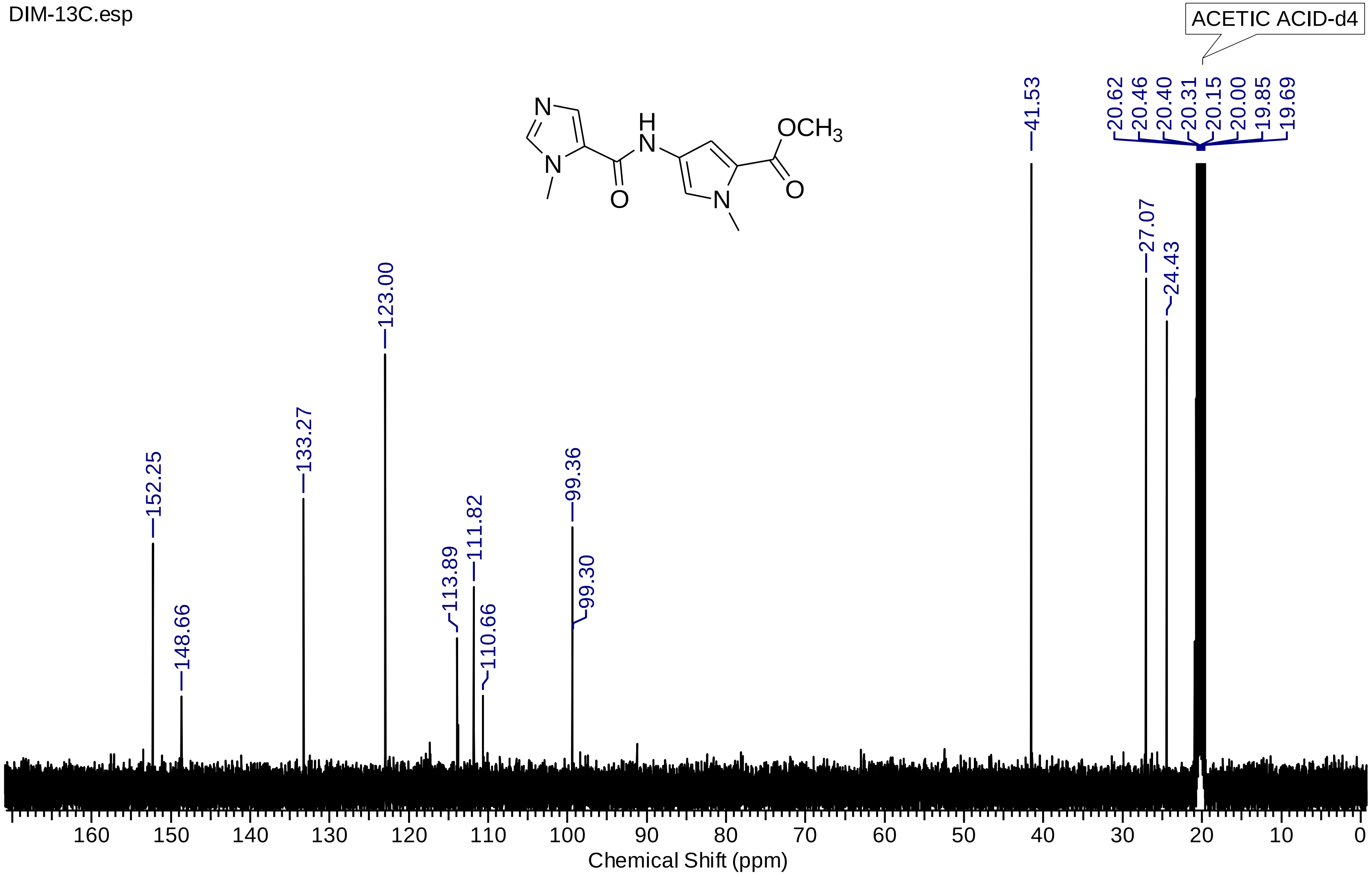

LC−MS (ESI): m/z calcd for $C_{12}H_{14}N_4O_3$, 262.11, found 263.11 [M + H]+. **(6a)**

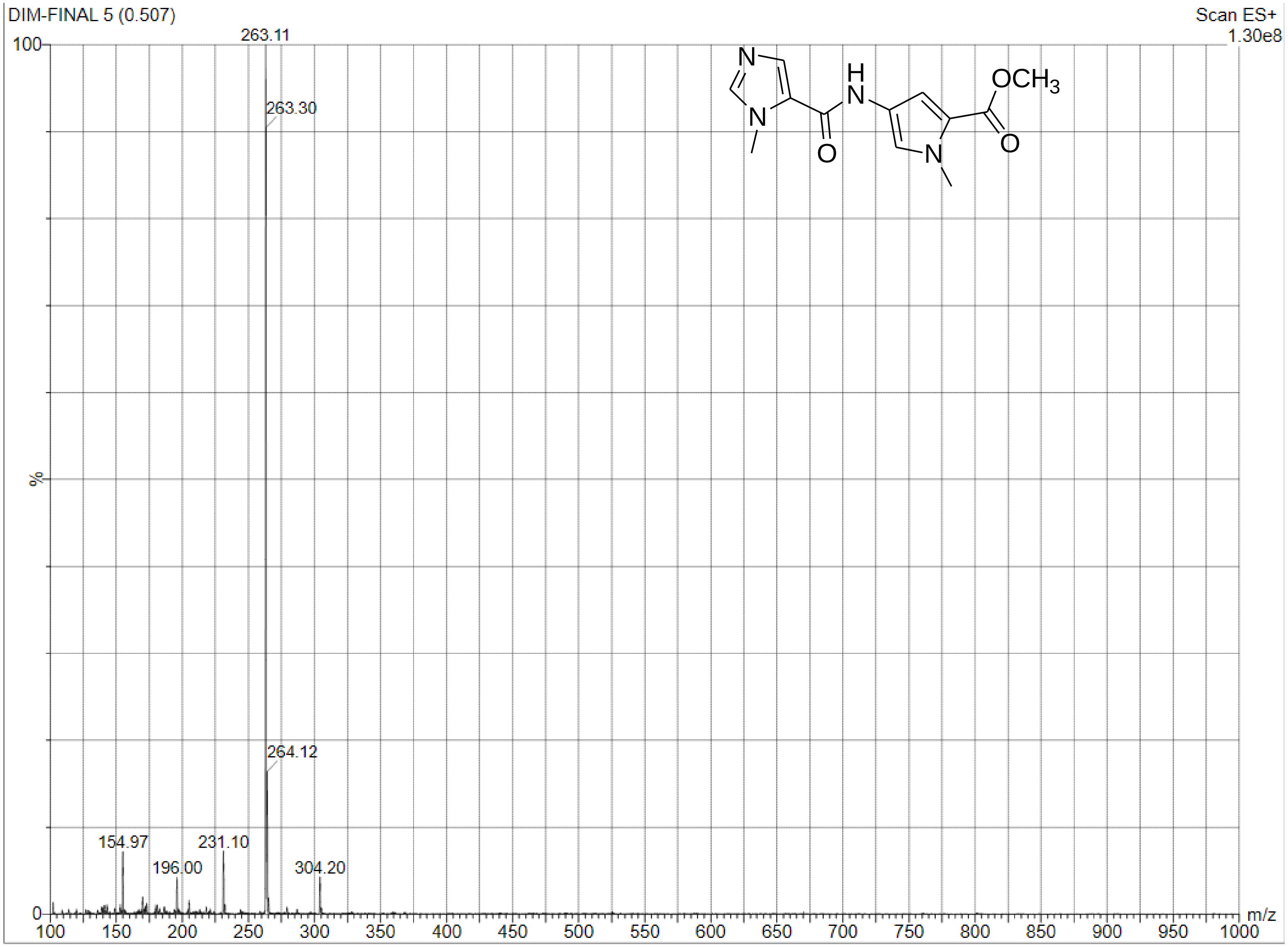

^{1}H NMR (DMSO-d_6) δ 3.82 (3H, s, NCH_3), .98 (3H, s, NCH_3), 6.98 (1H, d, Ar—H), 7.03 (1H, d, Ar—H), 7.38 (1H, d, Ar—H), 7.47 (1H, d, Ar—H), 10.46 (1H, s, COOH) ; **(9)**

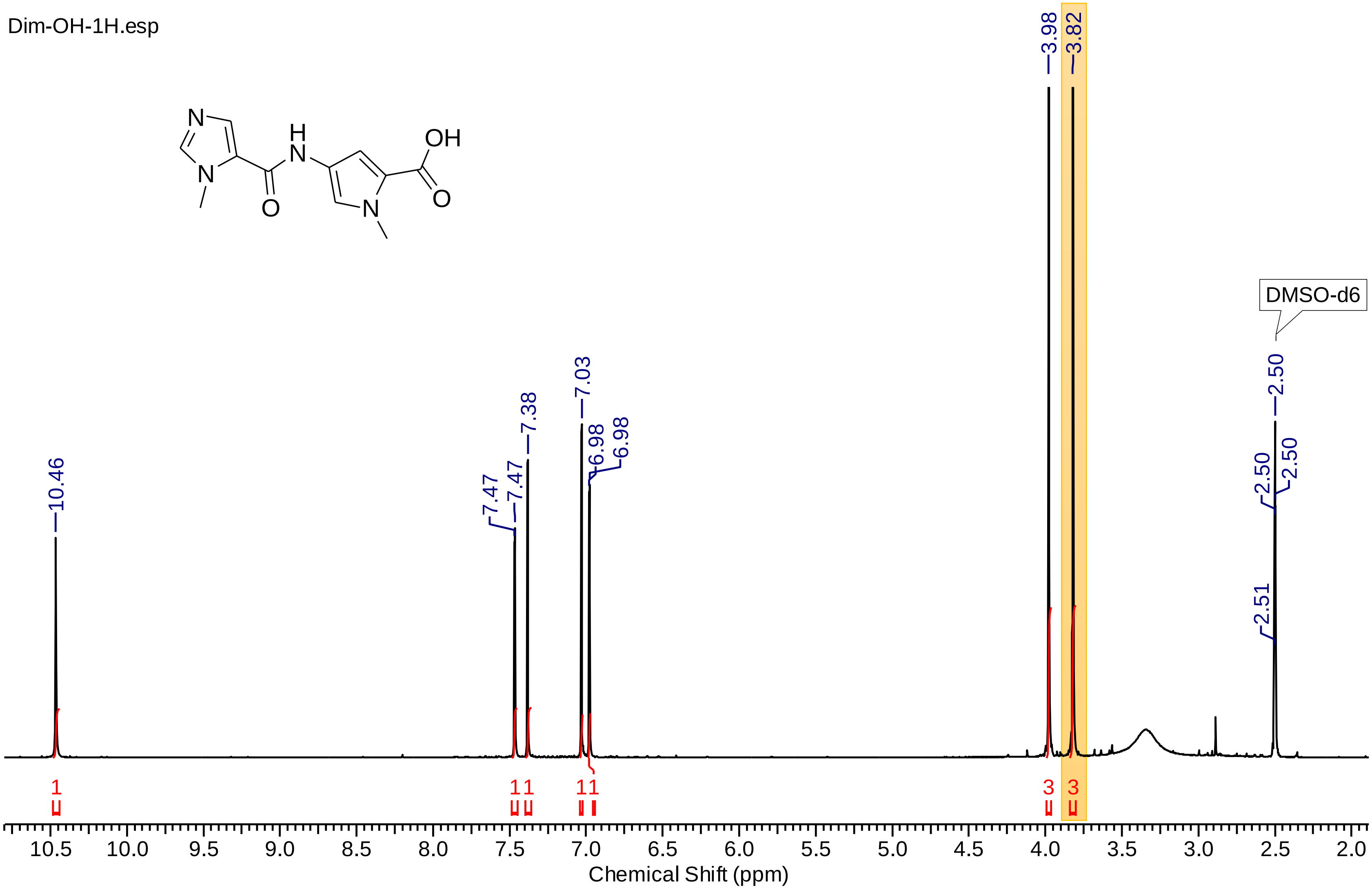

^{13}C NMR (DMSO-d_6): δ 35.10, 36.20, 99.53, 108.85, 119.81, 120.40, 121.91, 126.41, 127.02, 138.64, 156.09, 161.99; **(9)**

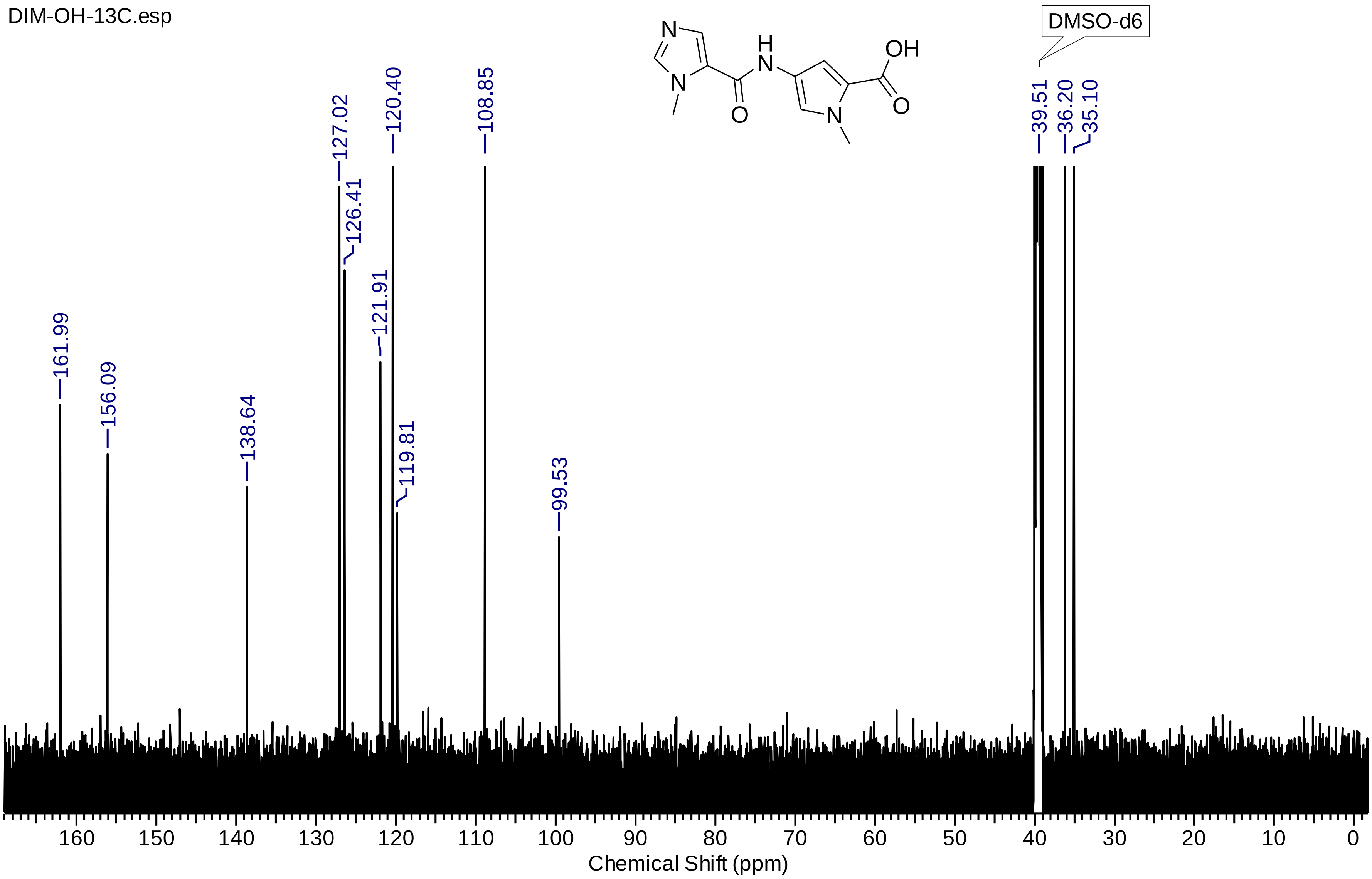

LC−MS (ESI): m/z calcd for $C_{11}H_{12}N_4O_3$, 248.09, found 249.04 [M + H]+. **(9)**

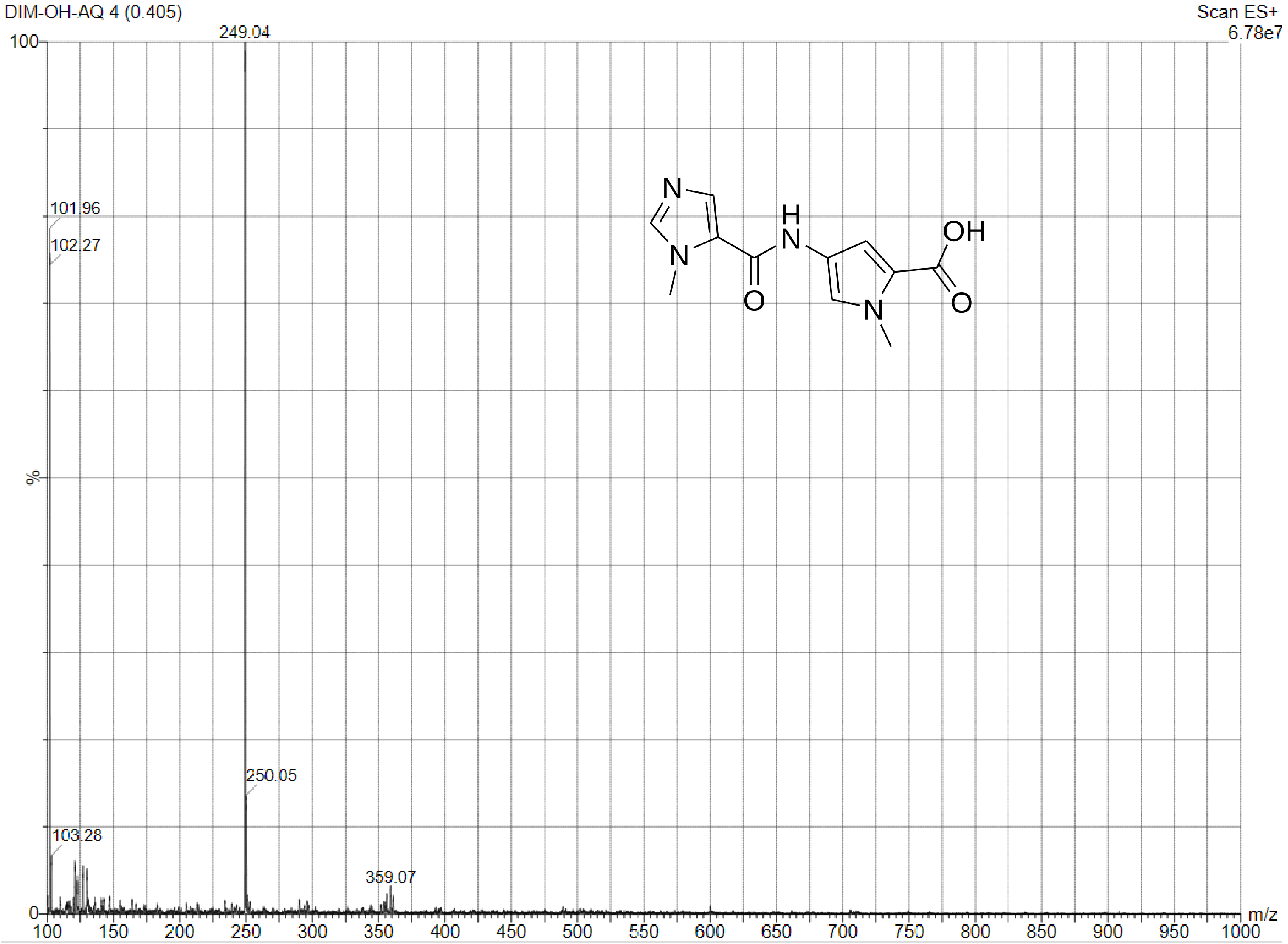

^{1}H NMR (CHLOROFORM-d) δ 3.83 (3H, s, OCH_3), 3.94 (3H, s, NCH_3), 6.85 (1H, d, Ar—H), 7.54 (1H, d, Ar—H), 8.24 (1H, d, Ar—H), 8.81 (1H, d, Ar—H), 9.10 (1H, s, CONH) ; **(6b)**

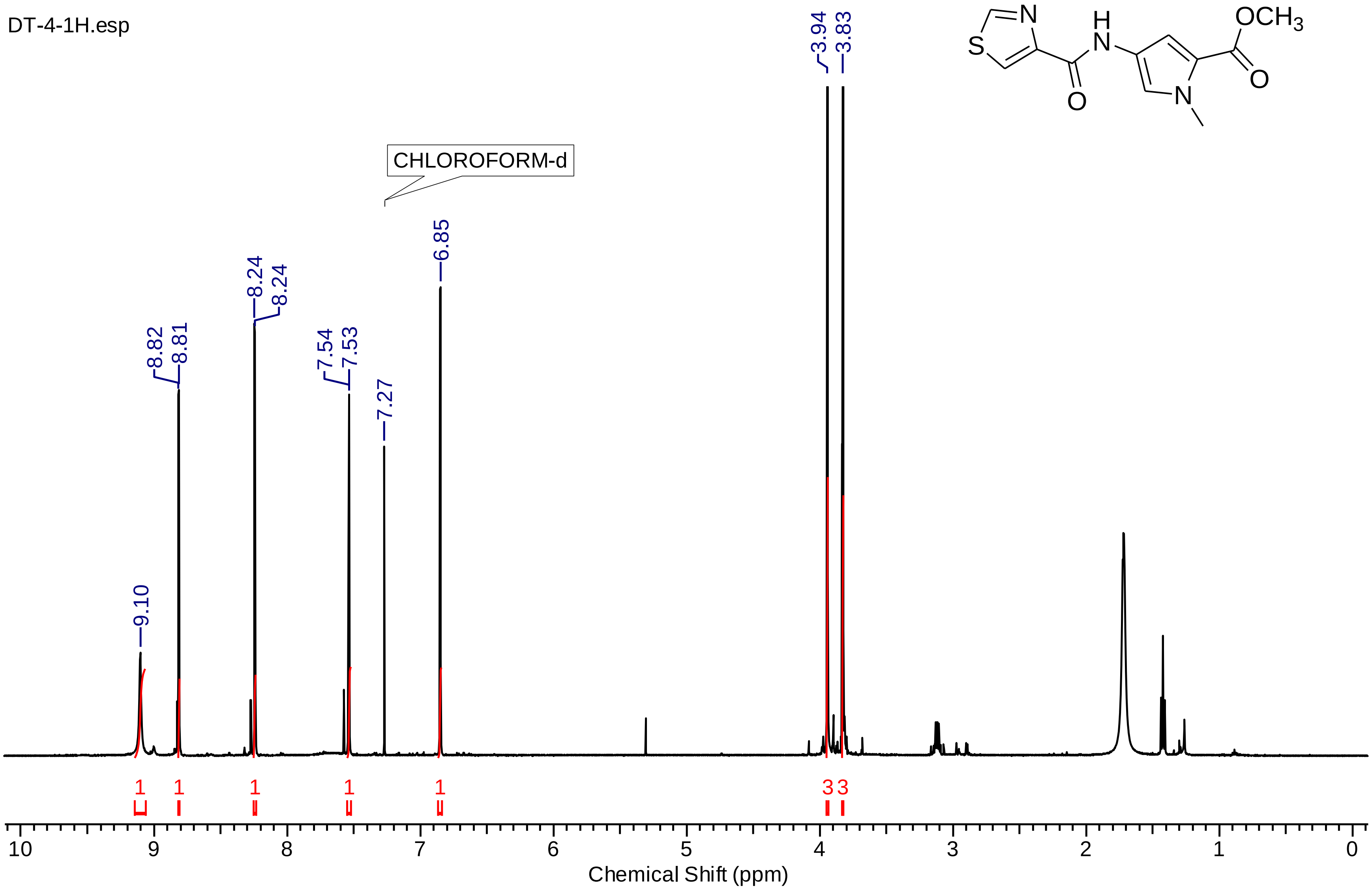

^{13}C NMR (CHLOROFORM-d): δ 36.86, 51.14, 108.20, 120.03, 121.02, 121.22, 123.35, 150.80, 152.79, 157.91, 161.48; **(6b)**

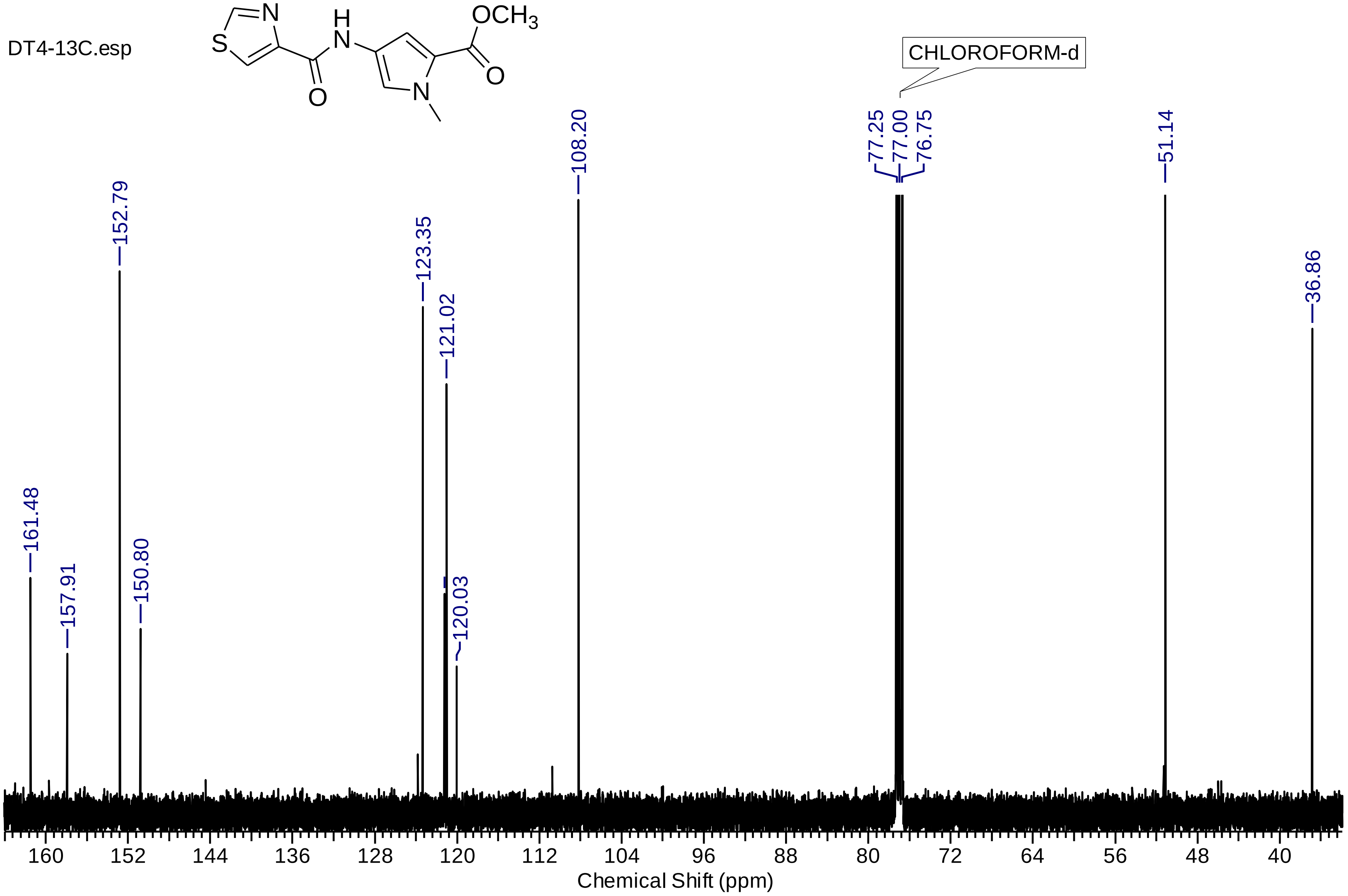

LC−MS (ESI): m/z calcd for $C_{11}H_{11}N_3O_3S$, 265.05, found 266.18 [M + H]+. **(6b)**

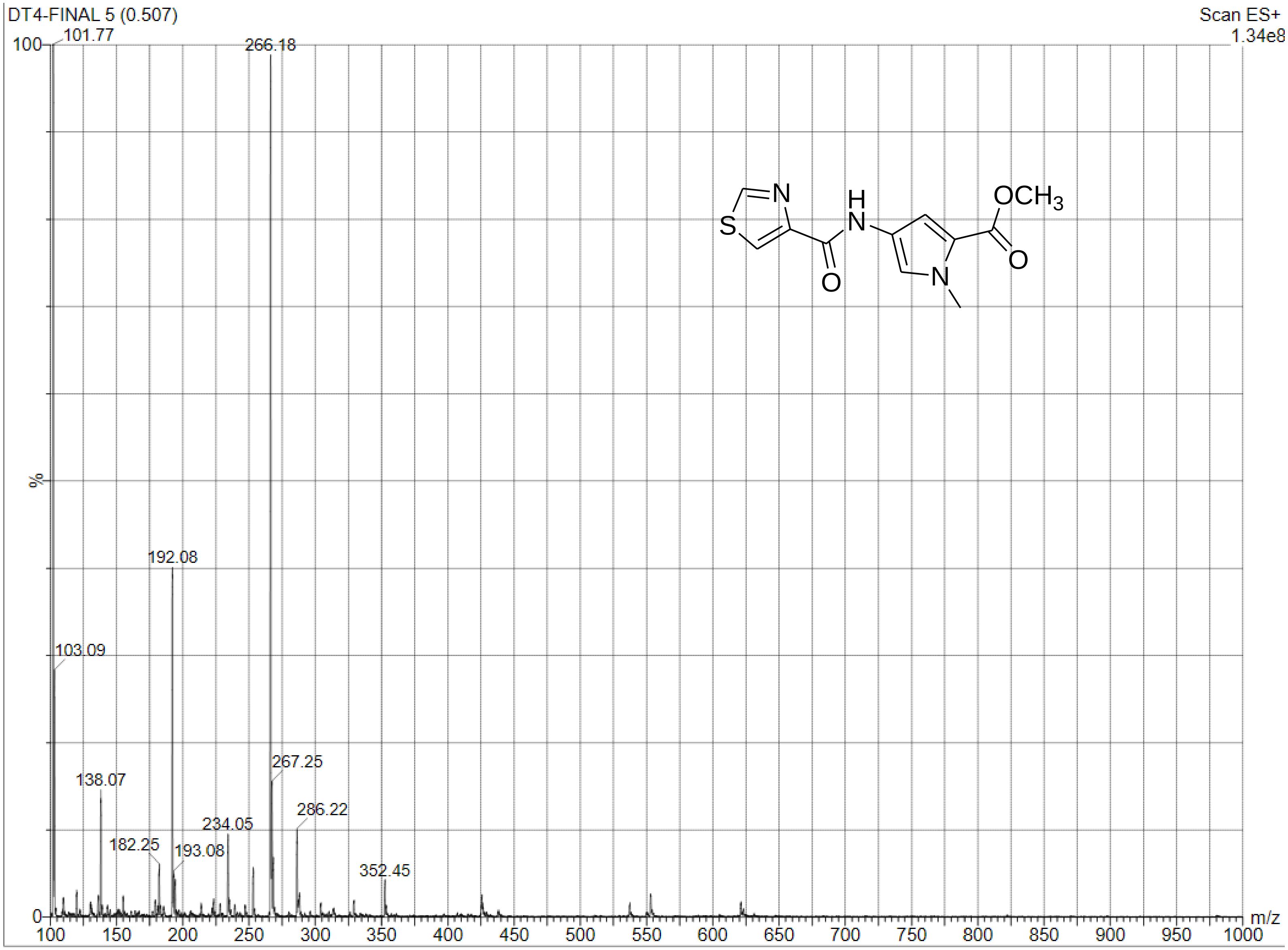

^{1}H NMR (DMSO-d_6) δ 3.83 (3H, s, NCH_3), 7.02 (1H, d, Ar—H), 7.50 (1H, d, Ar—H), 8.39 (1H, d, Ar—H), 9.23 (1H, d, Ar—H), 10.49 (1H, s, COOH), 12.21 (1H, s, CONH) ; **(10)**

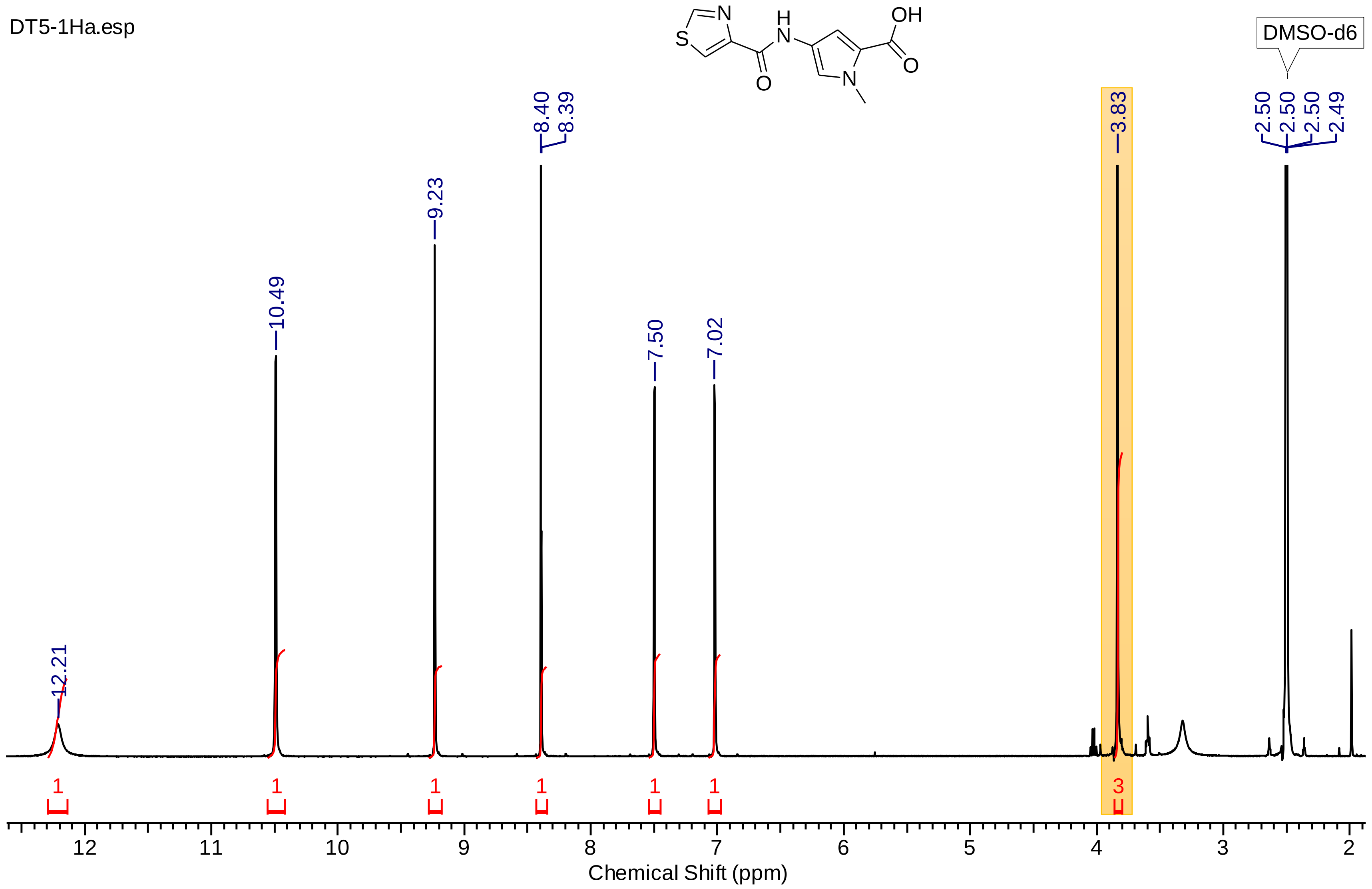

^{13}C NMR (DMSO-d_6): δ 25.13, 36.23, 109.04, 120.61, 121.99, 124.58, 150.71, 154.93, 157.76, 161.97; **(10)**

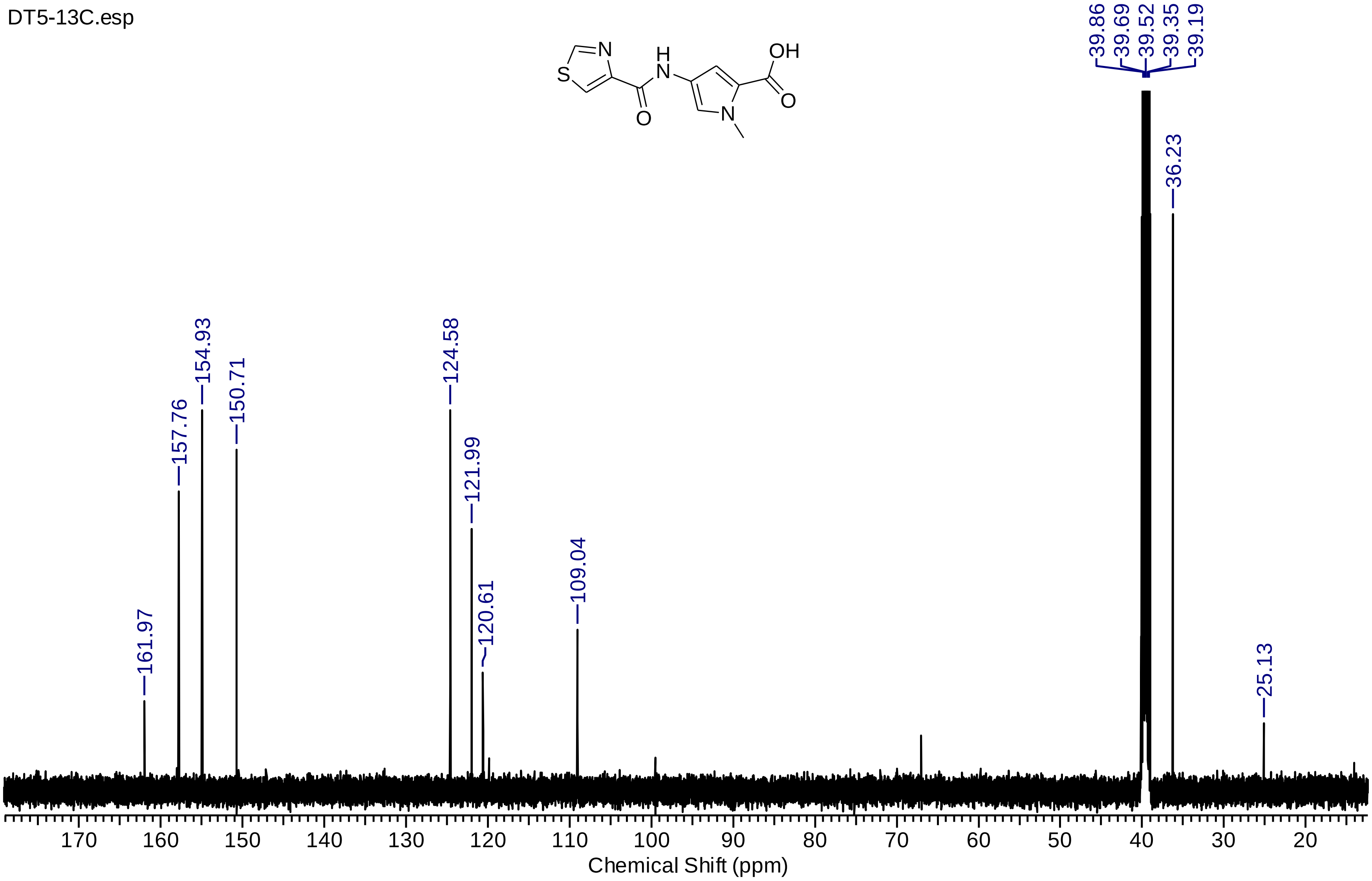

LC−MS (ESI): m/z calcd for $C_{10}H_9N_3O_3S$, 251.04, found 251.89 [M + H]+. **(10)**

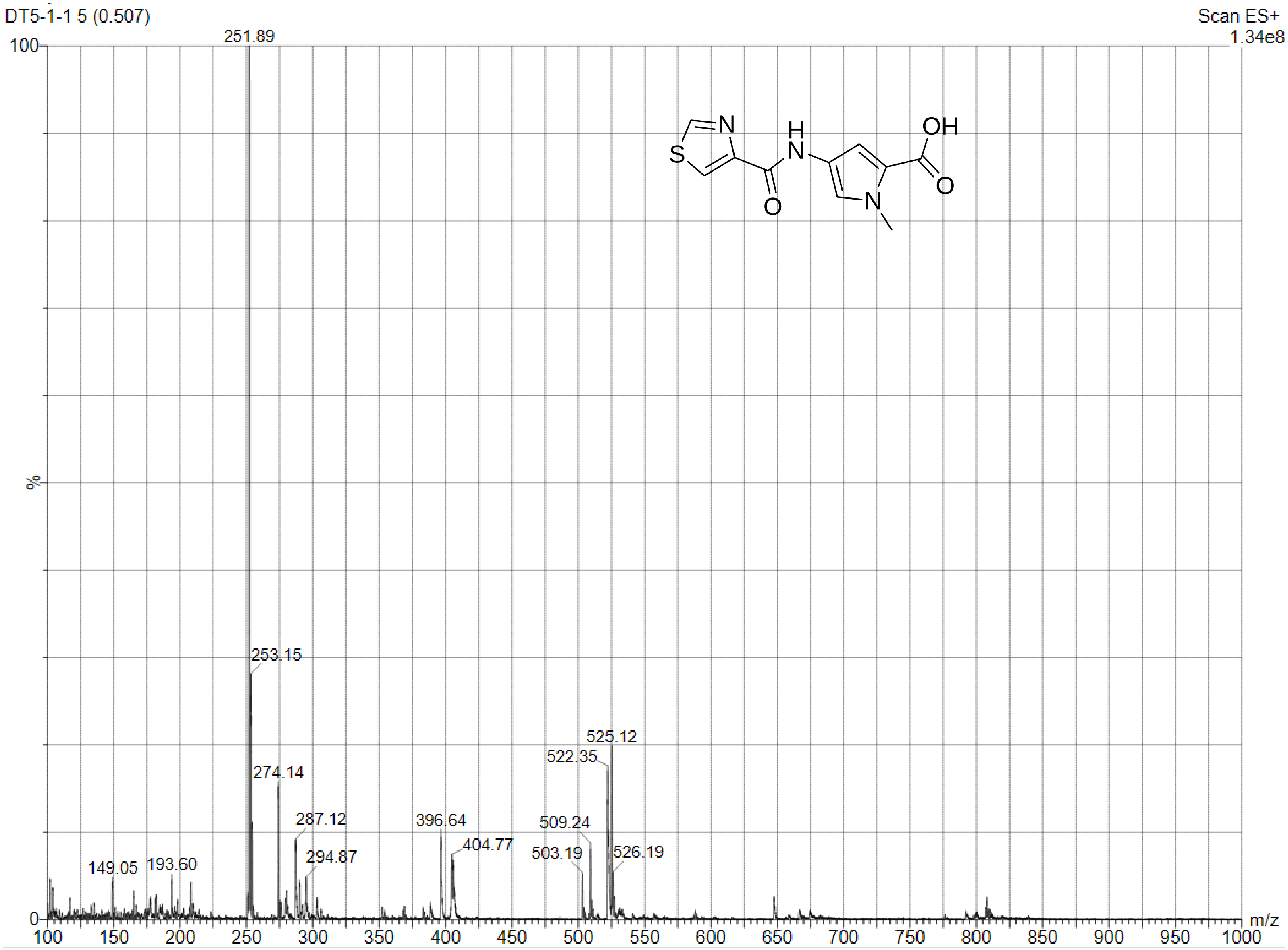

^{1}H NMR (DMSO-d_6) δ 3.91 (3H, s, NCH_3), 7.26 (1H, d, Ar—H), 8.23 (1H, d, Ar—H) ; **(4a)**

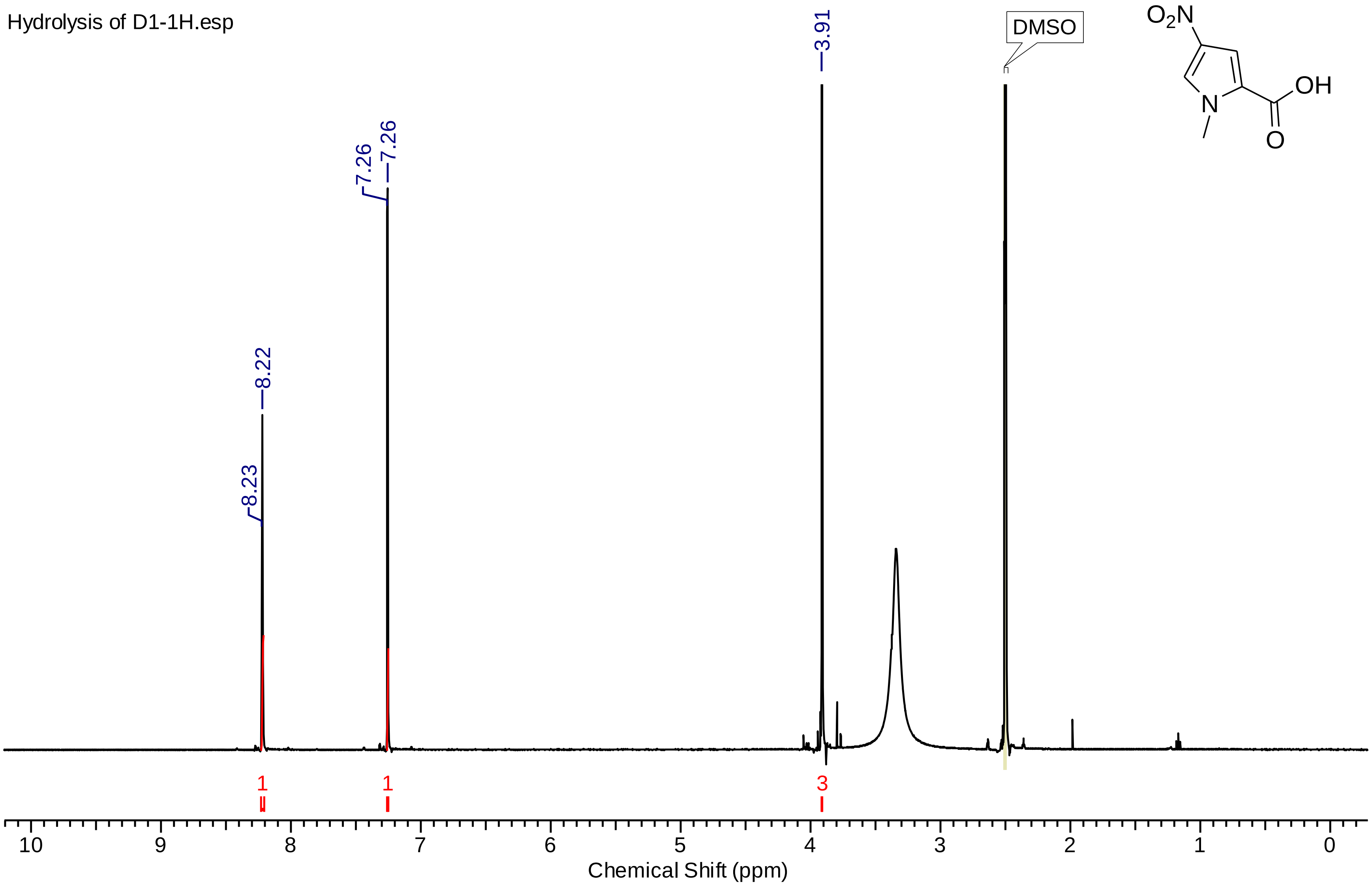

^{13}C NMR (DMSO-d_6): δ 37.50, 111.41, 123.81, 129.21, 134.02, 160.99; **(4a)**

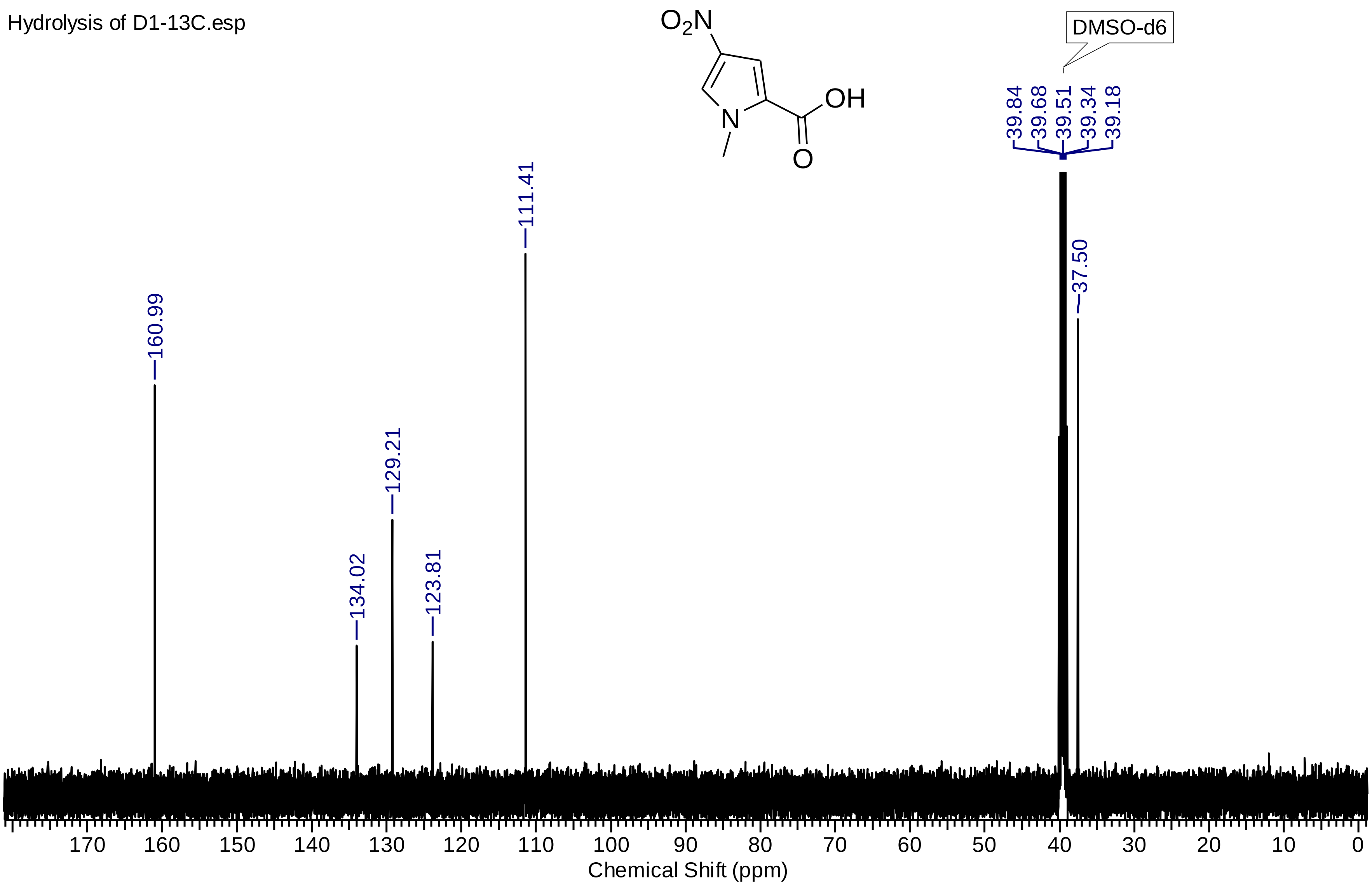

LC−MS (ESI): m/z calcd for $C_6H_6N_2O_4$, 170.03, found 169.01 [M - H]_. **(4a)**

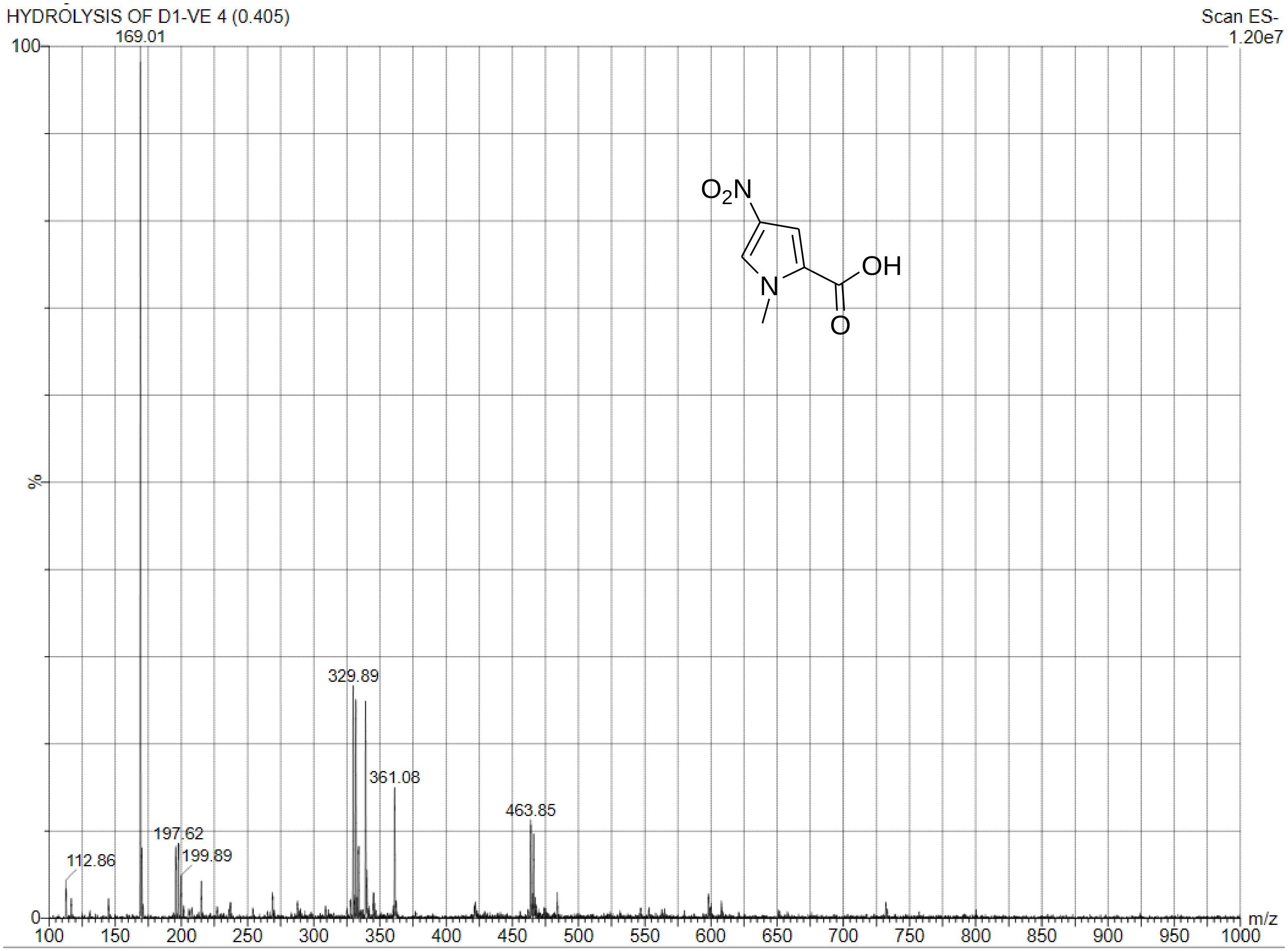

^{1}H NMR (CHOLOROFORM-d) δ 1.38 (6H, d, CH_3), 3.89 (3H, s, OCH_3), 4.09 (3H, d, NCH_3), 4.14 (1H, m, CH), 7.30 (1H, d, AR—H), 7.68 (1H, d, Ar—H); **(3)**

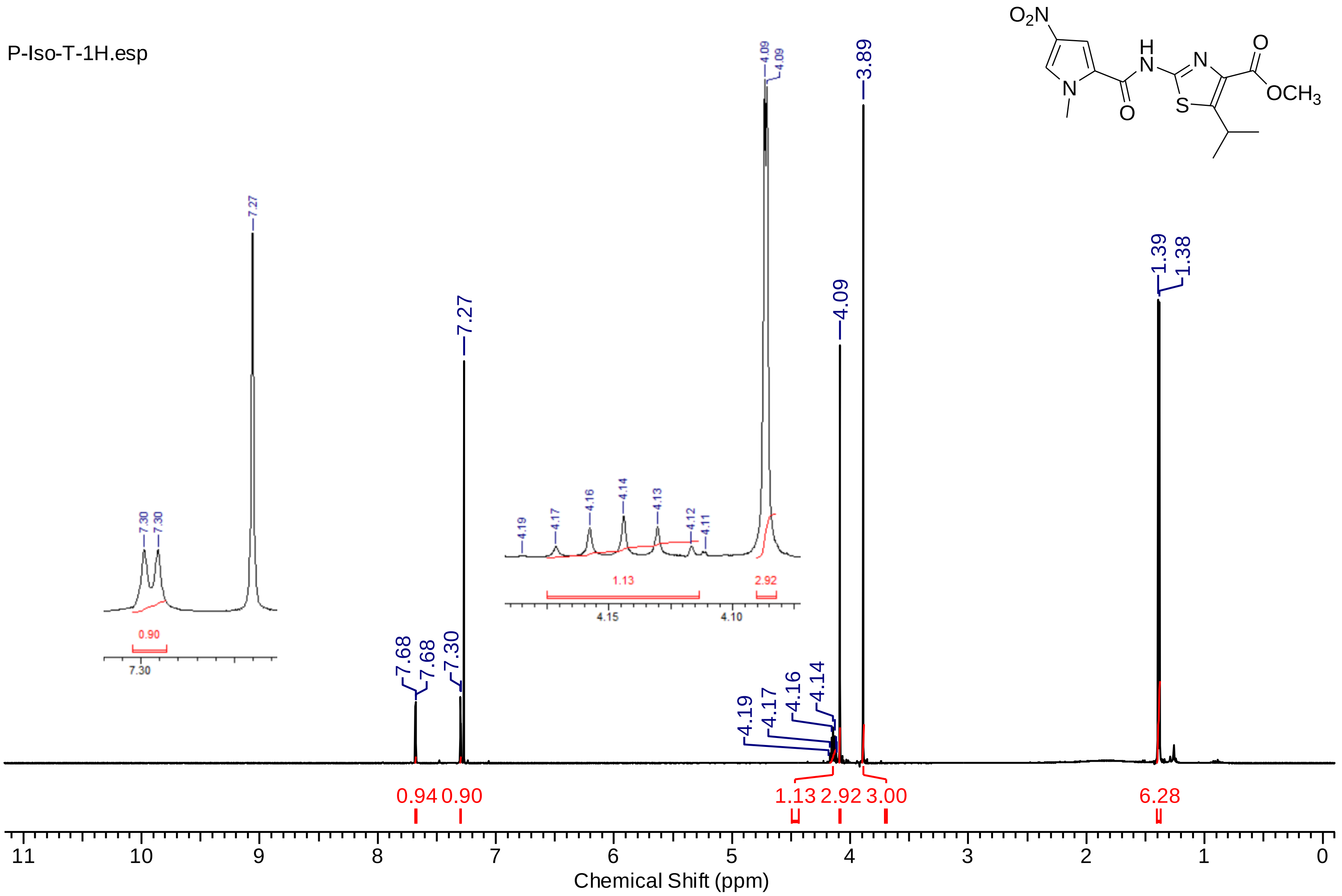

^{13}C NMR (CHLOROFORM-d): δ 24.85, 27.79, 38.52, 52.41, 100.13, 109.95, 123.69, 128.58, 135.69, 153.30, 154.46, 157.85, 161.83; **(3)**

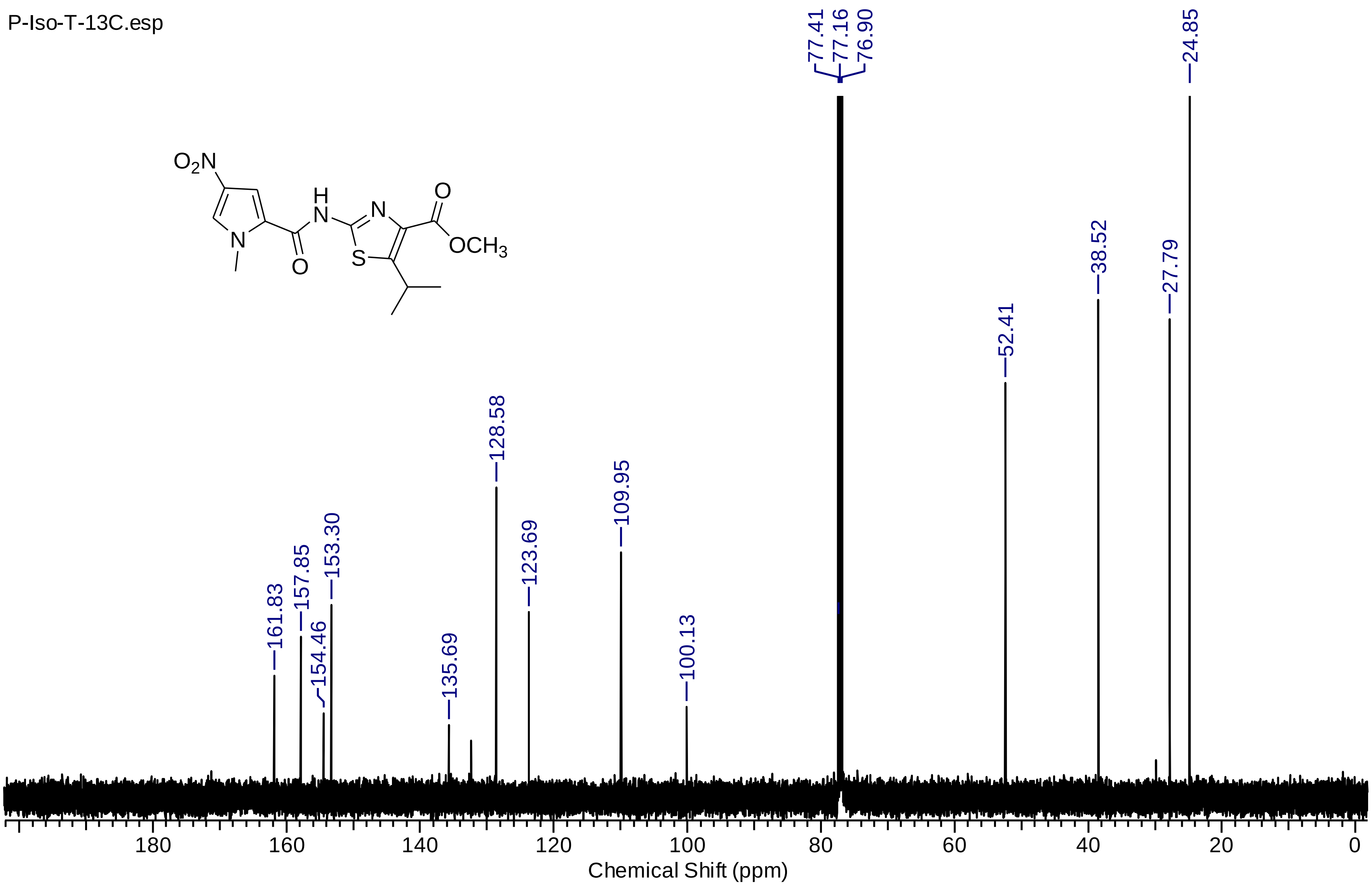

LC−MS (ESI): m/z calcd for $C_{14}H_{16}N_4O_4S$, 352.08, found 353.02 [M + H]+. **(3)**

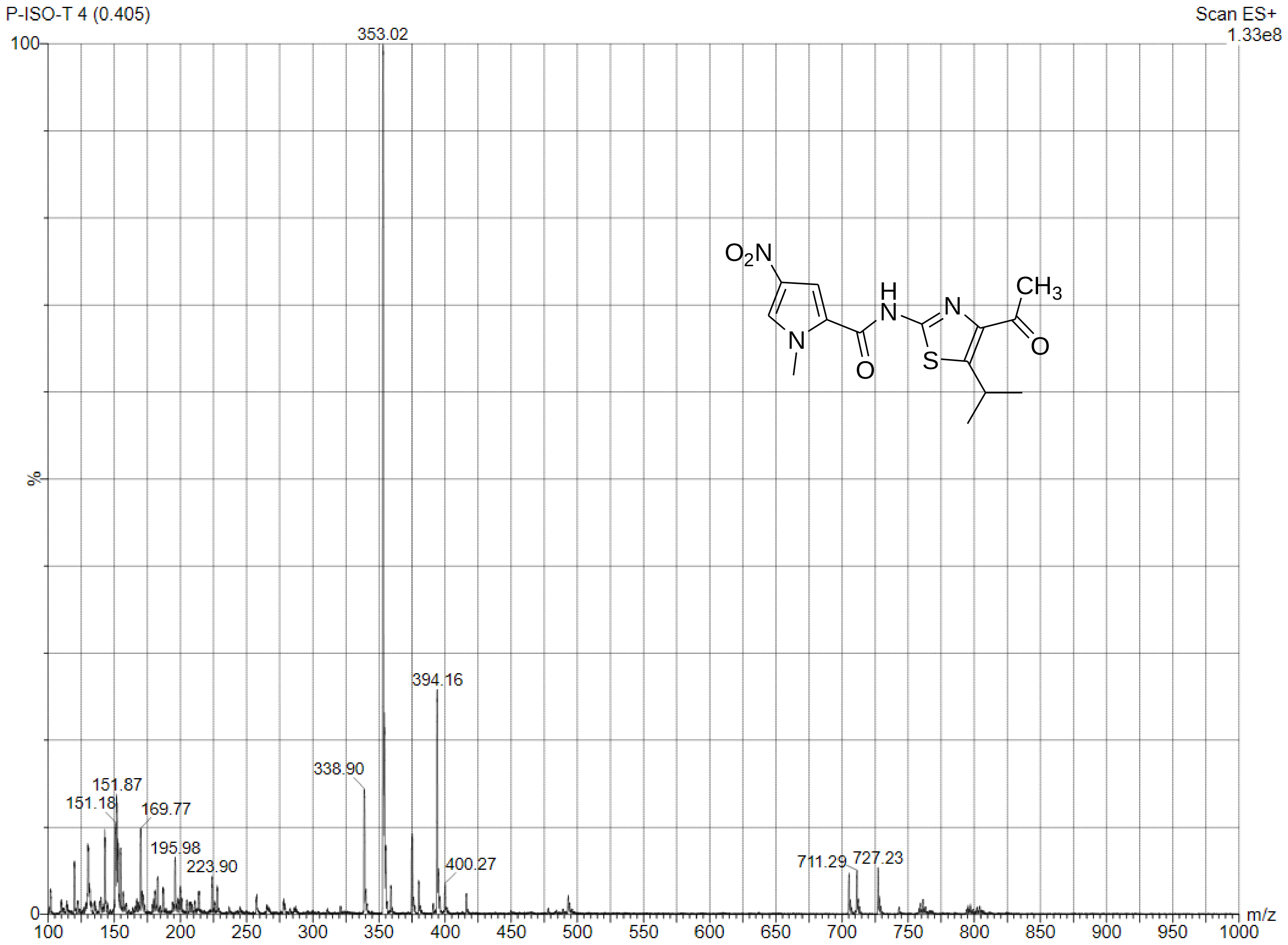

^{1}H NMR (CHOLOROFORM-d) δ 1.33 (6H, d, CH_3), 1.8 (2H, m, CH_2), 2.26 (6H, s, NCH_3), 2.41 (2H, t, NCH_2), 3.45 (2H, q, CONH--CH_2), 4.10 (3H, d, NCH_3), 4.41 (1H, m, CH), 7.55 (1H, t, CONH), 7.64 (1H, d, Ar—H), 7.70 (1H, d, Ar—H) ; **(5)**

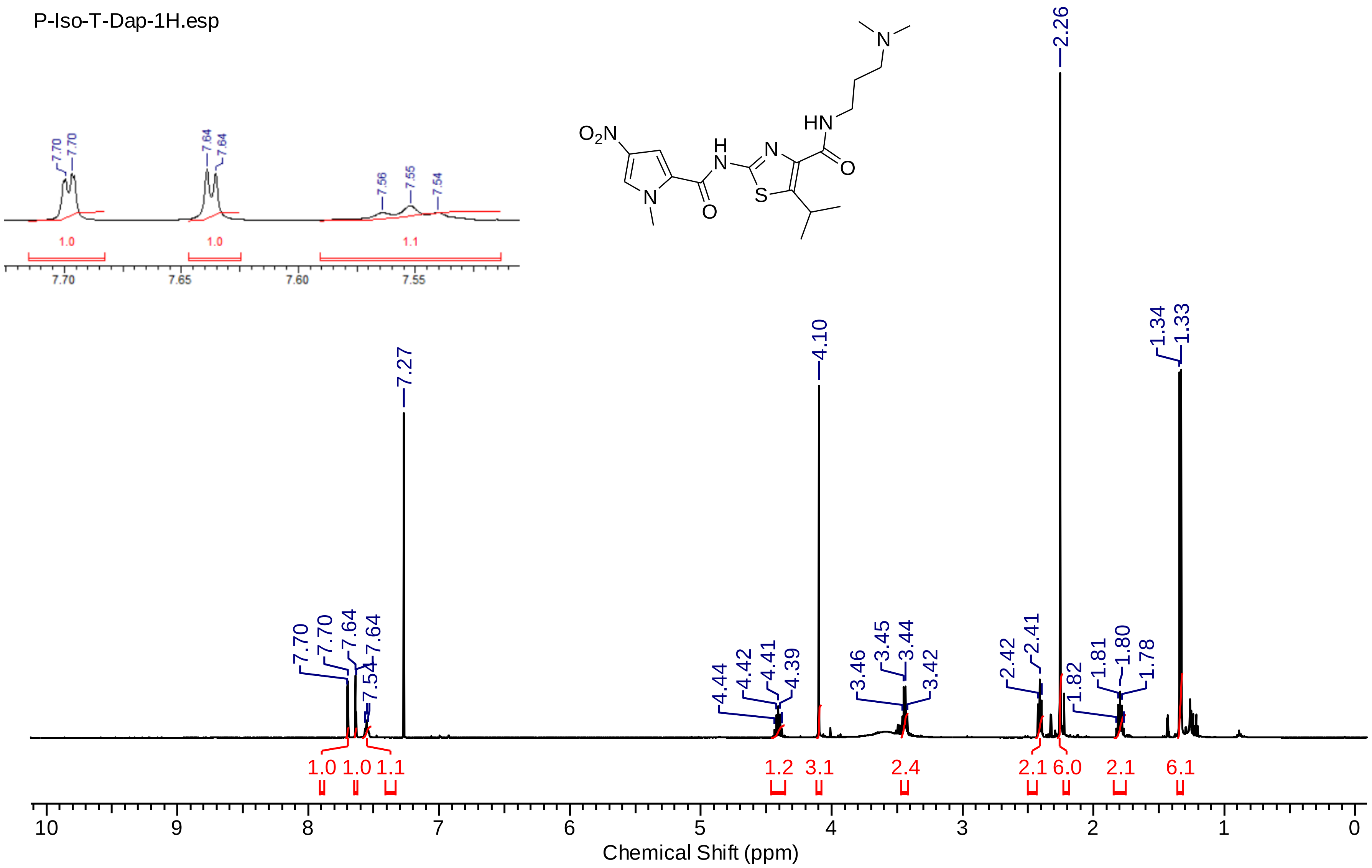

^{13}C NMR (CHLOROFORM-d): δ 25.12, 27.23, 27.47, 37.71, 38.59, 45.61, 57.56, 109.71, 124.19, 128.38, 135.55, 136.07, 148.99, 152.78, 157.92, 162.86; **(5)**

P-Iso-T-Dap-13C.esp

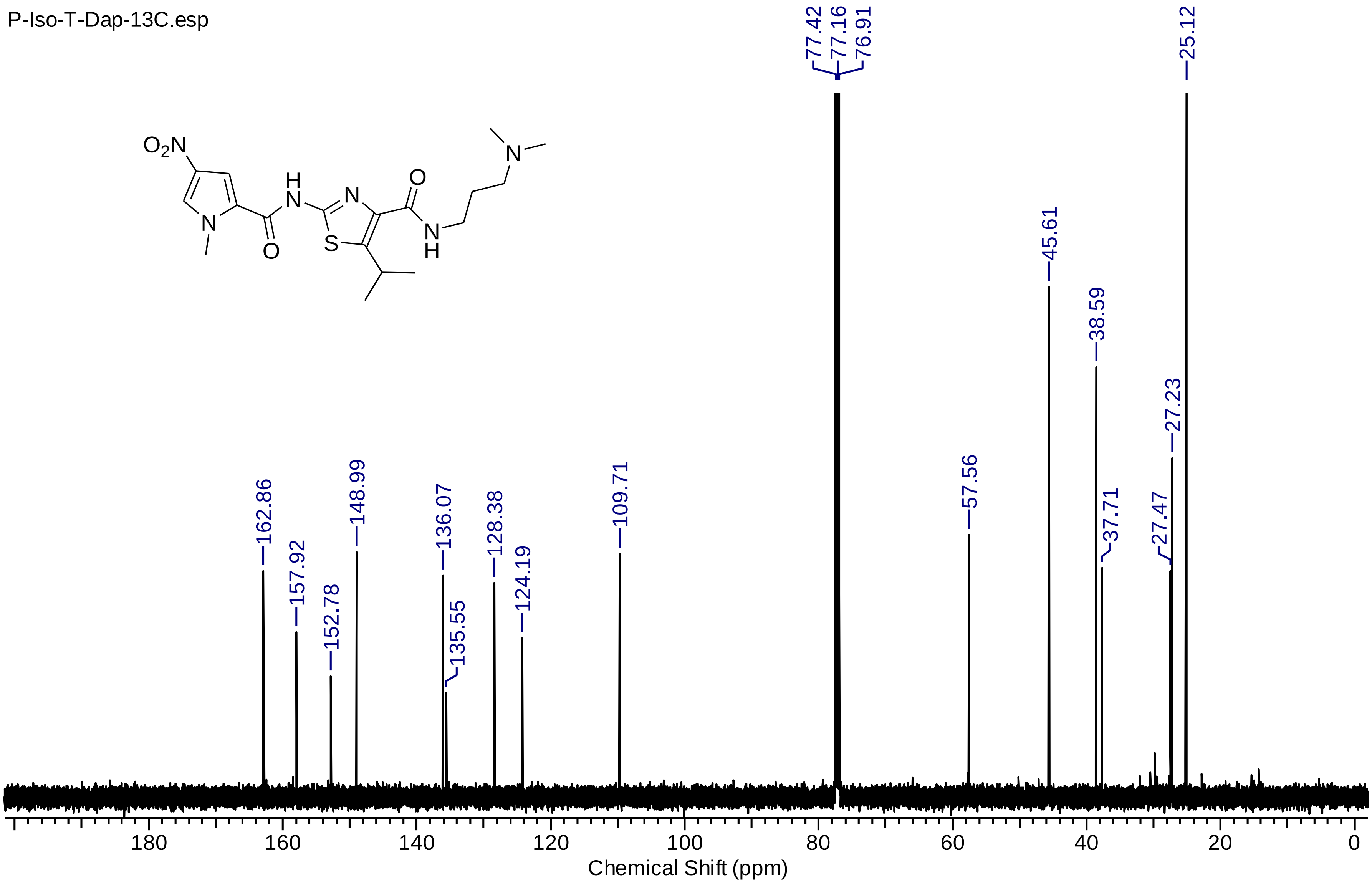

LC−MS (ESI): m/z calcd for $C_{18}H_{26}N_6O_4S$, 422.17, found 423.08 [M + H]+. **(5)**

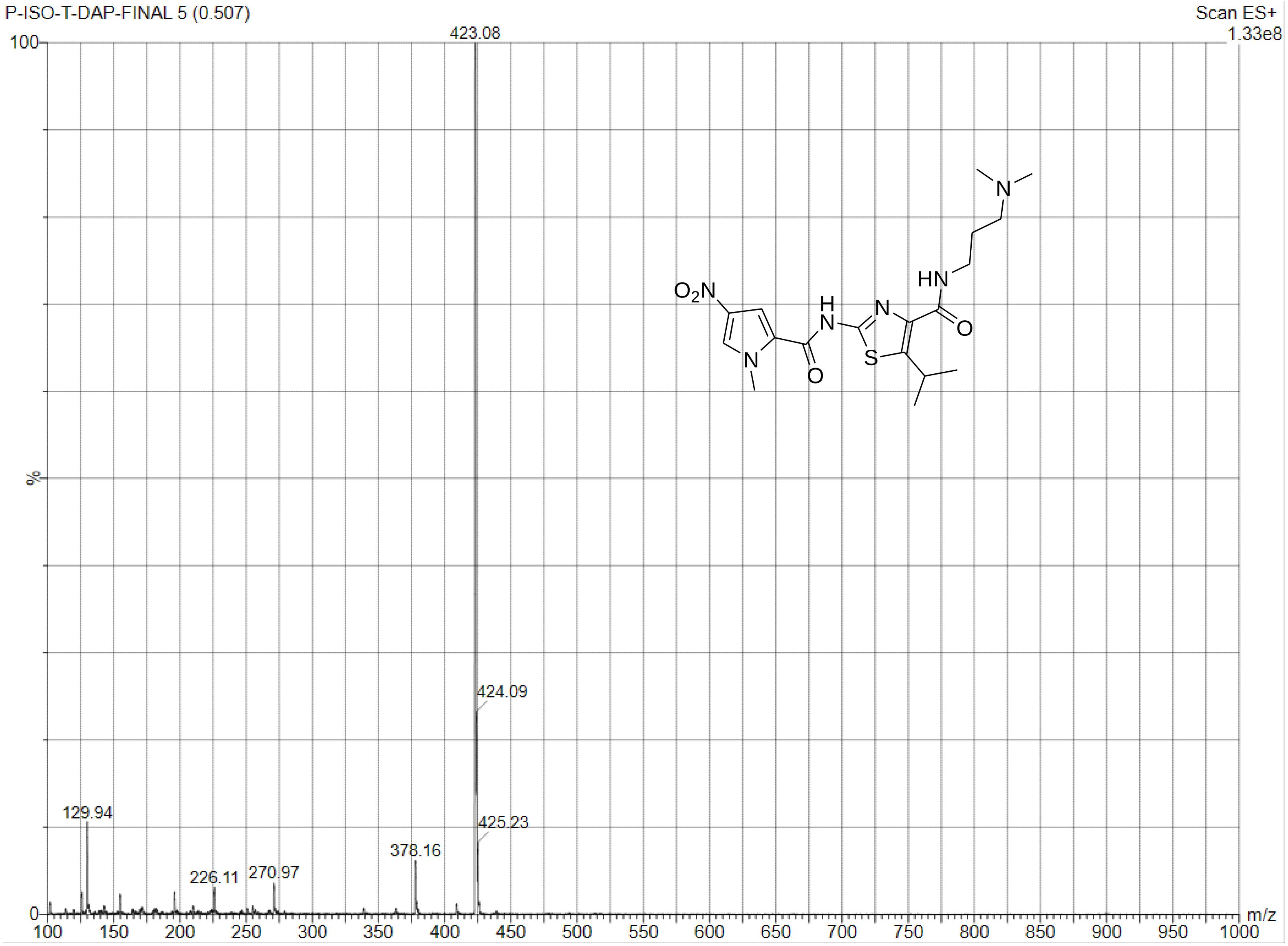

^{1}H NMR (DMSO-d_6) δ 1.28 (6H, d, CH_3), 3.98 (3H, s, NCH_3), 4.06 (1H, m, CH), 7.98 (1H, s, AR—H), 8.28 (1H, s, Ar—H) ; **(4)**

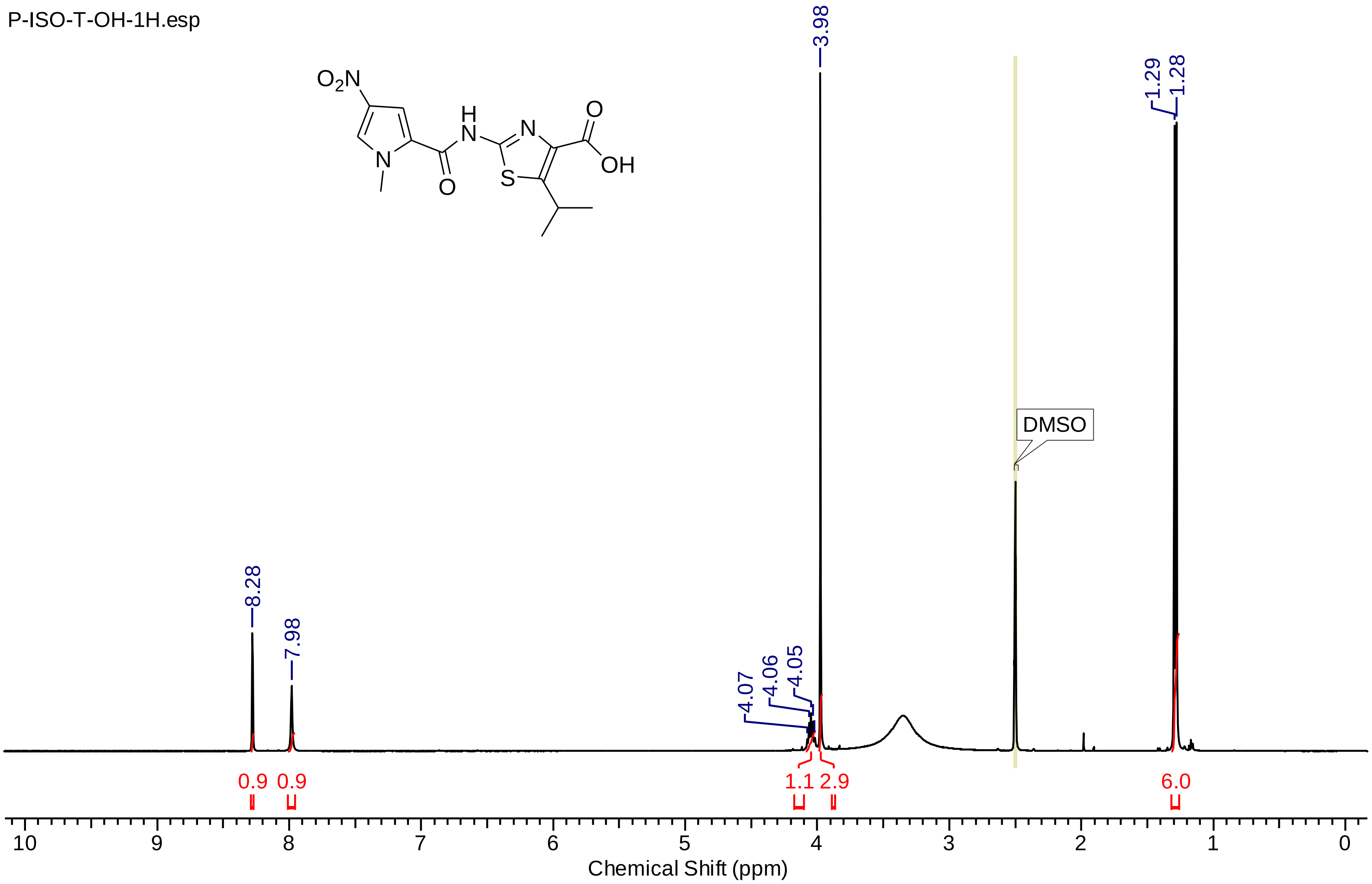

^{13}C NMR ($DMSO-d_6$): δ 24.72, 26.90, 37.99, 110.44, 123.88, 129.73, 134.18, 134.92, 149.92, 153.42, 158.34, 163.54; **(4)**

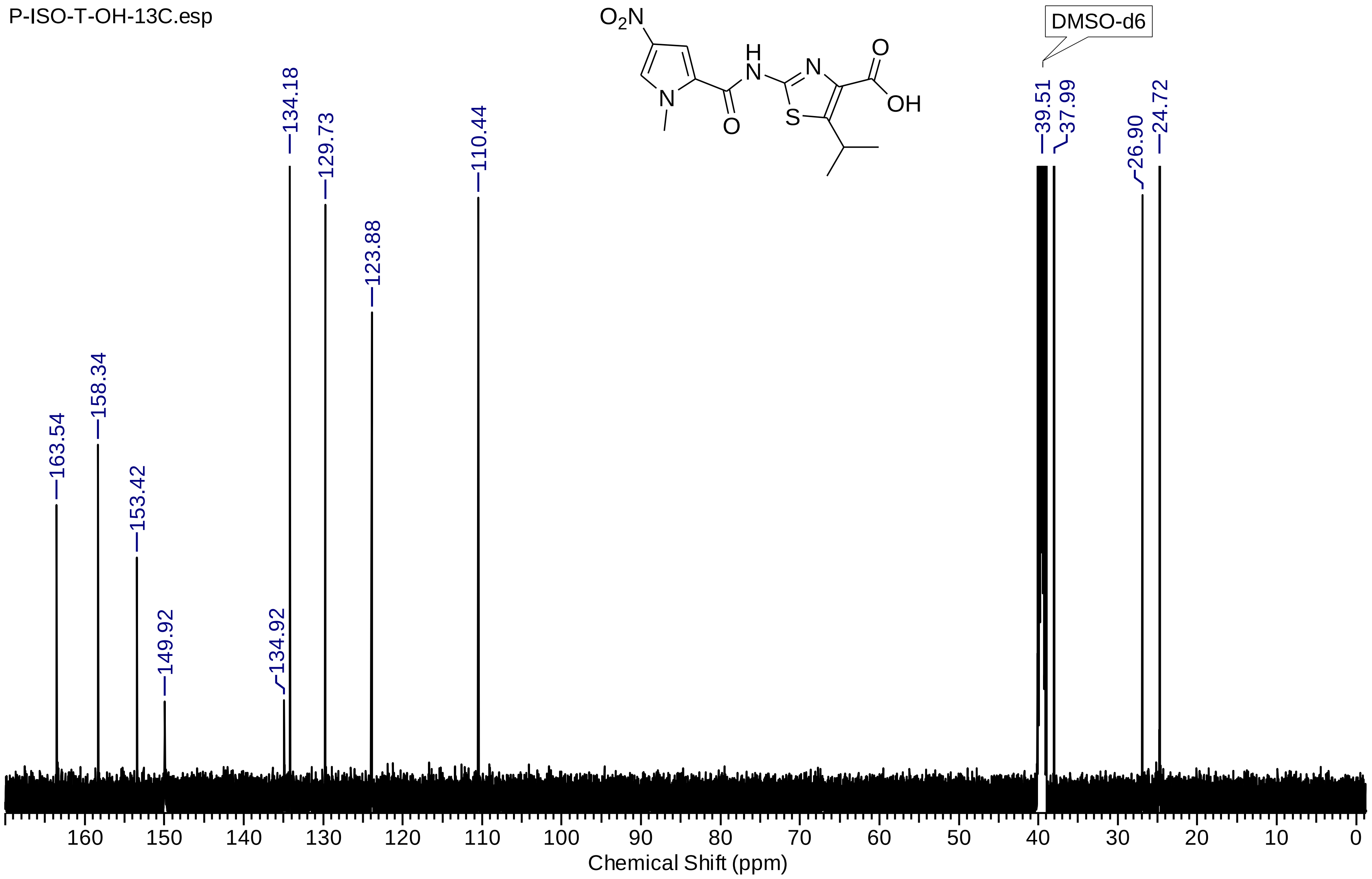

LC−MS (ESI): m/z calcd for $C_{13}H_{14}N_4O_5S$, 338.7, found 339.23 [M + H]+. **(4)**

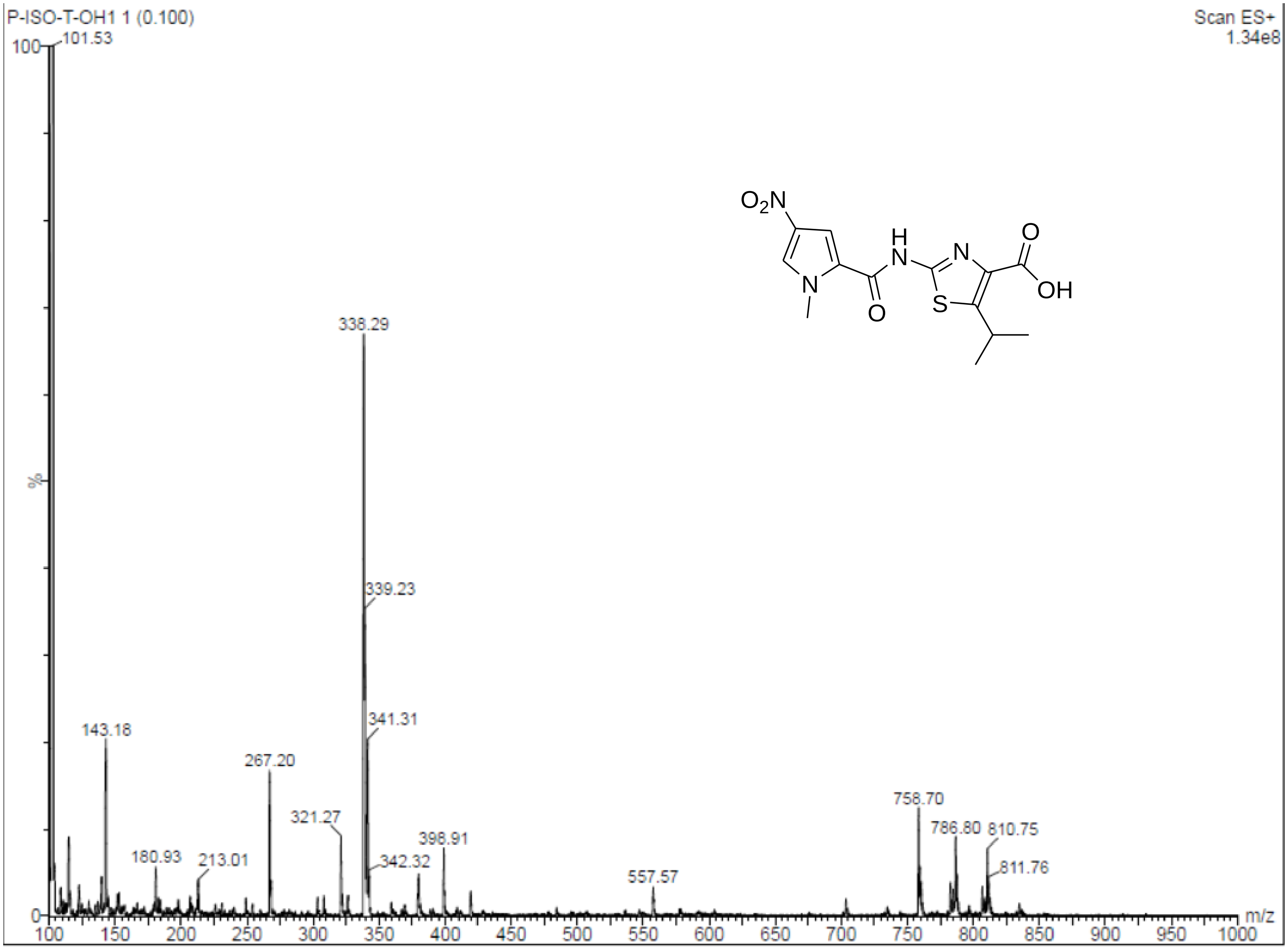

^{1}H NMR (CHOLOROFORM-d) δ 1.84 (2H, m, CH_2), 2.44 (6H, s, NCH_3), 2.65 (2H, t, NCH_2), 3.48 (2H, q, CONH--CH_2), 3.93 (3H, s, NCH_3), 4.04 (3H, s, NCH_3), 6.58 (1H, d, Ar—H), 7.25 (1H, d, Ar—H), 7.32 (1H, d, Ar—H), 7.60 (1H, d, Ar—H), 7.76 (1H, s, CONH), 8.11 (1H, s, CONH) ; **(7)**

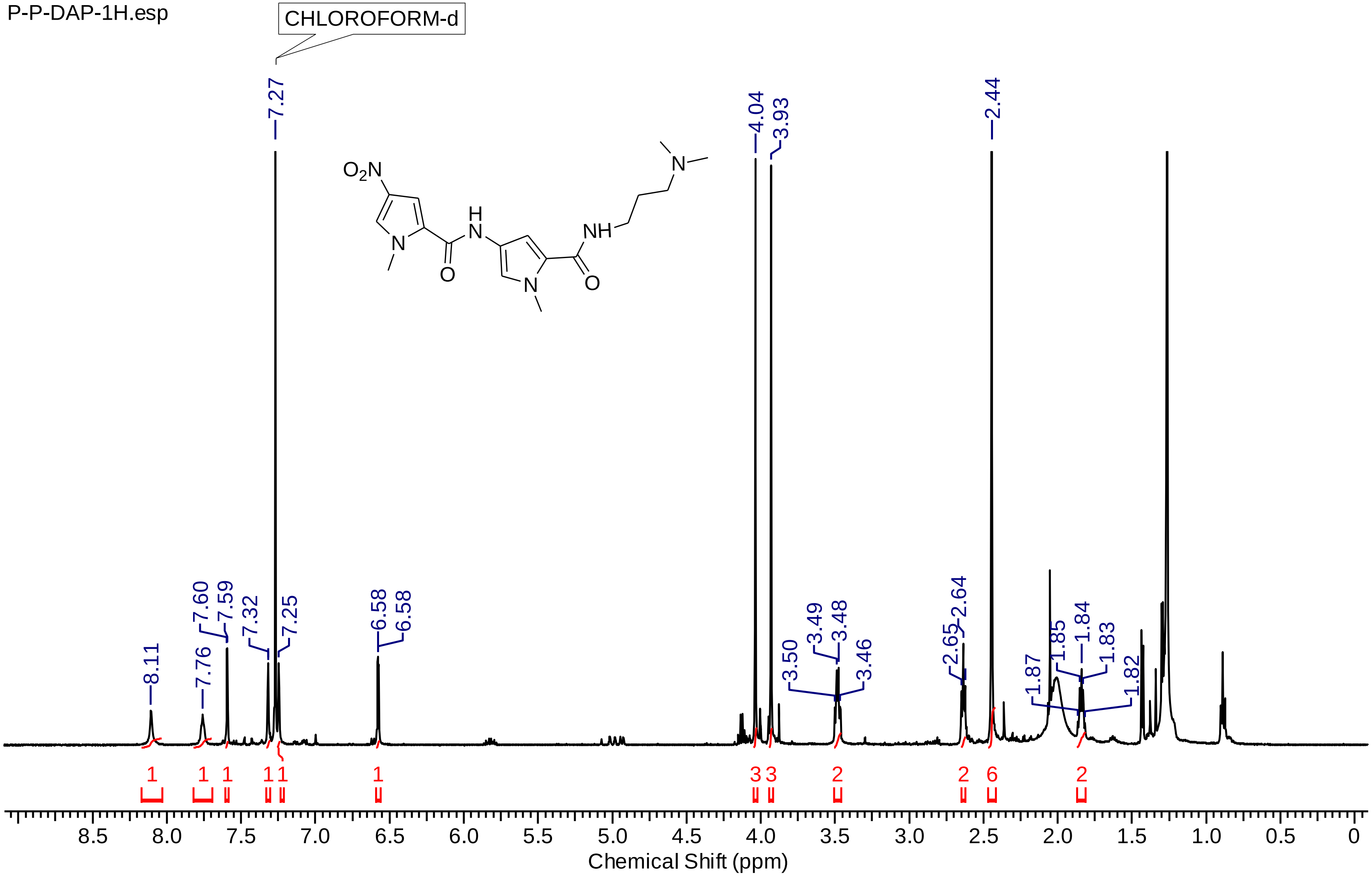

^{13}C NMR (CHLOROFORM-d): δ 14.27, 22.84, 25.58, 29.51, 29.85, 32.08, 36.88, 38.10, 45.19, 103.1, 107.15, 118.89, 120.64, 124.22, 127.03, 161.75; **(7)**

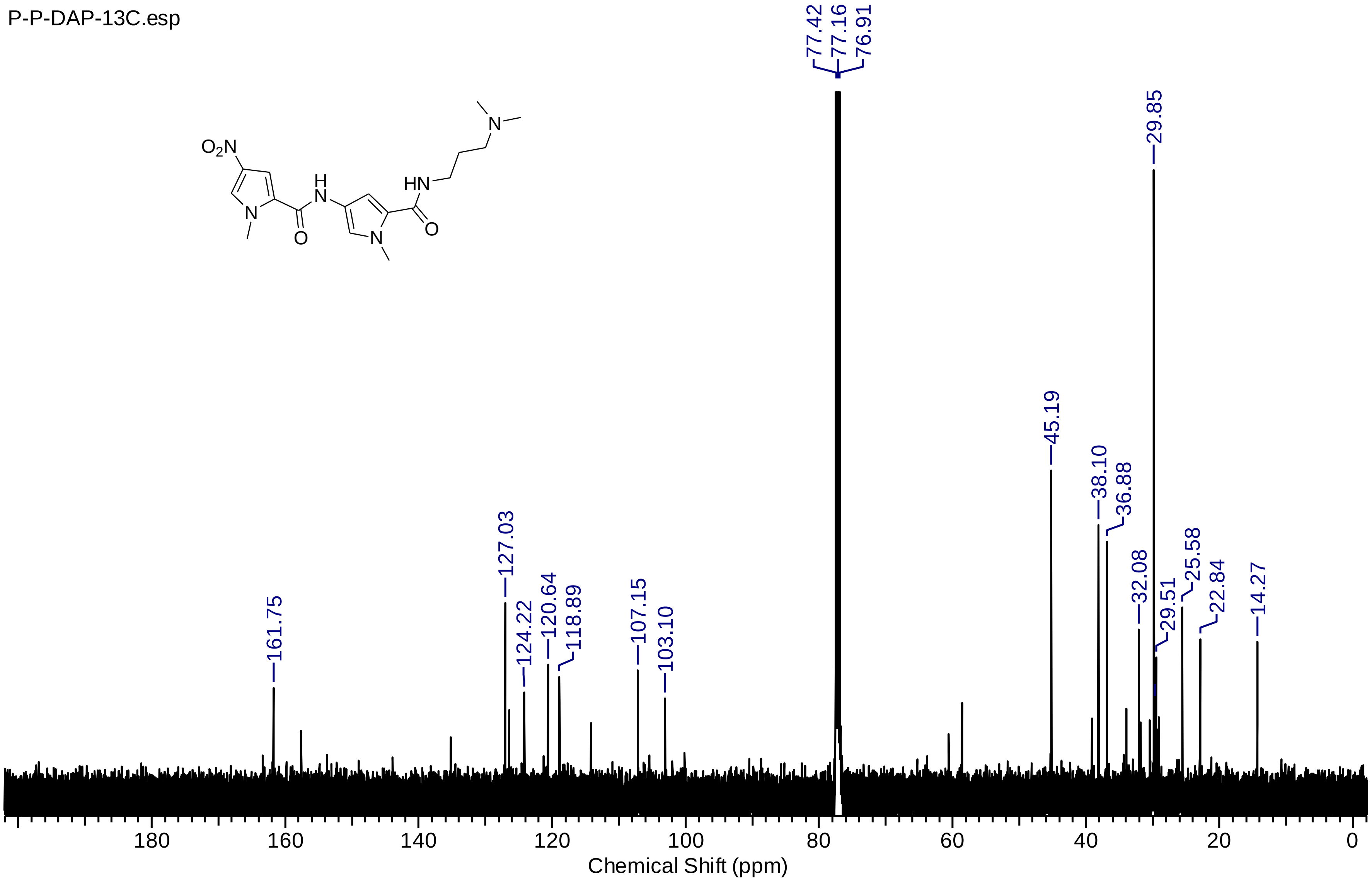

LC−MS (ESI): m/z calcd for $C_{17}H_{24}N_6O_4$, 376.19, found 377.02 [M + H]+. **(7)**

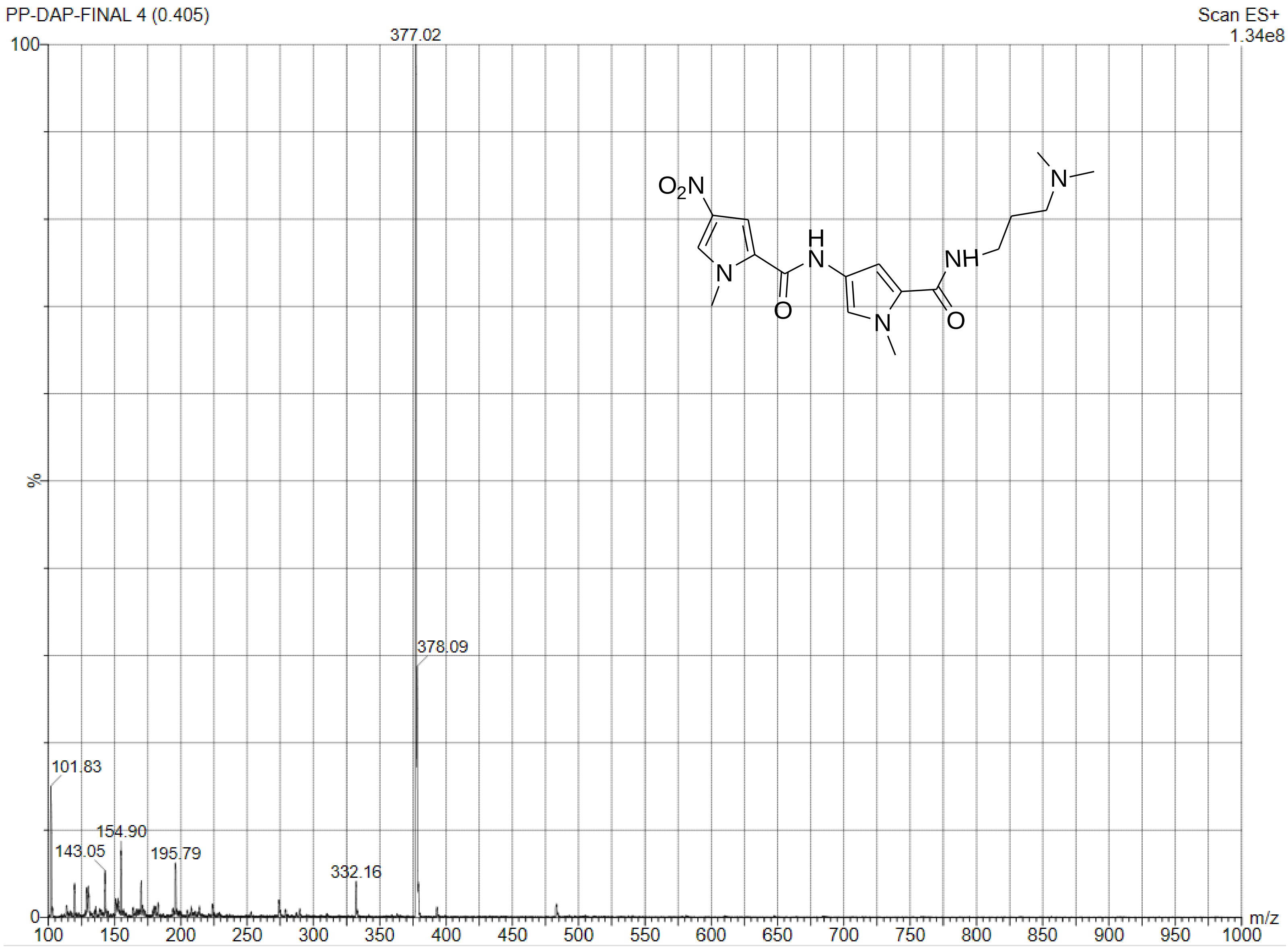

^{1}H NMR (ACETIC ACID-d_4) δ 2.01 (2H, t, NCH_2), 3.04 (4H, s, NCH_2), 3.51 (2H, q, CONH--CH_2), 3.90 (4H, s, OCH_2) 3.93 (3H, s, NCH_3), 4.07 (3H, s, NCH_3), 6.98 (1H, d, Ar—H), 7.28 (1H, d, Ar—H), 7.34 (1H, d, Ar—H), 7.94 (1H, d, Ar—H), 9.52 (1H, s, CONH) ; **(8)**

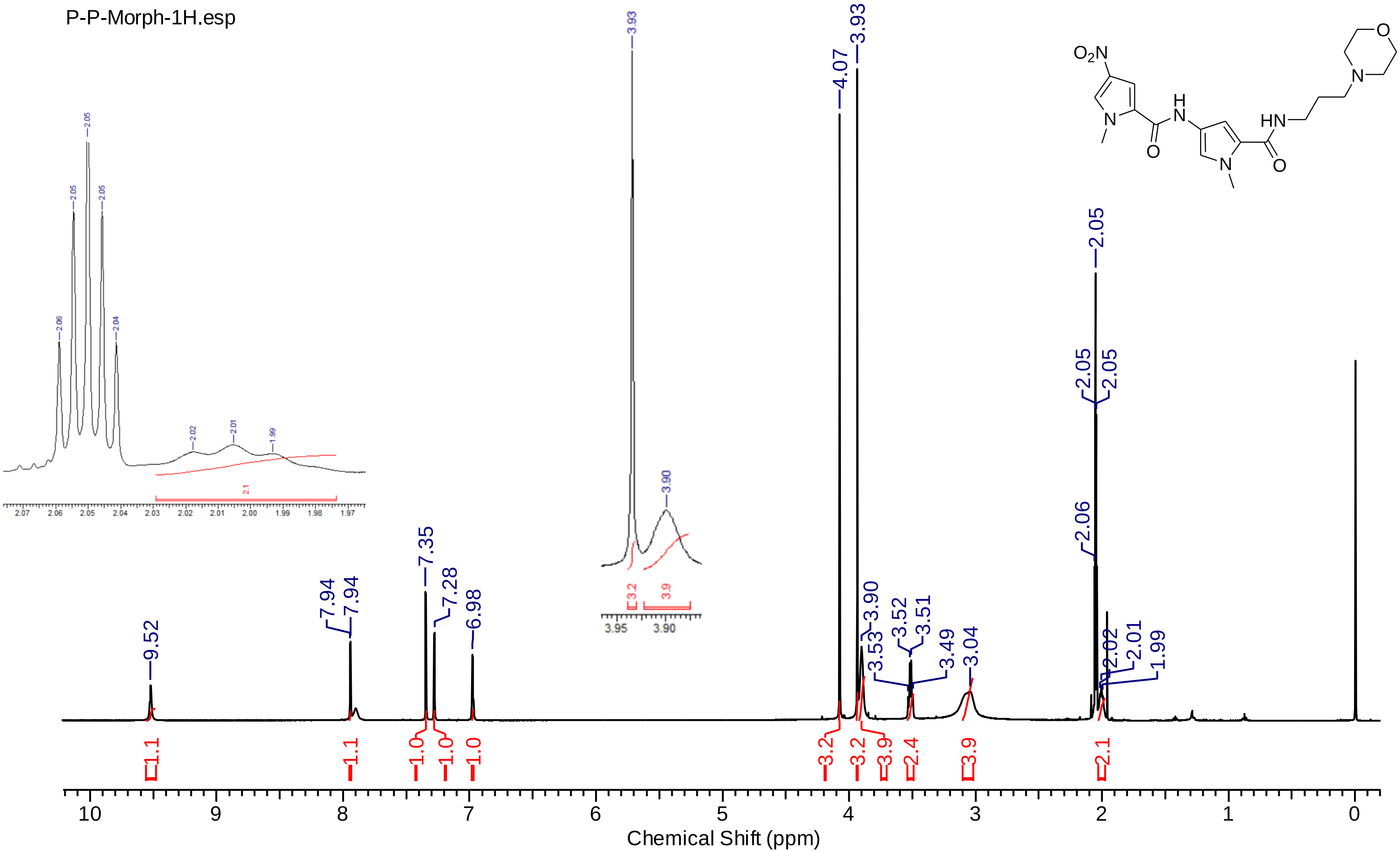

^{13}C NMR (ACETIC ACID-d_4): δ15.16, 17.54, 25.98, 26.77, 27.14, 33.12, 45.90, 94.87, 97.26, 112.50, 114.20, 117.02, 117.18, 137.53, 149.71; **(8)**

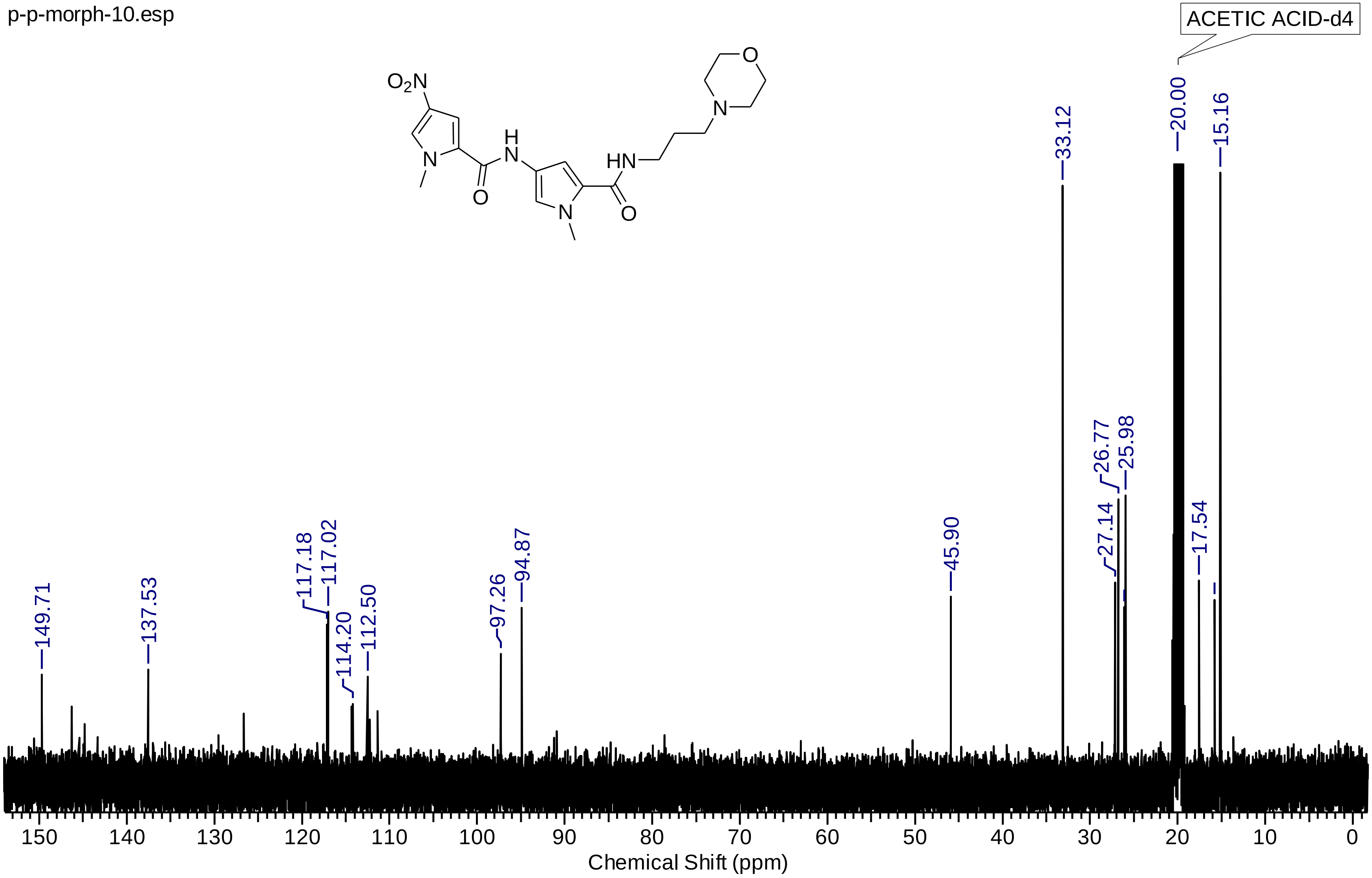

LC−MS (ESI): m/z calcd for $C_{19}H_{26}N_6O_5$, 418.2, found 419.12 [M + H]+. **(8)**

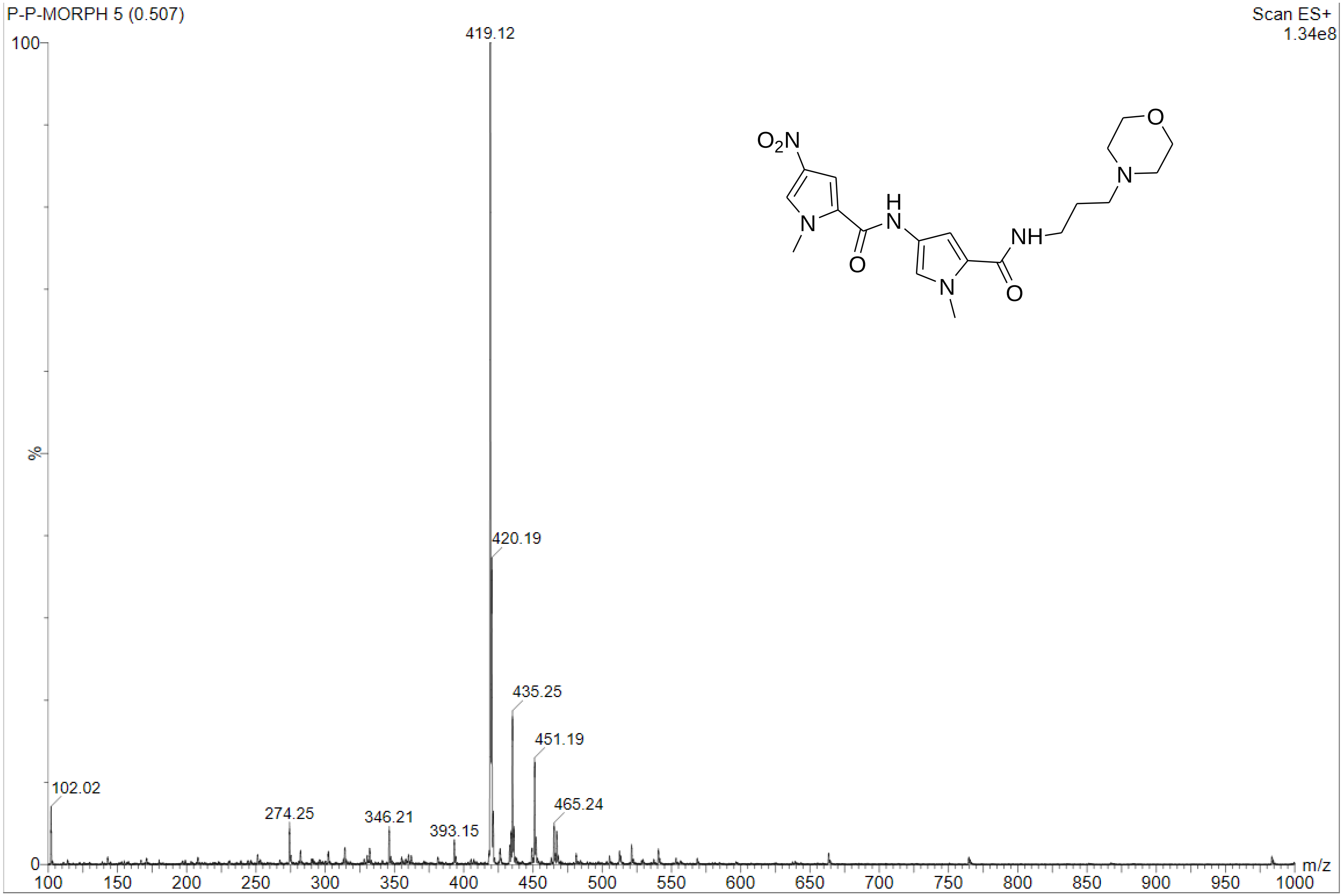

^{1}H NMR (ACETIC ACID-d_4) δ 1.32 (6H, d, CH_3), 2.12 (2H, m, CH_2), 3.02 (6H, s, NCH_3), 3.31 (2H, t, NCH_2), 3.49 (2H, q, CONH--CH_2), 3.97 (3H, s, NCH_3), 4.00 (3H, s, NCH_3), 4.11 (3H, s, NCH_3), 4.34 (1H, m, Ar—CH), 7.07 (1H, d, Ar—H), 7.09 (1H, d, Ar—H), 7.33 (1H, d, Ar—H), 7.35 (1H, d, Ar—H), 7.39 (1H, d, Ar—H), 7.49 (1H, d, Ar—H) ; **(MGB3)**

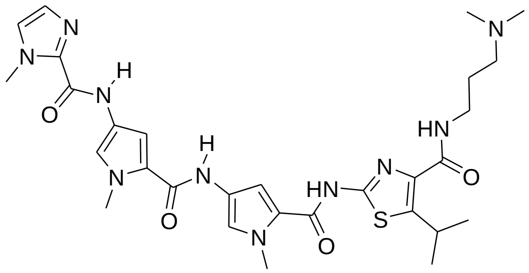

MGB-3-1H.esp

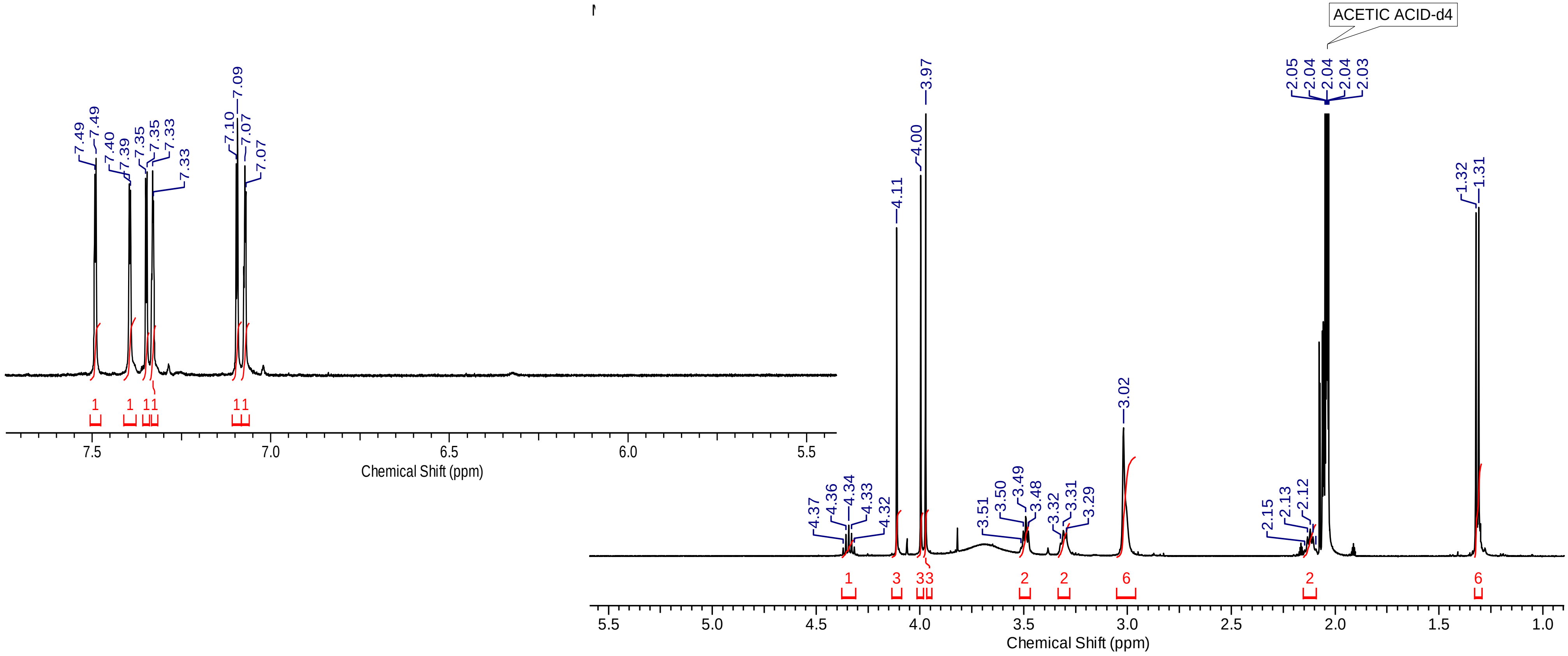

^{13}C NMR (ACETIC ACID-d_4): δ 15.30, 17.71, 26.28, 26.98, 27.34, 33.33, 46.07, 95.08, 97.47, 97.53, 109.76, 112.41, 114.43, 117.22, 126.70, 129.49, 137.79, 146.05, 149.83; **(MGB3)**

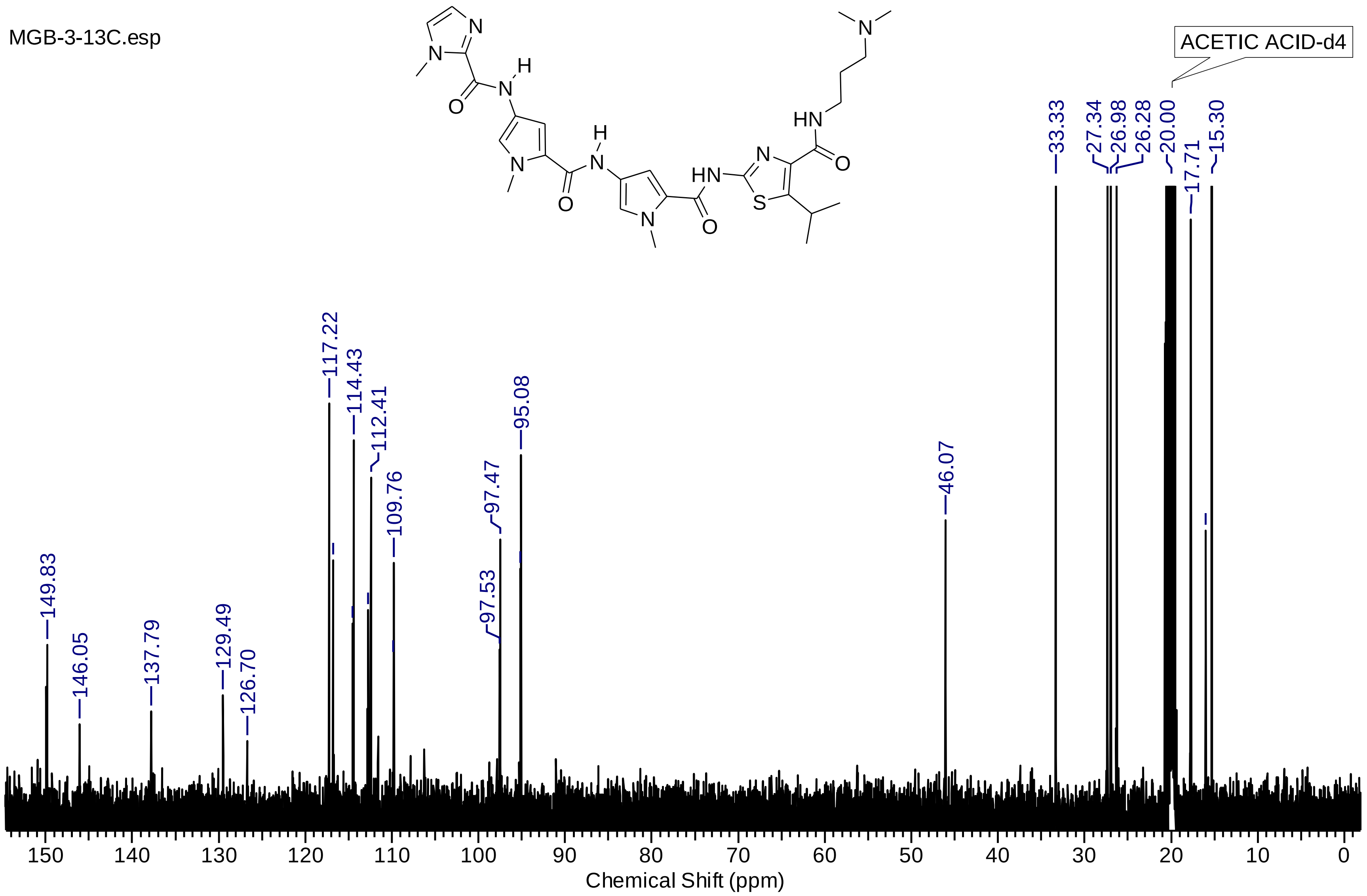

LC–MS (ESI): m/z calcd for $C_{29}H_{38}N_{10}O_4S$, 622.28, found 623.27 [M + H]+. **(MGB3)**

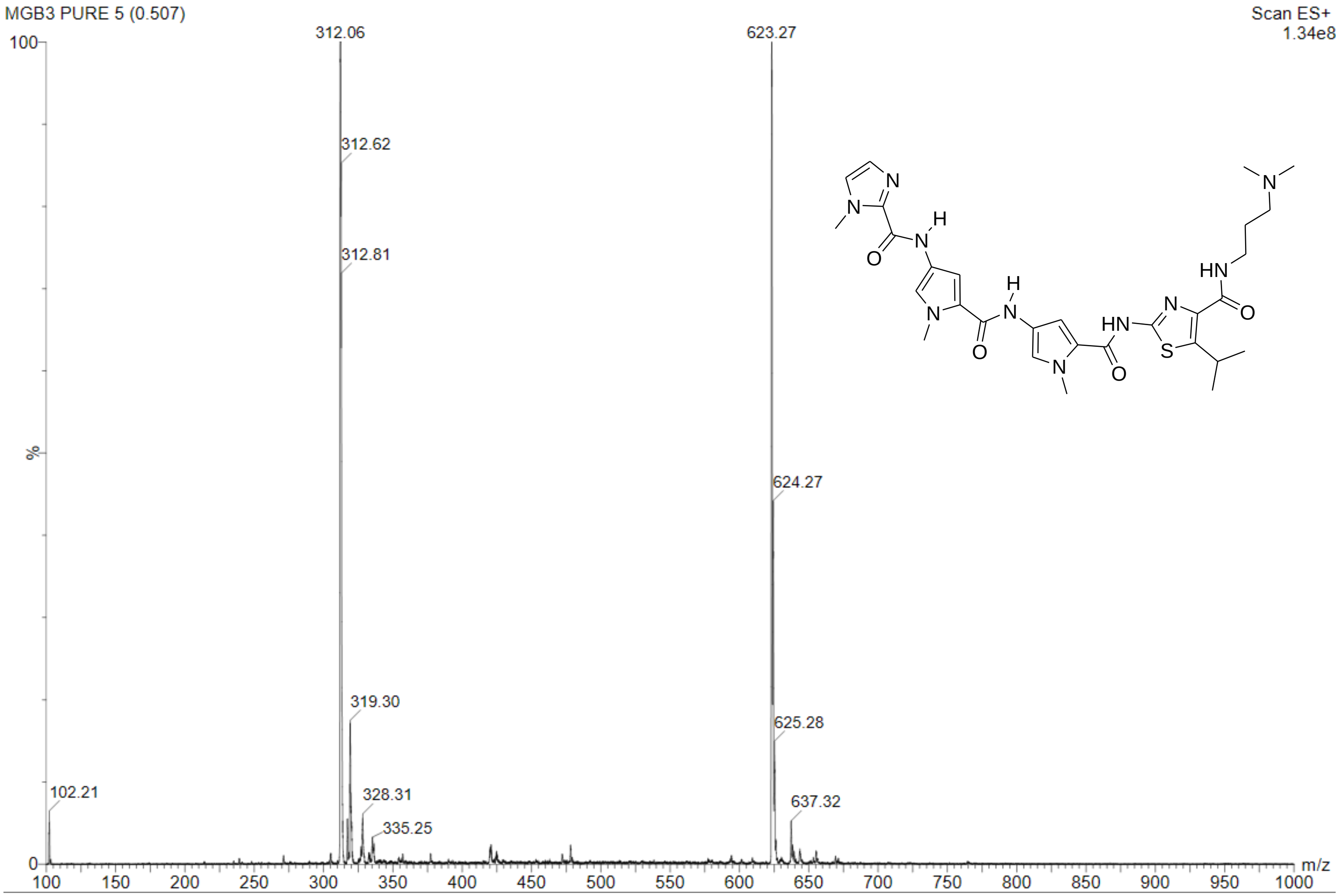

^{1}H NMR (ACETIC ACID-d_4) δ 1.32 (6H, d, CH_3), 2.12 (2H, m, CH_2), 3.03 (6H, s, NCH_3), 3.32 (2H, t, NCH_2), 3.49 (2H, q, CONH--CH_2), 3.97 (3H, s, NCH_3), 3.99 (3H, s, NCH_3), 4.34 (1H, m, Ar—CH), 7.15 (1H, d, Ar—H), 7.41 (1H, d, Ar—H), 7.43 (1H, d, Ar—H), 7.48 (1H, d, Ar—H), 8.32 (1H, d, Ar—H), 9.10 (1H, d, Ar—H), 9.52 (1H, s, CONH), 9.68 (1H, s, CONH) ; **(MGB4)**

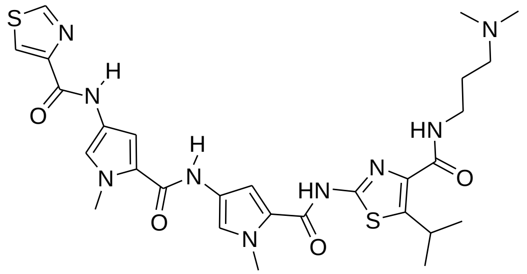

MGB-4-1H.esp

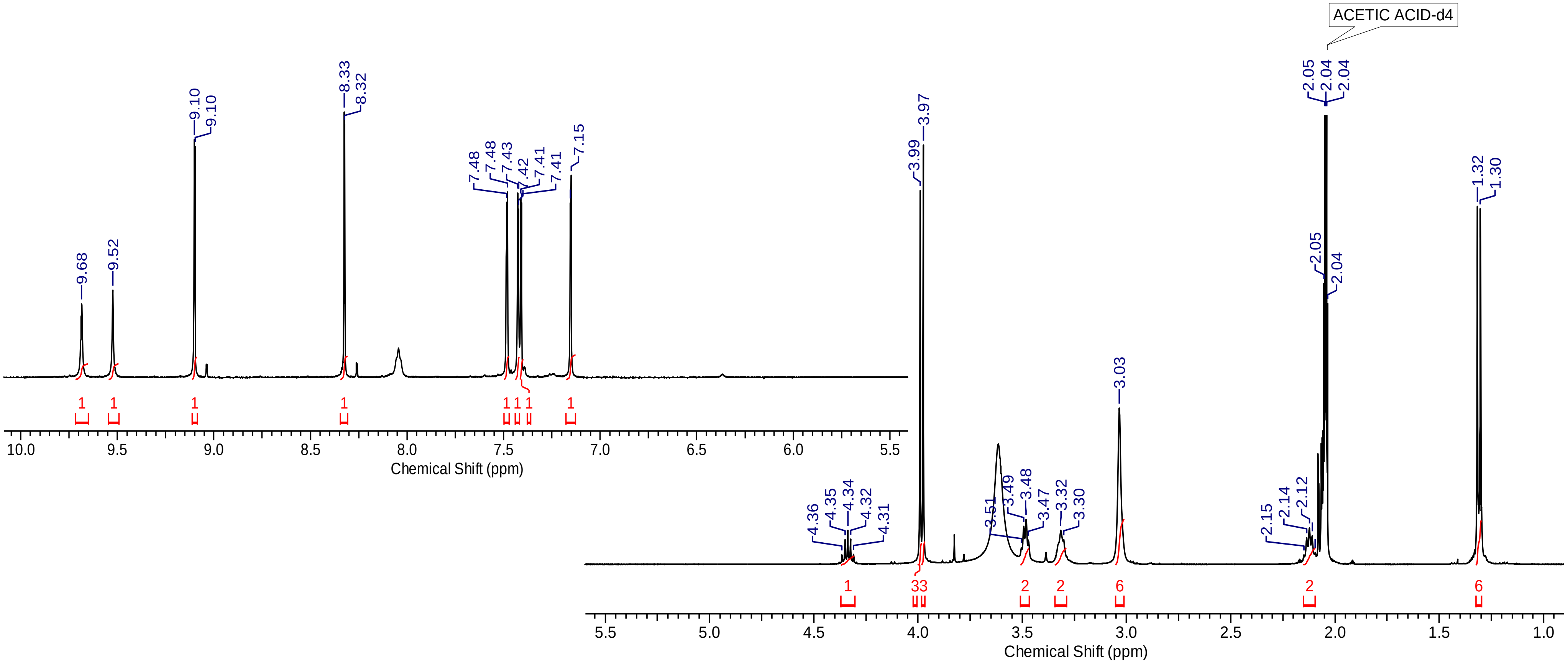

^{13}C NMR (ACETIC ACID-d_4): δ 15.14, 17.56, 26.04, 26.79, 27.15, 36.89, 45.93, 95.13, 97.42, 106.04, 108.35, 111.35, 112.65, 114.33, 126.47, 137.70, 142.24, 144.95, 148.55, 149.70, 149.78, 150.32, 154.38; **(MGB4)**

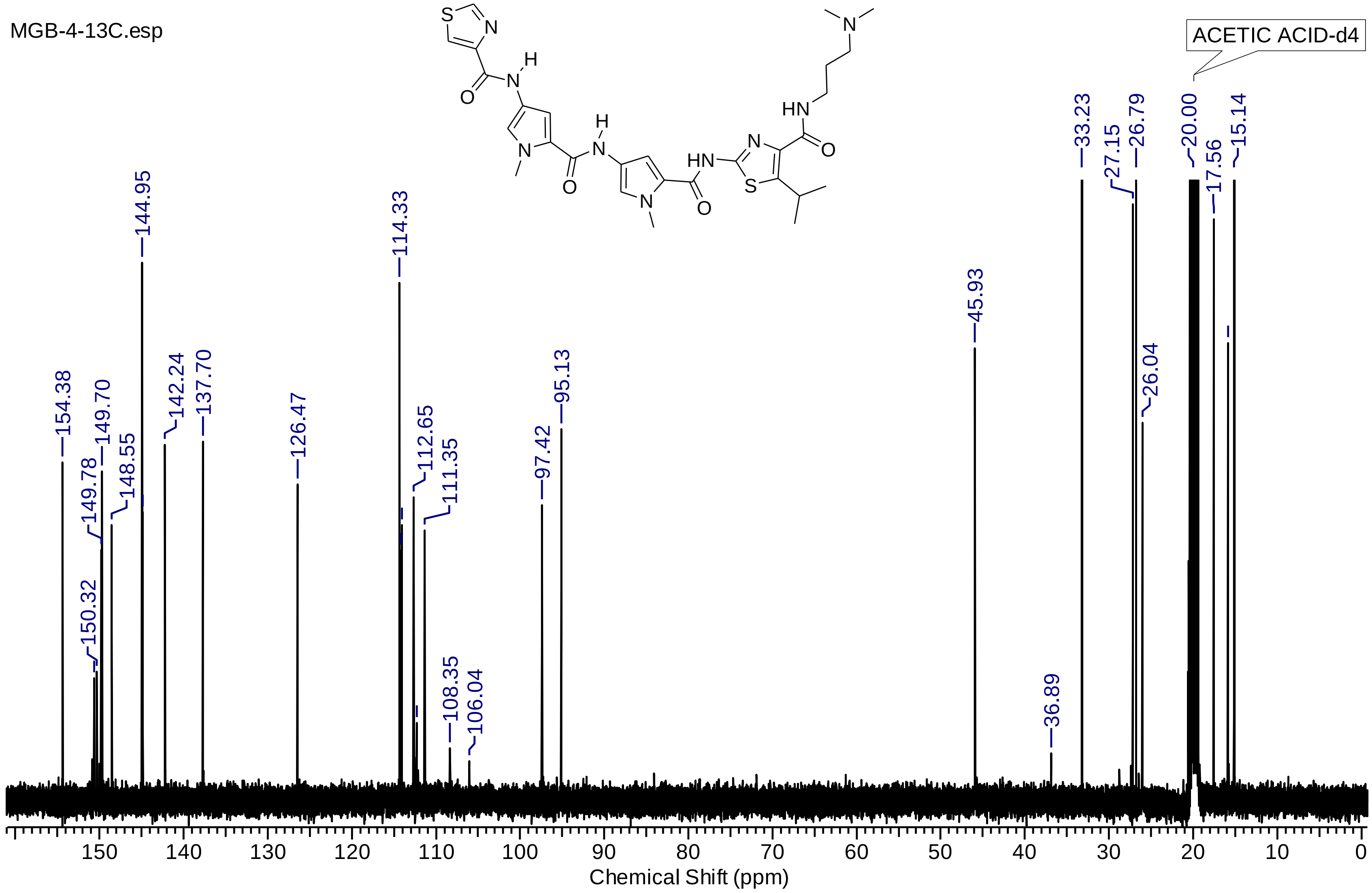

LC−MS (ESI): m/z calcd for $C_{28}H_{35}N_9O_4S_2$, 625.23, found 626.23 [M + H]+. **(MGB4)**

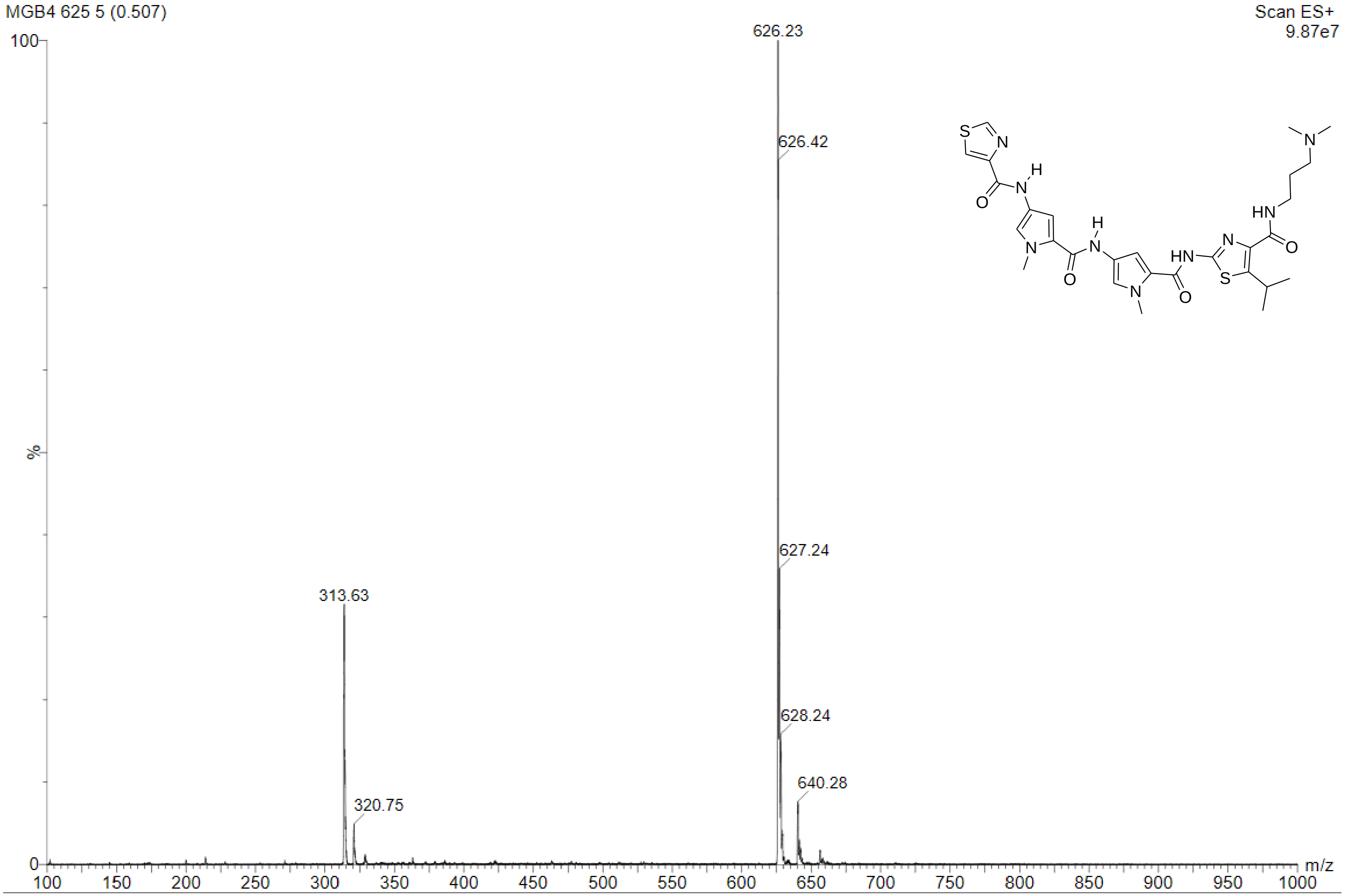

^{1}H NMR (ACETIC ACID-d$_4$) δ 2.11 (2H, m, CH$_2$), 3.04 (6H, s, NCH$_3$), 3.33 (2H, t, NCH$_2$), 3.36 (6H, s, NCH$_3$), 3.49 (2H, q, CONH--CH$_2$), 3.90 (3H, s, NCH$_3$), 3.93 (3H, s, NCH$_3$), 3.96 (3H, s, NCH$_3$), 4.13 (1H, s, NH) 6.93 (1H, d, Ar—H), 6.97 (1H, d, Ar—H), 7.04 (1H, d, Ar—H), 7.23 (1H, d, Ar—H), 7.26 (1H, d, Ar—H), 7.34 (1H, d, Ar—H), 7.83 (1H, s, CONH), 9.26 (1H, s, CONH), 9.34 (1H, s, CONH) ; **(MGB5)**

MGB-5-1H.esp

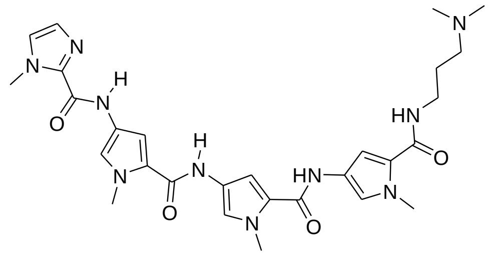

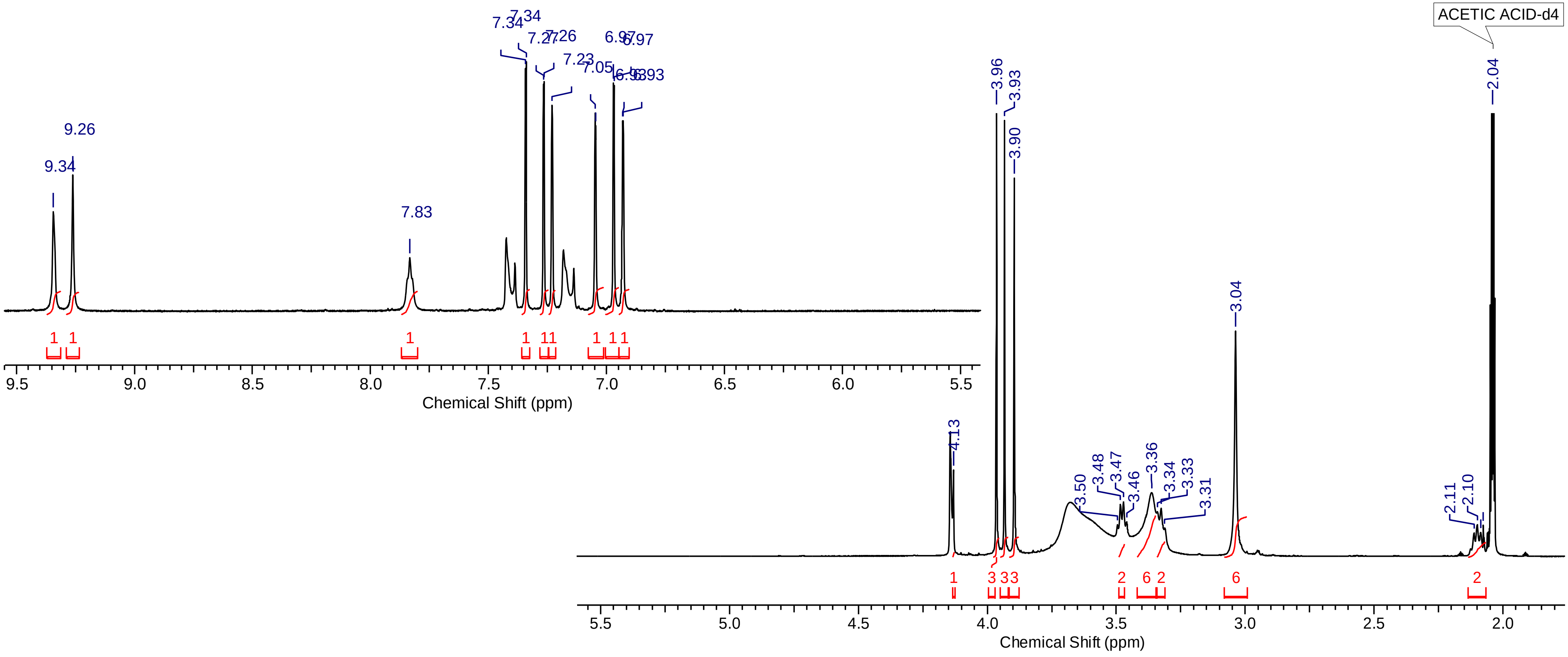

^{13}C NMR (ACETIC ACID-d_4): δ 16.21, 20.81, 20.90, 26.30, 26.54, 26.92, 27.01, 27.10, 33.40, 46.02, 95.02, 95.38, 109.79, 112.63, 113.78, 113.95, 114.32, 114.77, 116.53, 117.42, 145.87, 149.78, 149.92, 154.01; **(MGB5)**

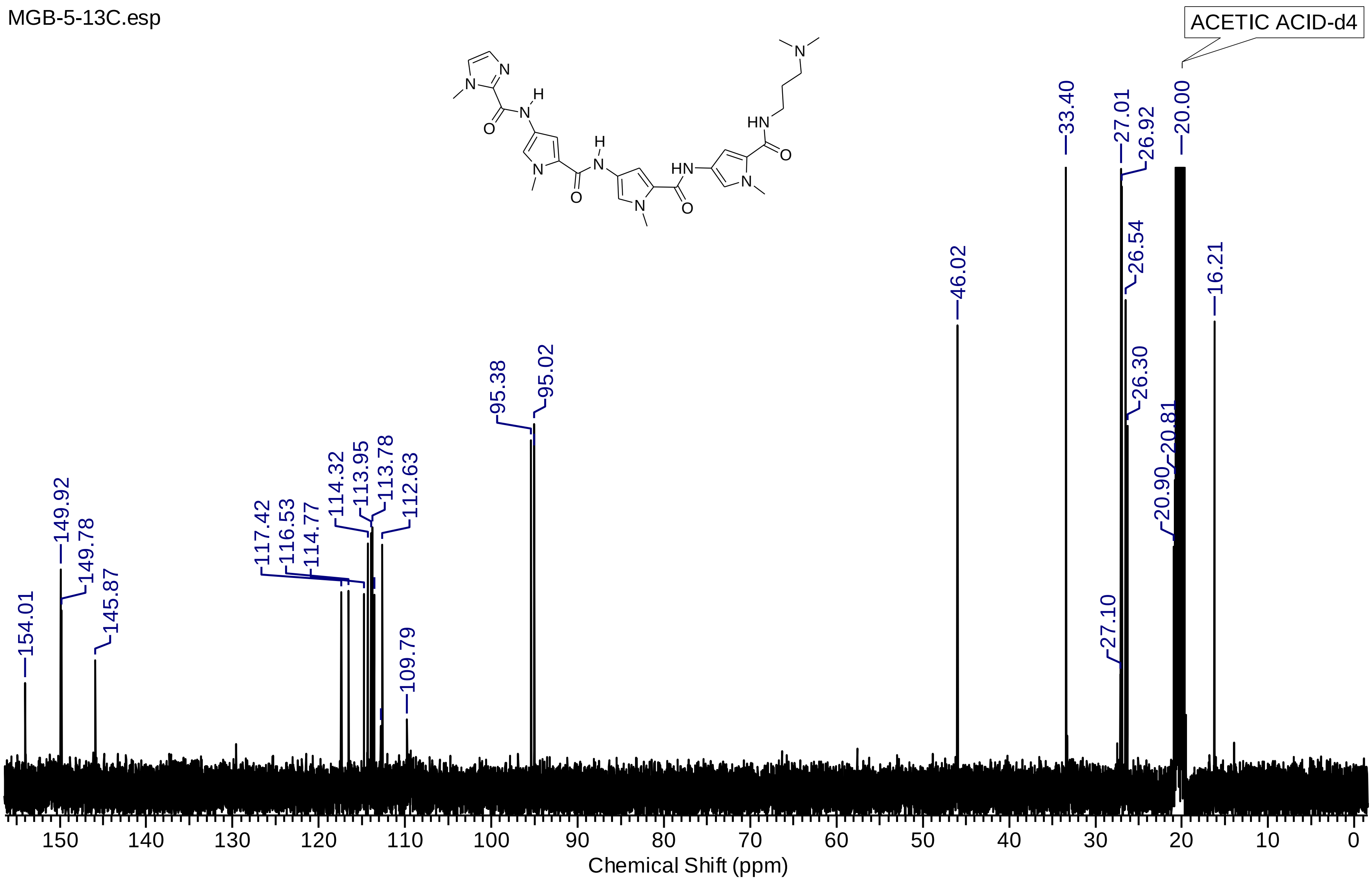

LC−MS (ESI): m/z calcd for $C_{27}H_{38}N_{10}O_4$, 576.29, found 577.33 [M + H]+. **(MGB5)**

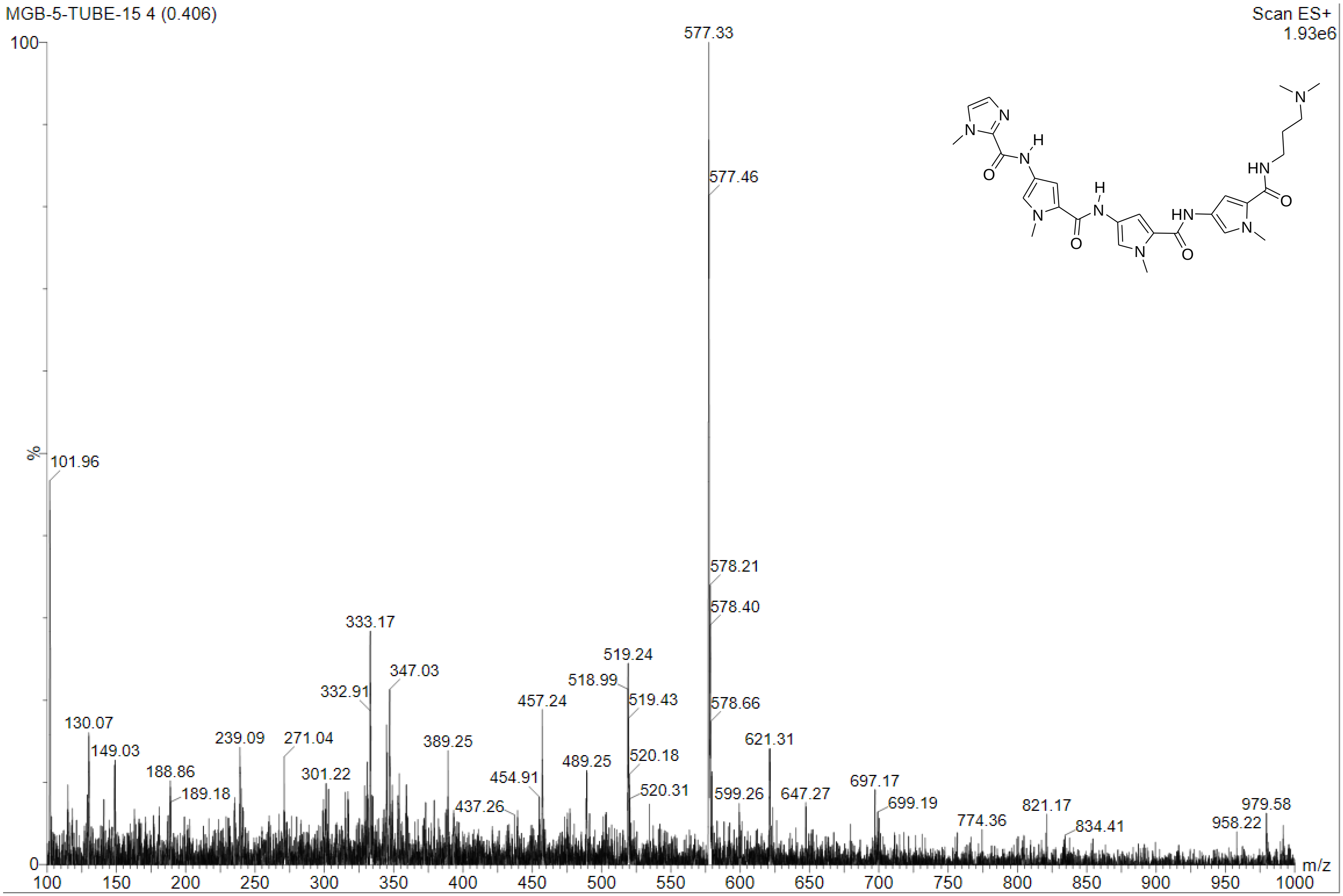

^{1}H NMR (ACETIC ACID-d_4) δ 2.08 (2H, m, CH_2), 2.99 (6H, s, NCH_3), 3.30 (2H, t, NCH_2), 3.45 (2H, m, CONH--CH_2), 3.89 (3H, s, NCH_3), 3.94 (3H, s, NCH_3), 3.97 (3H, s, NCH_3), 6.89 (1H, s, Ar—H), 6.97 (1H, s, Ar—H), 7.09 (1H, d, Ar—H), 7.15 (1H, d, Ar—H), 7.23 (1H, s, Ar—H), 7.27 (1H, d, Ar—H), 7.39 (1H, s, Ar—H), 7.41 (1H, s, Ar—H), 8.32 (1H, s, CONH), 8.34 (1H, s, CONH), 9.10 (1H, s, CONH), 9.11 (1H, s, CONH) ; (**MGB6**)

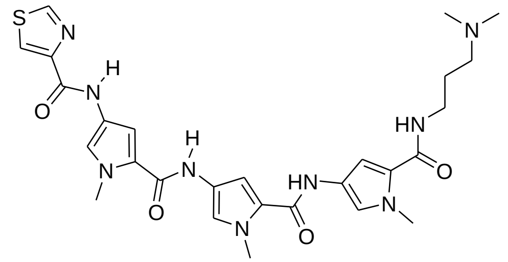

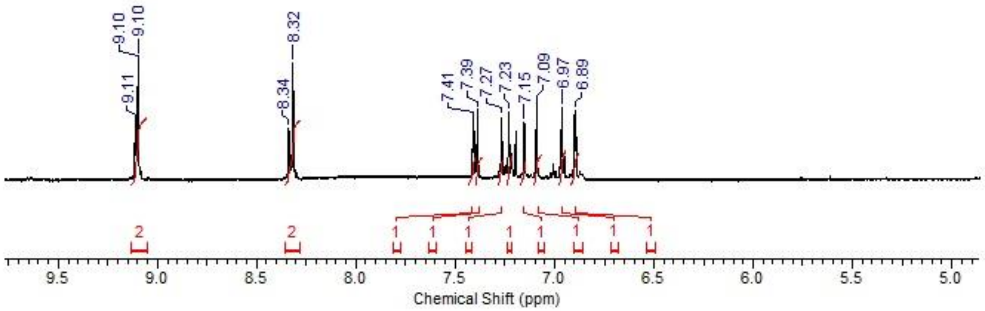

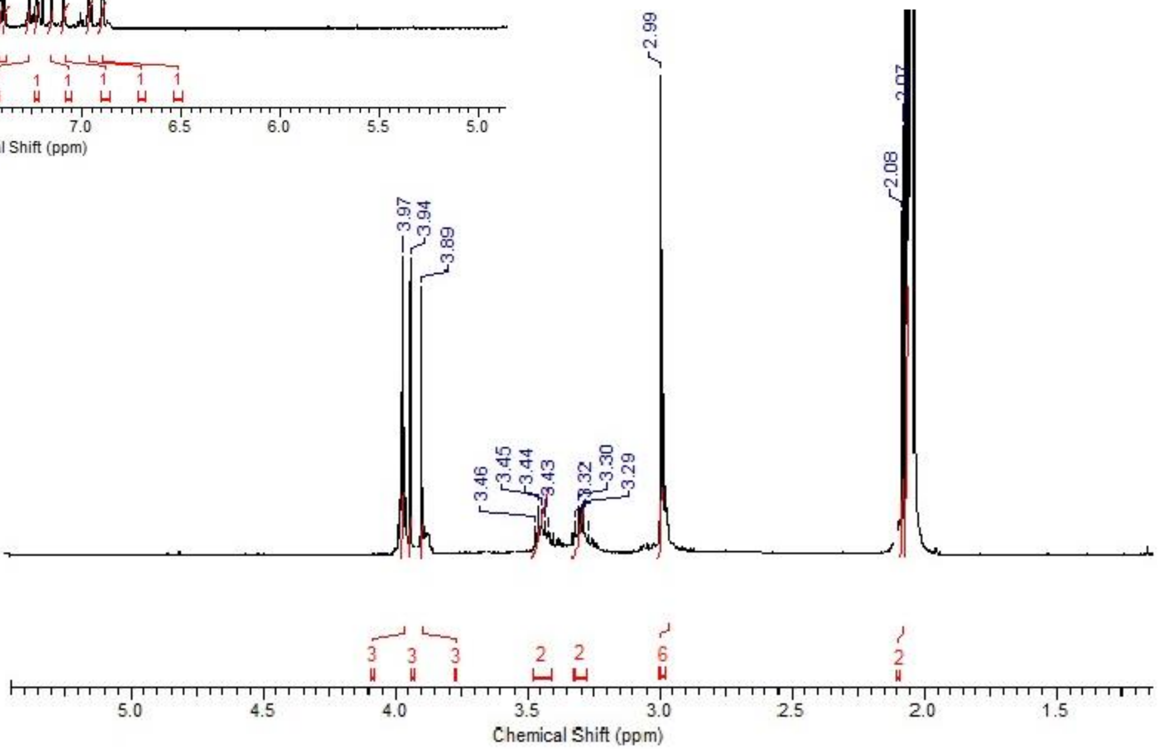

^{13}C NMR (CHLOROFORM-d): δ 25.19, 36.62, 36.78, 36.97, 34.22, 104.10, 104.37, 104.50, 111.11, 118.46, 119.69, 121.14, 122.06, 123.43, 123.52, 124.54, 125.10, 127.76, 132.23, 143.48, 150.88, 153.11, 158.33, 159.17, 162.72; (**MGB6**)

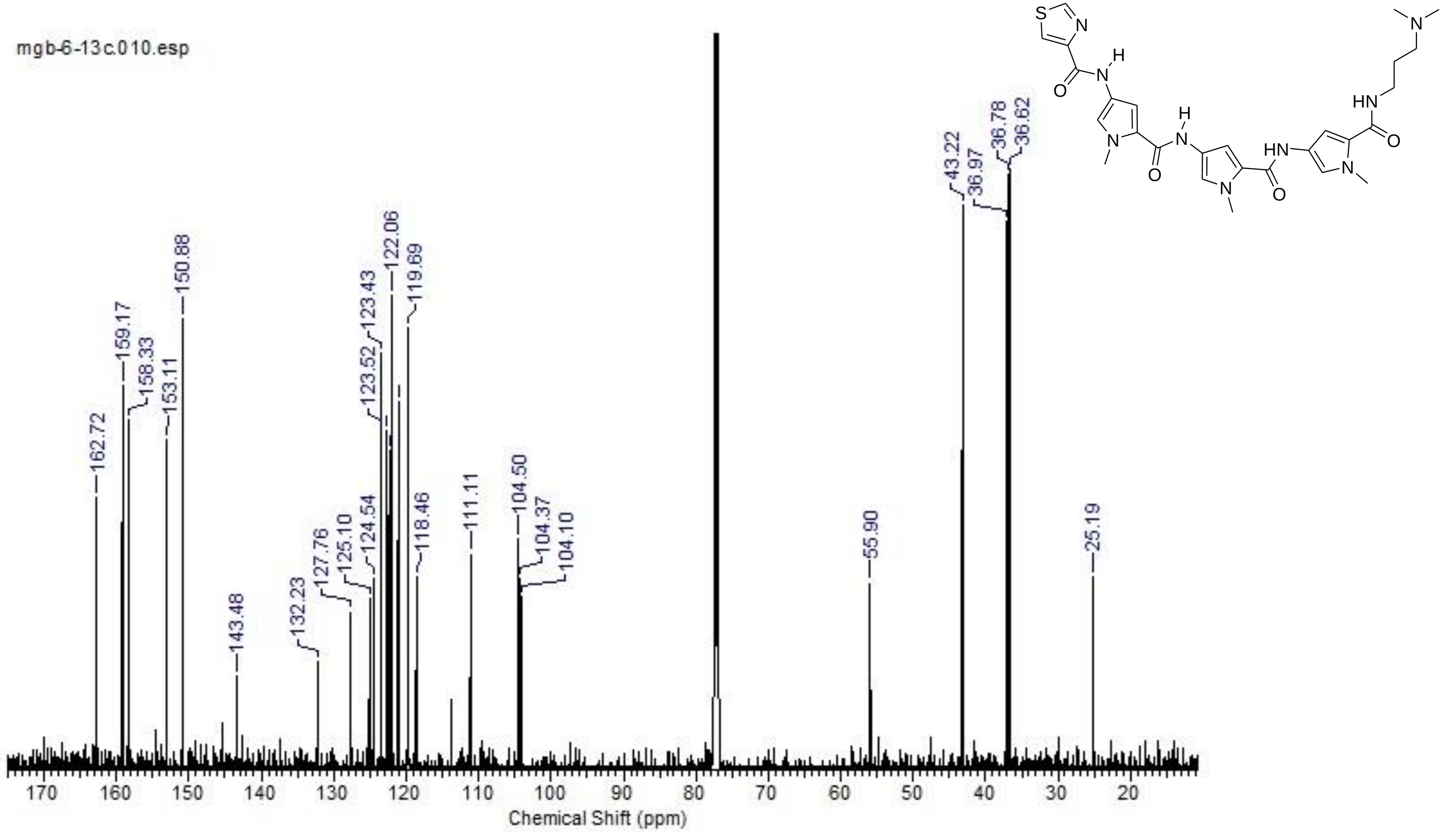

LC−MS (ESI): m/z calcd for $C_{27}H_{33}N_9O_4S$, 579.24, found 580.32 [M + H]+. (**MGB6**)

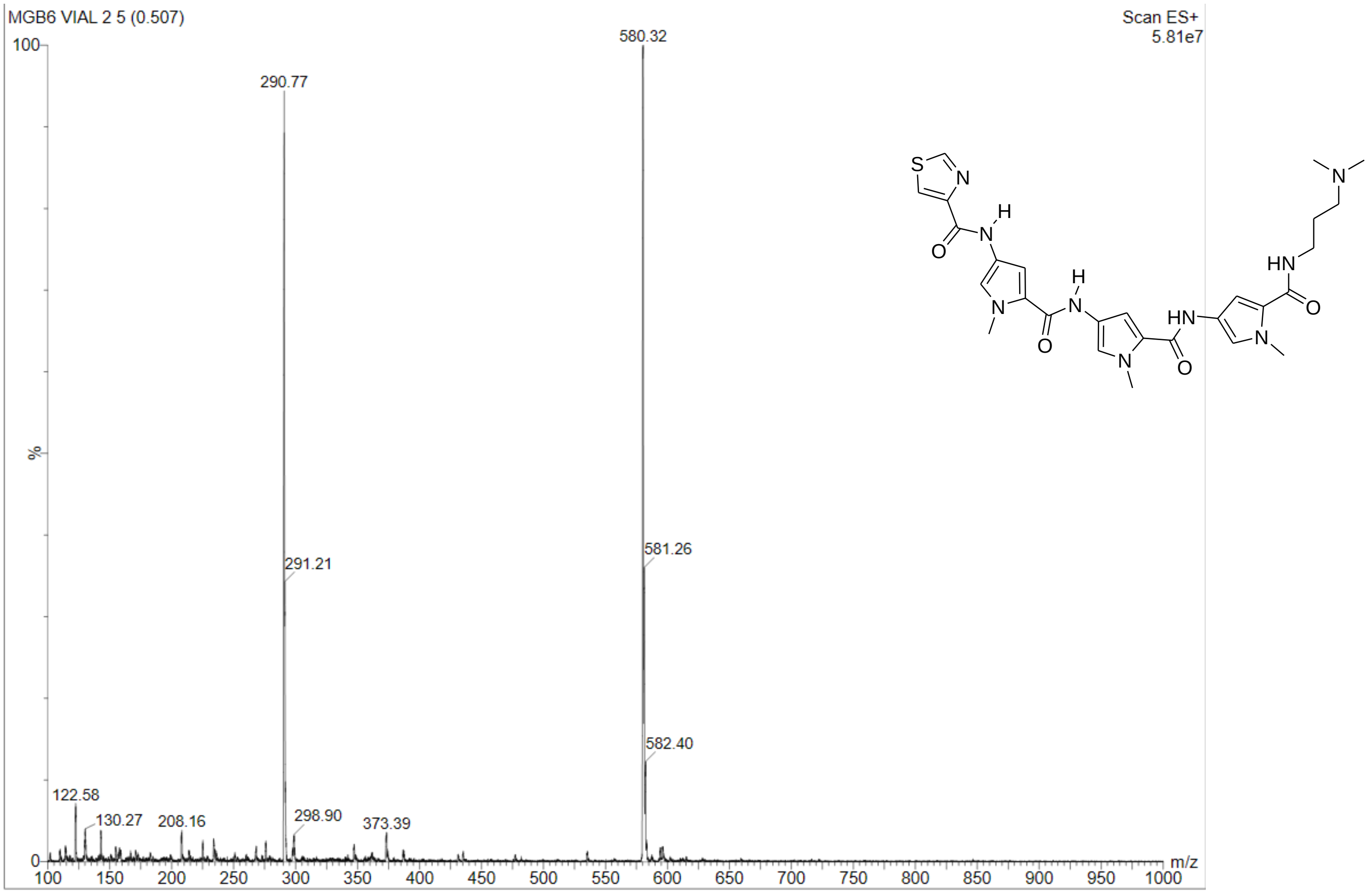

^{1}H NMR (ACETIC ACID-d_4) δ 2.18 (2H, m, CH_2), 3.32 (2H, t, NCH_2), 3.43 (2H, m, NCH_2), 3.59 (2H, q, CONH--CH_2), 3.75 (4H, d, OCH_2), 3.93 (3H, s, NCH_3), 3.98 (3H, s, NCH_3), 6.95 (1H, d, Ar—H), 7.04 (1H, d, Ar—H), 7.30 (1H, d, Ar—H), 7.44 (1H, d, Ar—H), 7.49 (1H, s, Ar—H), 7.50 (1H, d, Ar—H), 7.81 (1H, t, Ar—H), 8.00 (1H, d, Ar—H), 8.08 (1H, t, Ar—H) , 8.98 (1H, s, CONH), 9.26 (1H, s, CONH), 10.57 (1H, s, CONH) ; **(MGB16)**

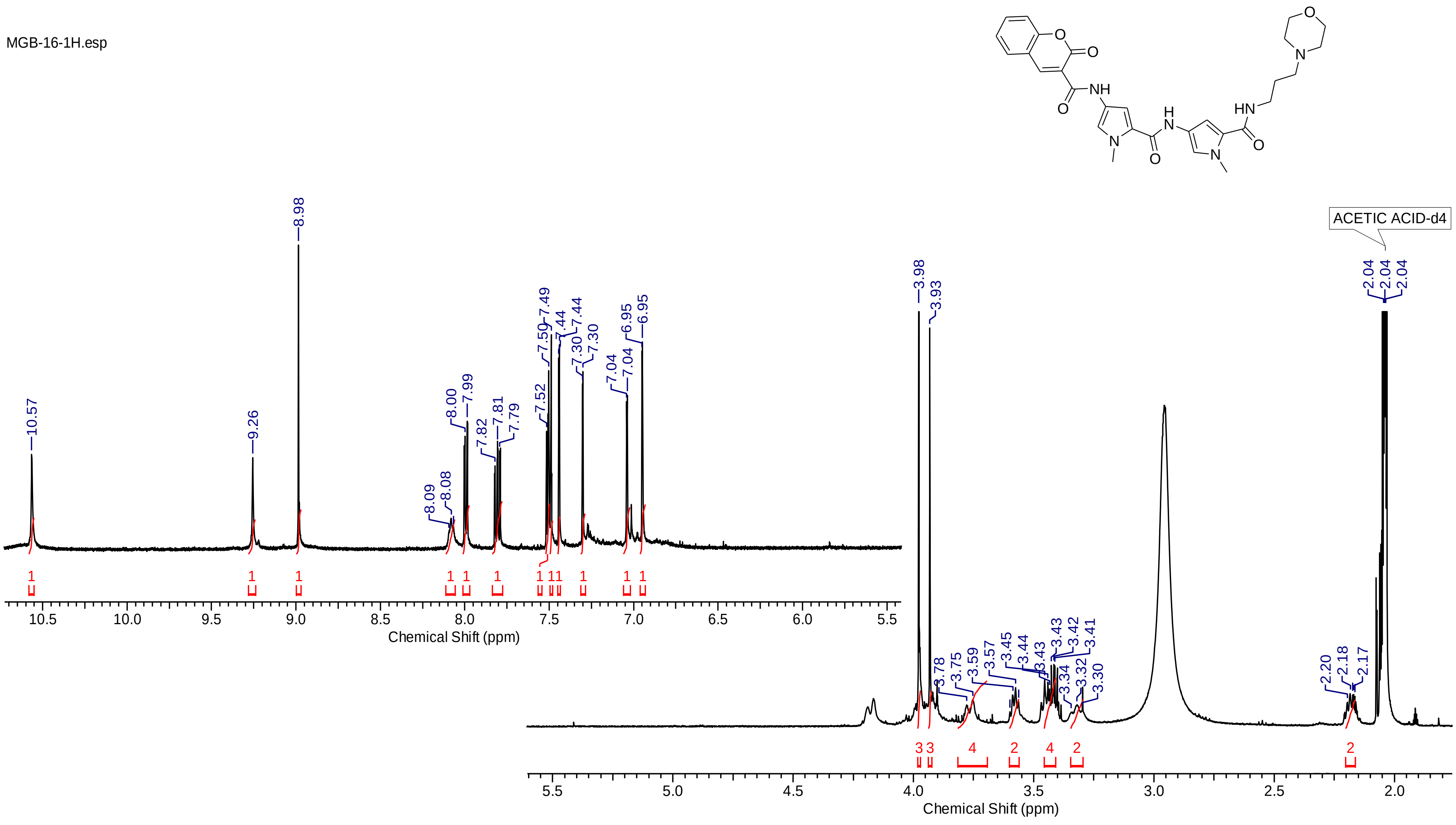

^{13}C NMR (DMSO-d_6): δ 8.48, 30.54, 35.89, 36.11, 45.61, 51.05, 54.05, 63.37, 104.27, 116.08, 118.41, 119.24, 122.44, 123.08, 125.15, 130.10, 134.02, 146.86, 153.66, 175.95, 158.04, 160.58, 161.49; **(MGB16)**

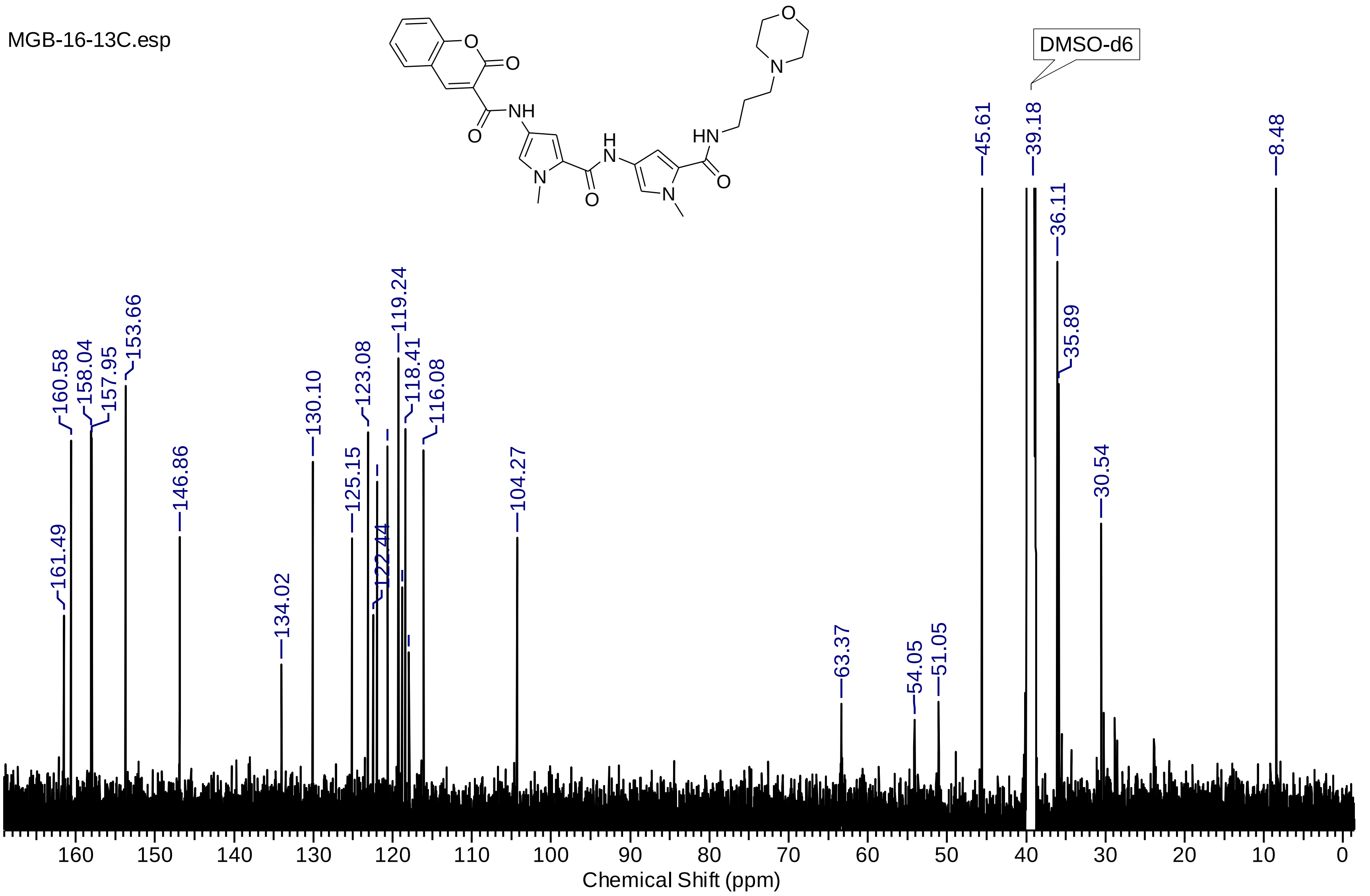

LC−MS (ESI): m/z calcd for $C_{29}H_{32}N_6O_6$, 560.24, found 561.27 [M + H]+. **(MGB16)**

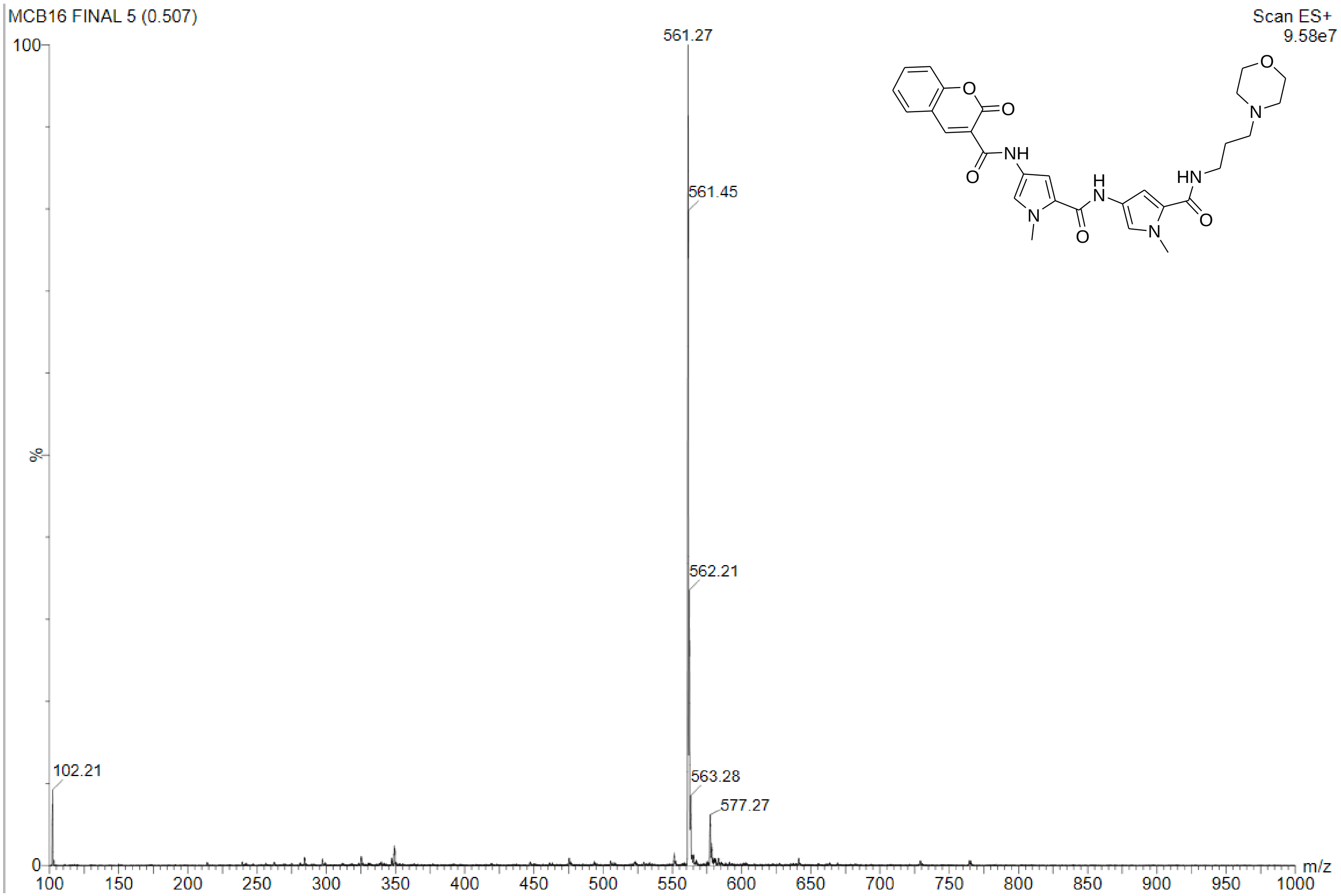

MGB-20-1H.esp

^{1}H NMR (ACETIC ACID-d$_4$) δ, 2.14 (2H, m, CH_2), 2.76 (2H, t, SCH_2), 3.22 (4H, t, NCH_2), 3.35 (2H, t, NCH_2), 3.39 (2H, t, NHCO--CH_2), 3.49 (2H, q, CONH--CH_2), 3.66 (4H, t, OCH_2), 3.90 (3H, s, NCH_3), 3.90 (3H, s, NCH_3), 6.80 (1H, d, Ar—H), 6.91 (1H, s, Ar—H), 7.13 (1H, d, Ar—H), 7.26 (1H, s, Ar—H), 7.46 (1H, m, Ar—H), 7.48 (1H, d, Ar—H), 7.51 (1H, t, Ar—H), 7.86 (4H, q, Ar—H), 9.11 (1H, s, CONH), 9.20 (1H, s, CONH) ; **(MGB20)**

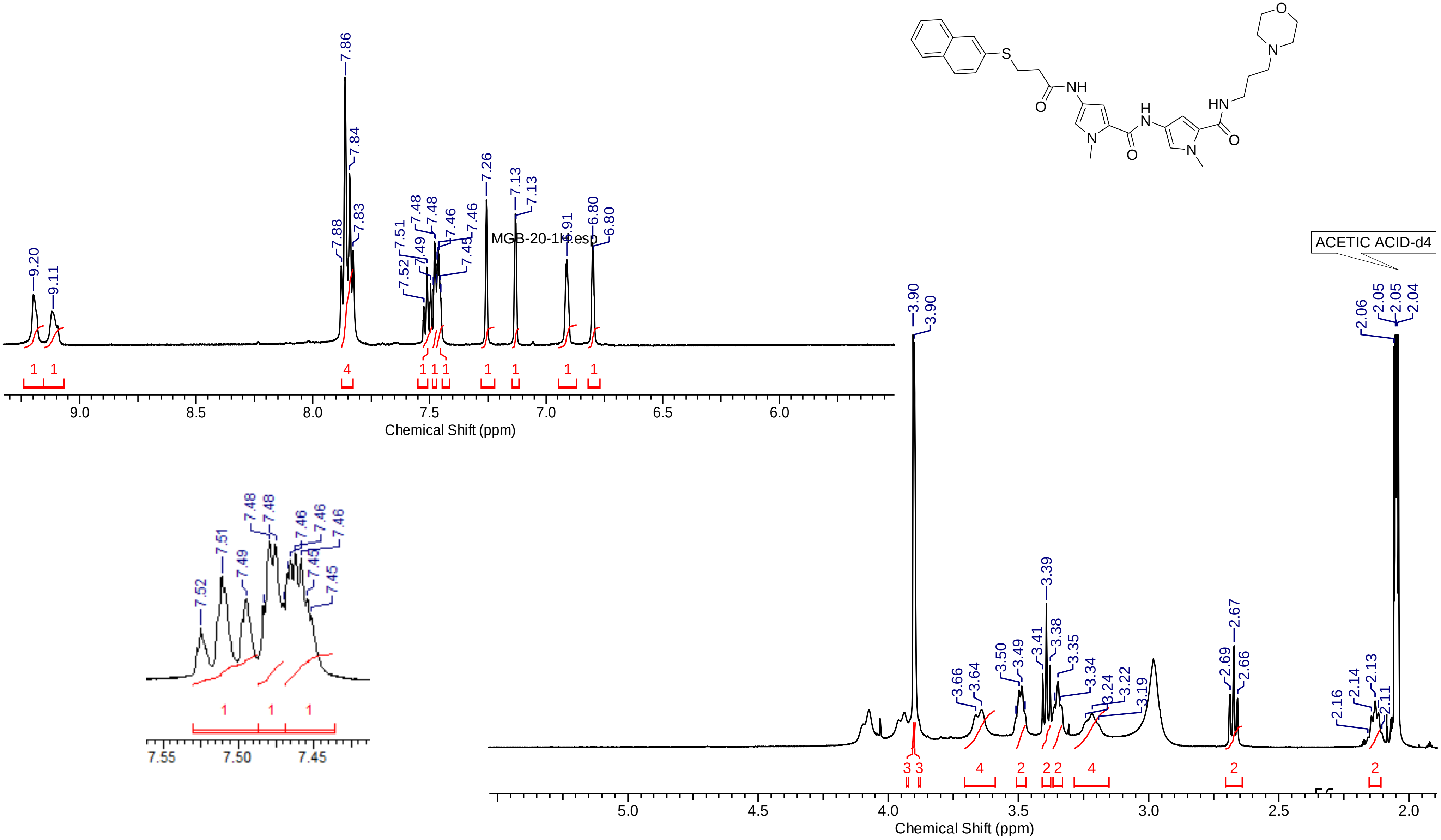

^{13}C NMR (DMSO-d_6): δ 8.48, 30.54, 35.89, 36.11, 45.61, 51.05, 54.05, 63.37, 104.27, 116.08, 118.41, 119.24, 120.62, 123.08, 125.15, 130.10, 134.02, 146.86, 153.66, 175.95, 158.04, 160.58, 161.49; **(MGB20)**

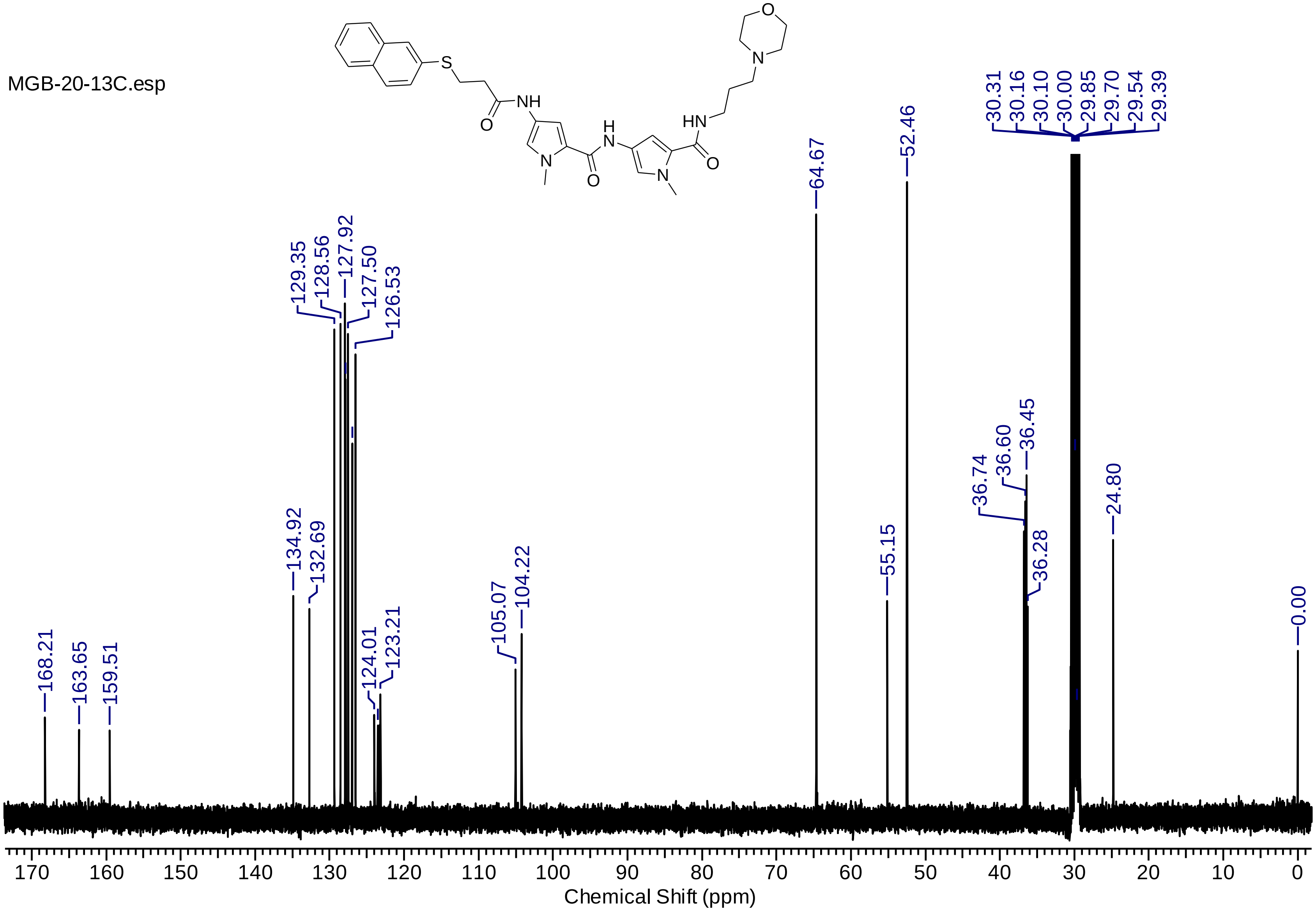

LC−MS (ESI): m/z calcd for $C_{32}H_{38}N_6O_4S$, 602.27, found 603.29 [M + H]+. **(MGB20)**

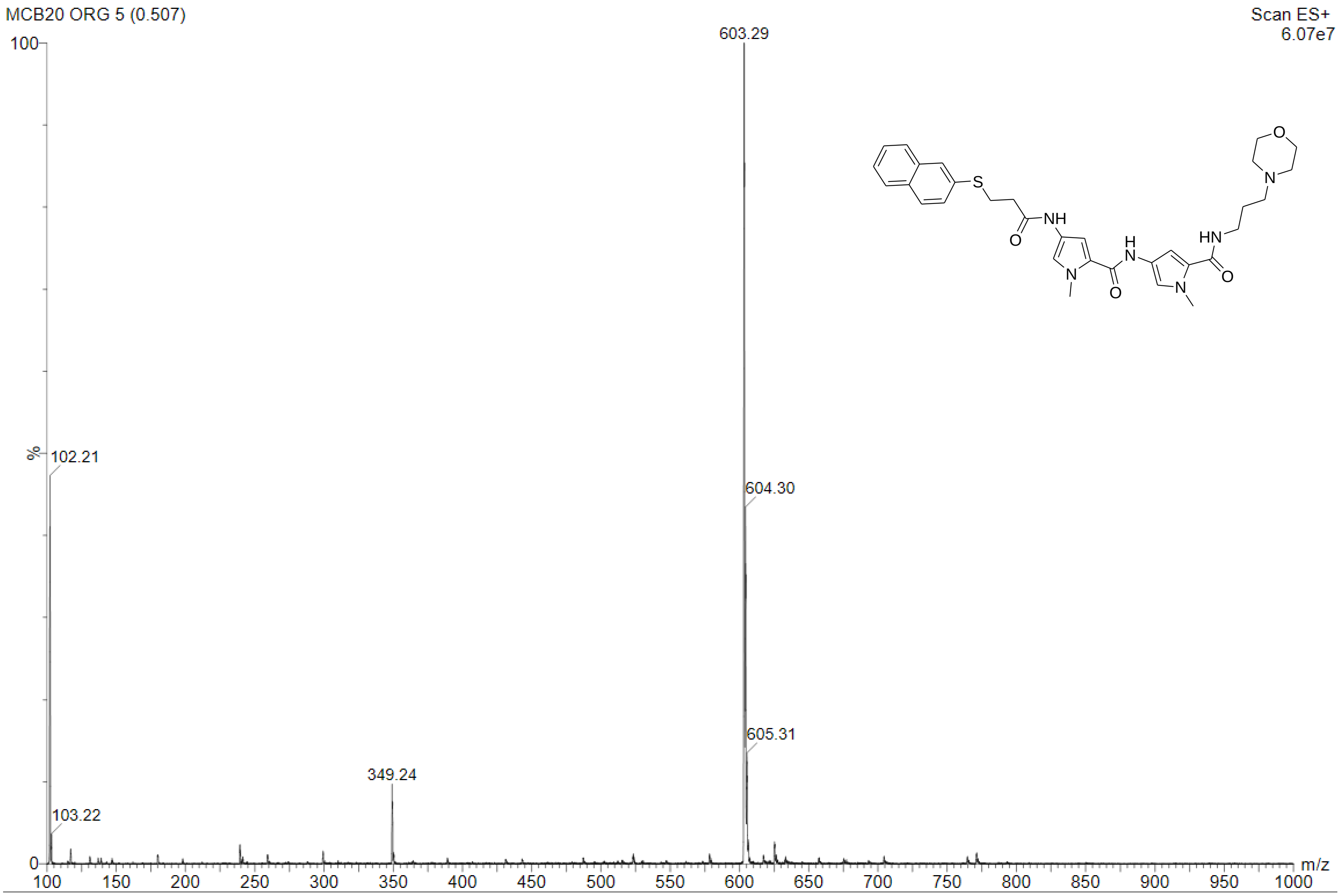

^{1}H NMR (ACETIC ACID-d$_4$) δ 2.11 (2H, m, CH$_2$), 2.28 (3H, s, CH$_3$), 2.54 (2H, t, SCH$_2$), 2.71 (2H, t, NHCO—CH$_2$), 3.33 (2H, s, NCH$_2$), 3.49 (2H, q, CONH--CH$_2$), 3.72 (2H, s, SCH$_2$—Ar), 3.89 (3H, s, NCH$_3$), 3.90 (3H, s, NCH$_3$), 6.81 (1H, s, Ar—H), 6.91 (1H, s, Ar—H), 7.10 (1H, s, Ar—H), 7.12 (1H, s, Ar—H), 7.21 (2H, d, Ar—H), 7.24 (1H, s, Ar--H), 7.84 (1H, s, CONH), 9.04 (1H, d, CONH), 9.19 (1H, d, CONH) ; **(MGB22)**

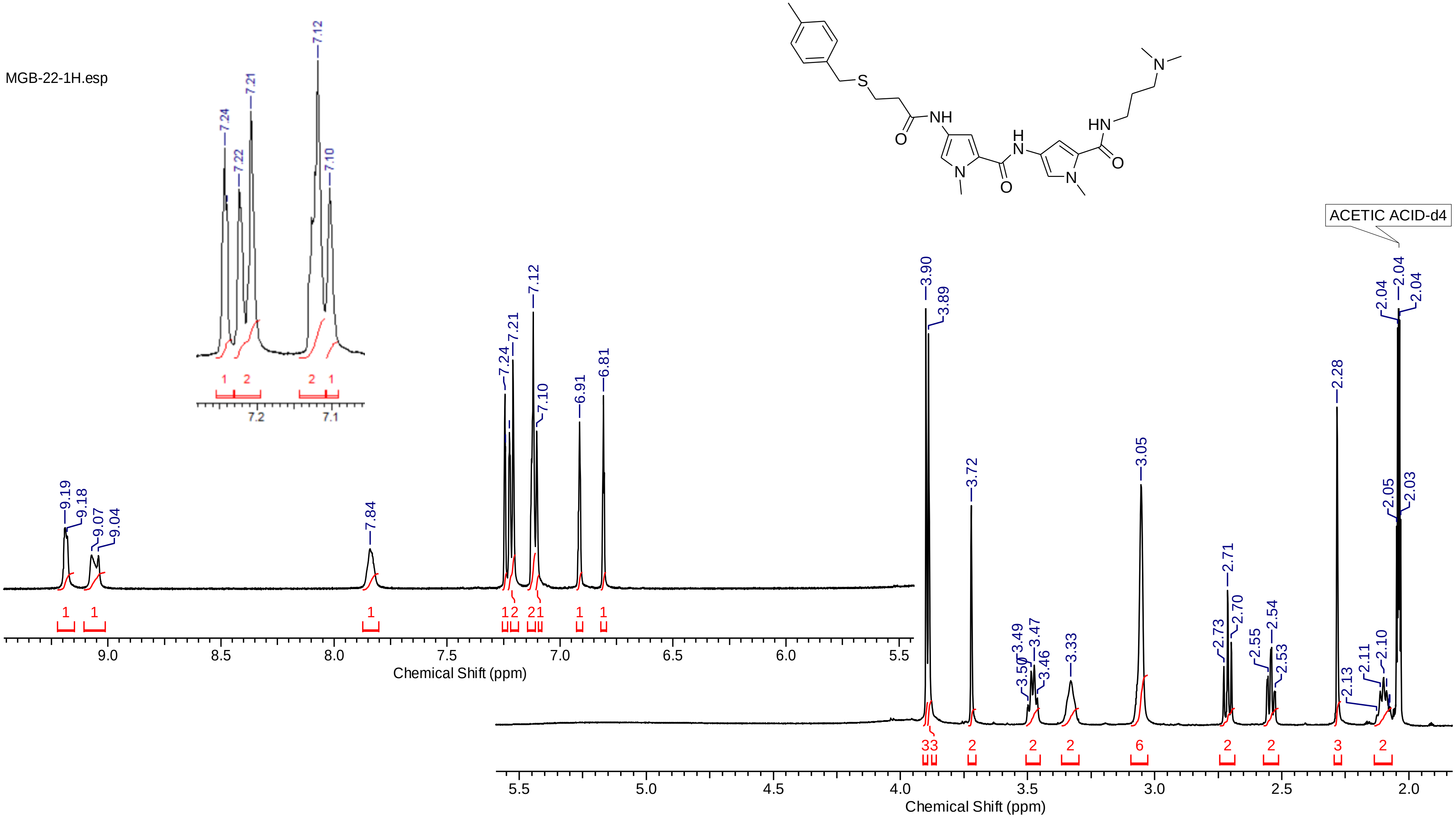

^{13}C NMR (ACETIC ACID-d_4): δ 11.22, 16.12, 17.83, 26.04, 26.32, 27.17, 33.29, 45.84, 94.44, 95.30, 113.32, 113.43, 114.14, 119.87, 119.99, 126.77, 127.27, 149.68, 154.01, 158.79; **(MGB22)**

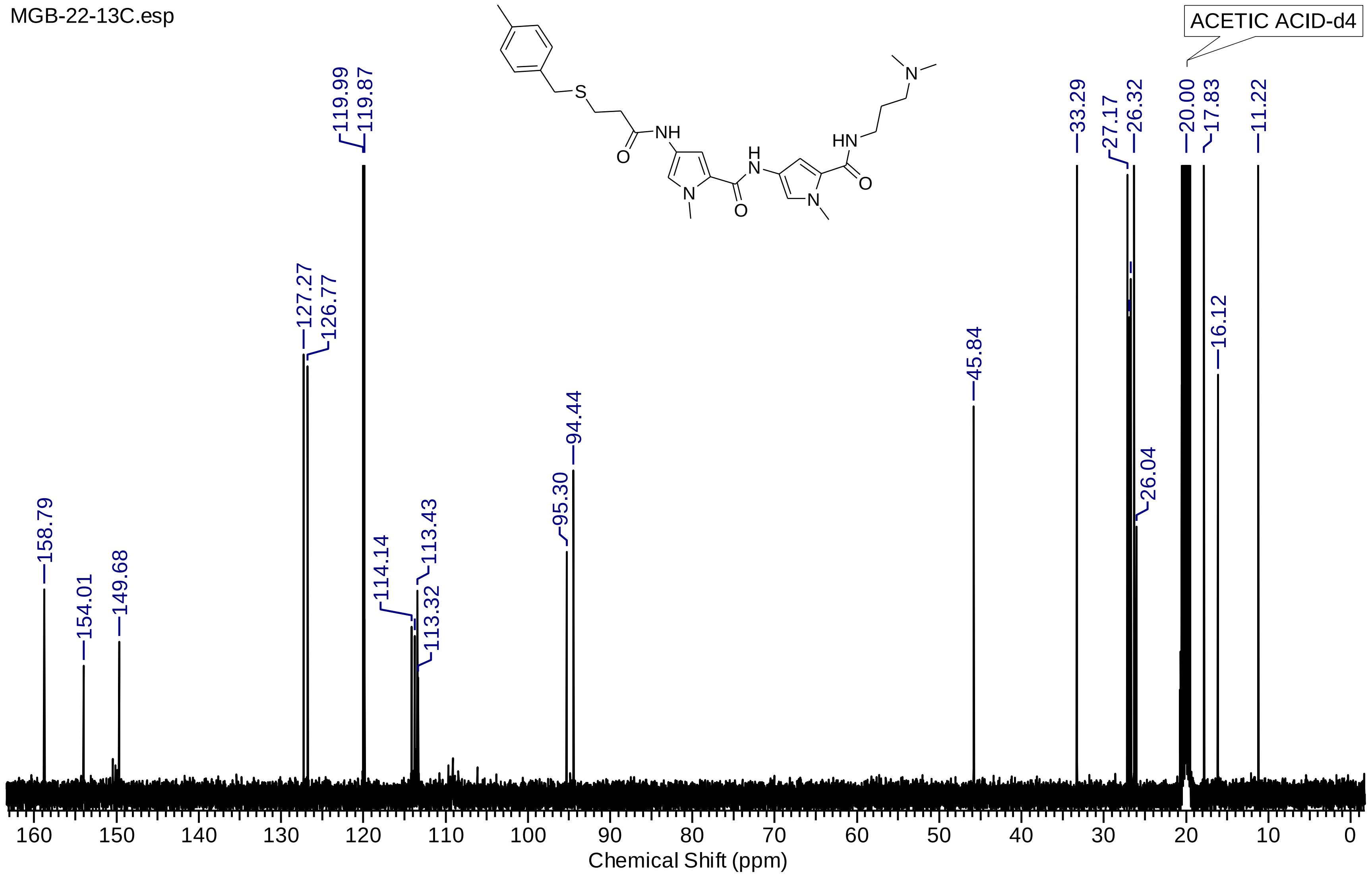

LC−MS (ESI): m/z calcd for $C_{28}H_{38}N_6O_3S$, 538.27, found 539.42 [M + H]+. **(MGB22)**

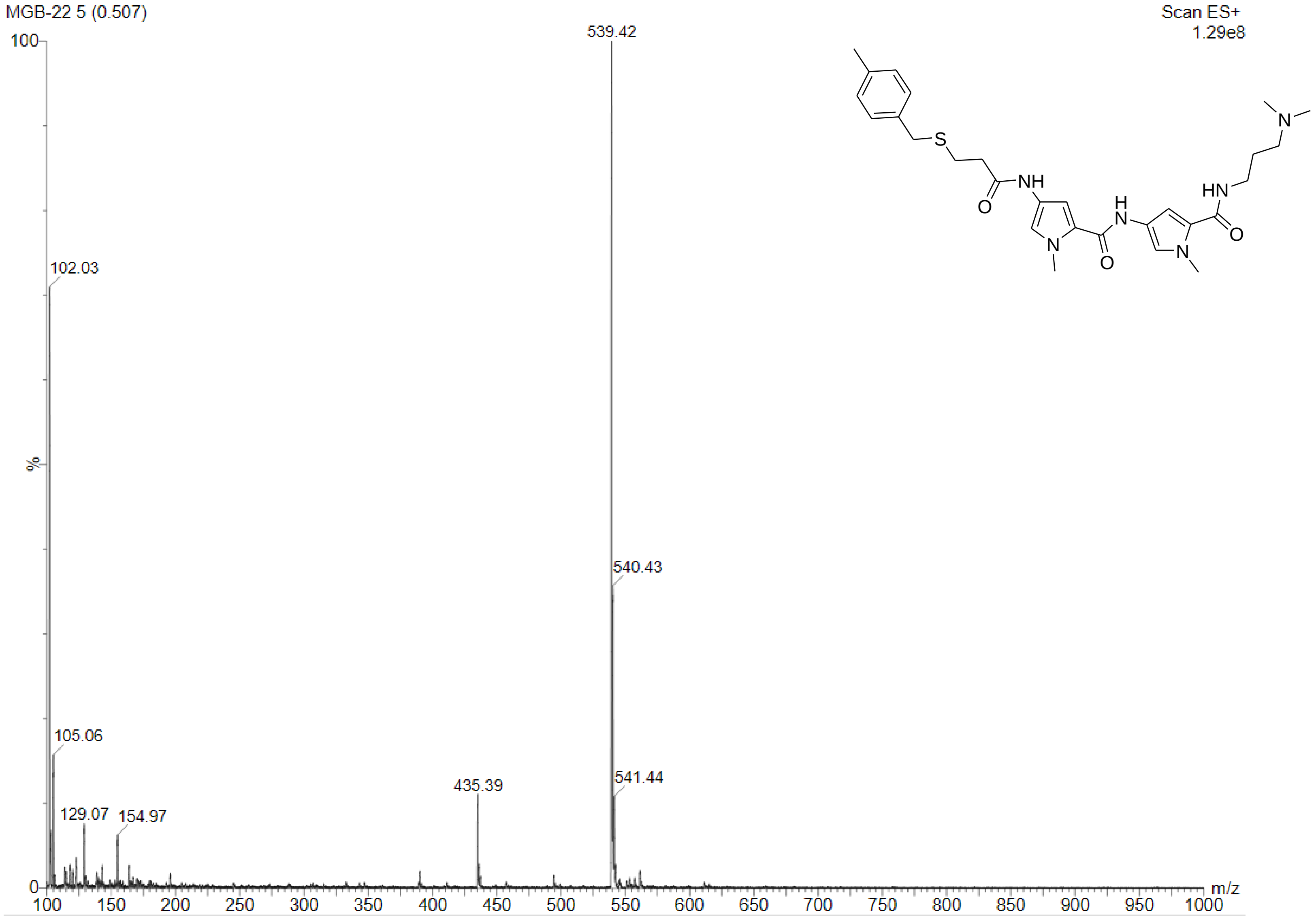

^{1}H NMR (ACETIC ACID-d$_4$) δ, 2.14 (2H, m, CH_2), 2.28 (3H, s, CH_3), 2.54 (2H, t, SCH_2), 2.71 (2H, t, NHCO--CH_2), 3.22 (2H, t, NCH_2), 3.36 (2H, t, NCH_2), 3.49 (2H, q, CONH--CH_2), 3.67 (2H, d, NCH_2), 3.72 (2H, s, SCH_2—Ar), 3.89 (3H, s, NCH_3), 3.90 (3H, s, NCH_3), 3.94 (2H, d, OCH_2), 4.08 (2H, d, OCH_2), 6.80 (1H, s, Ar—H), 6.91 (1H, s, Ar—H), 7.10 (1H, s, Ar—H), 7.12 (1H, s, Ar—H), 7.21 (1H, s, Ar—H), 7.23 (1H, s, Ar—H), 7.25 (1H, s, Ar—H), 7.86 (1H, s, CONH), 9.04 (1H, s, CONH), 9.18 (1H, s, CONH) ; **(MGB24)**

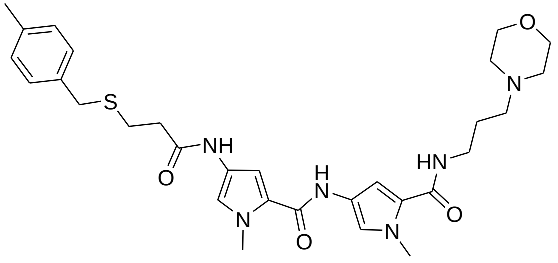

MGB-24-1H.esp

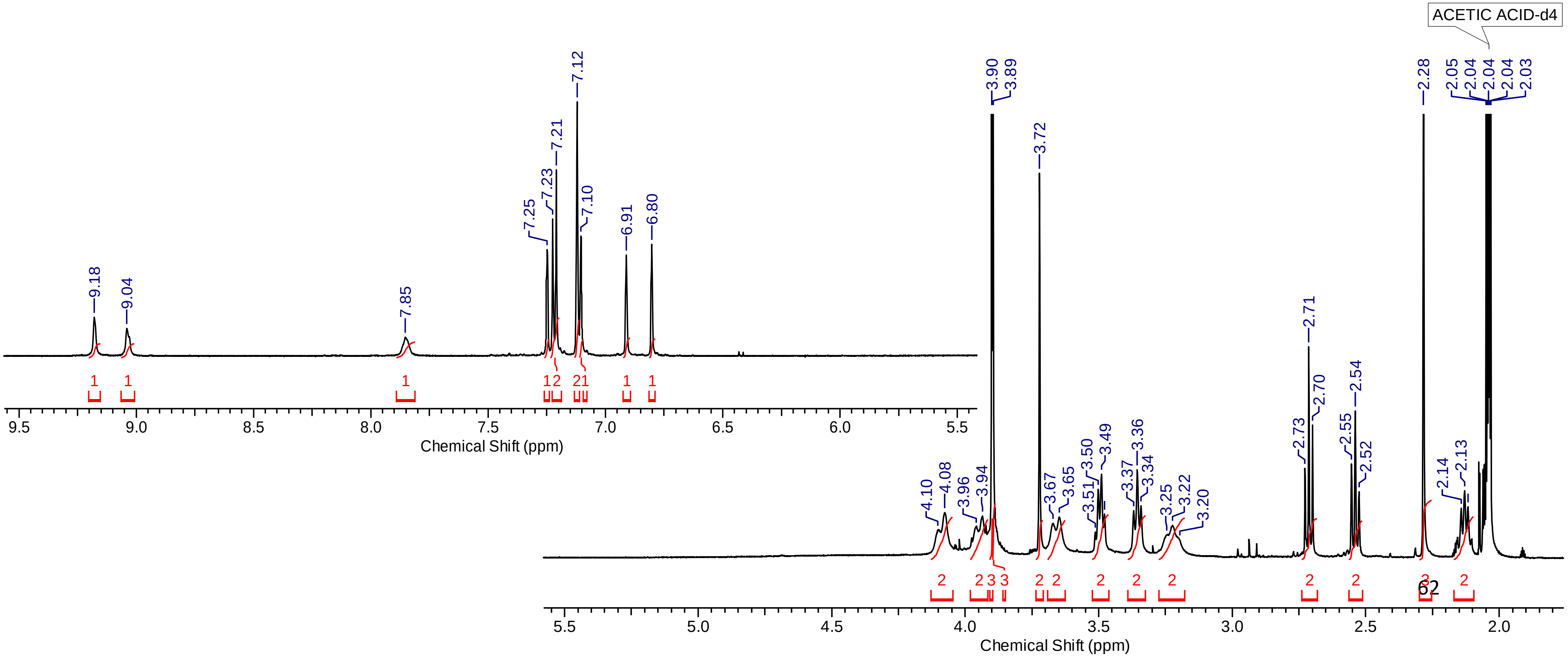

^{13}C NMR (ACETIC ACID-d_4): δ 11.07, 14.85, 17.67, 26.17, 26.60, 27.01, 27.06, 42.48, 45.05, 54.72, 94.30, 95.22, 108.98, 113.12, 113.26, 113.98, 119.72, 119.84, 119.98, 126.61, 127.12, 149.54, 153.87, 158.65; **(MGB24)**

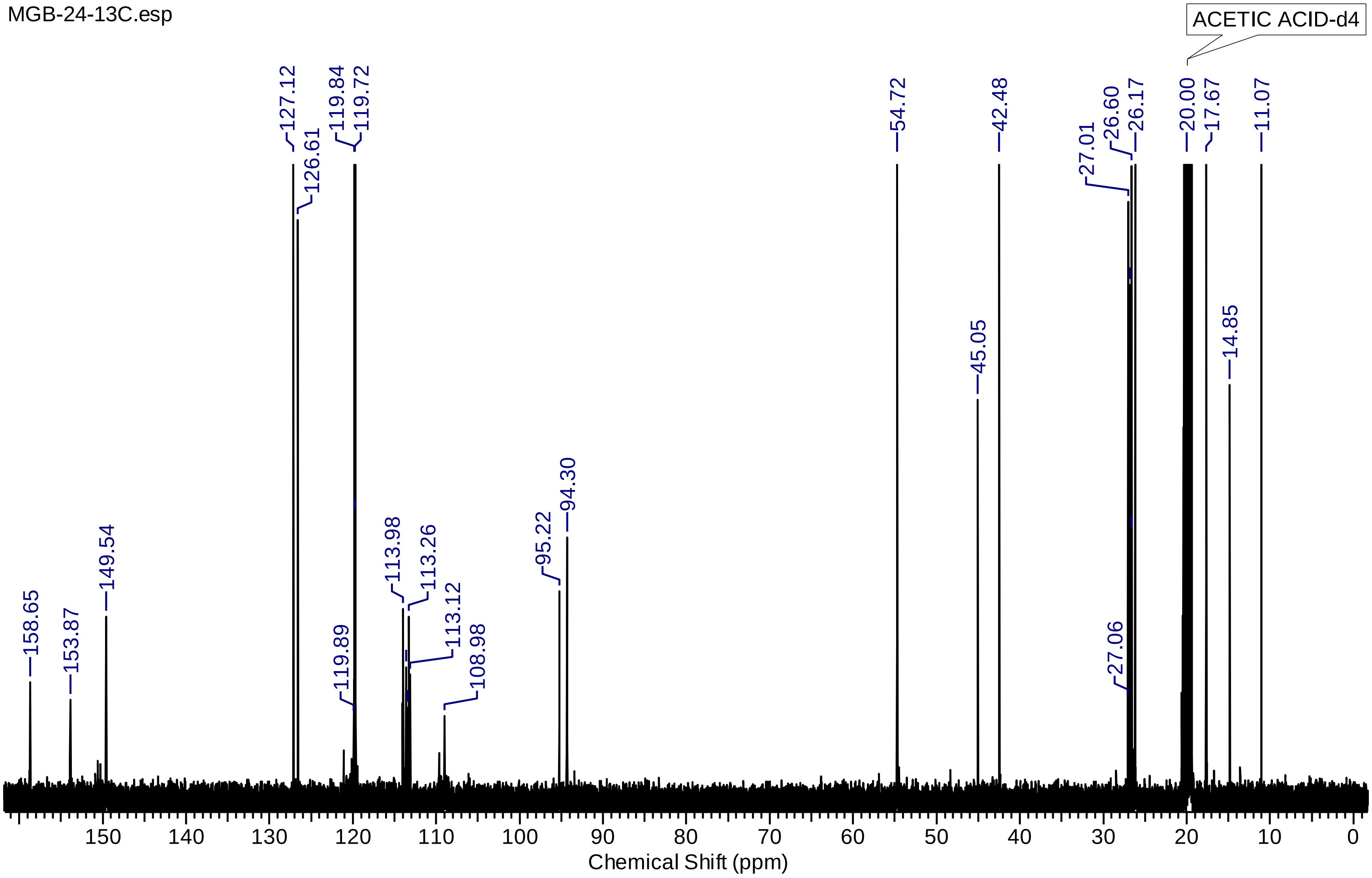

LC−MS (ESI): m/z calcd for $C_{30}H_{40}N_6O_4S$, 580.28, found 581.45 [M + H]+. **(MGB24)**

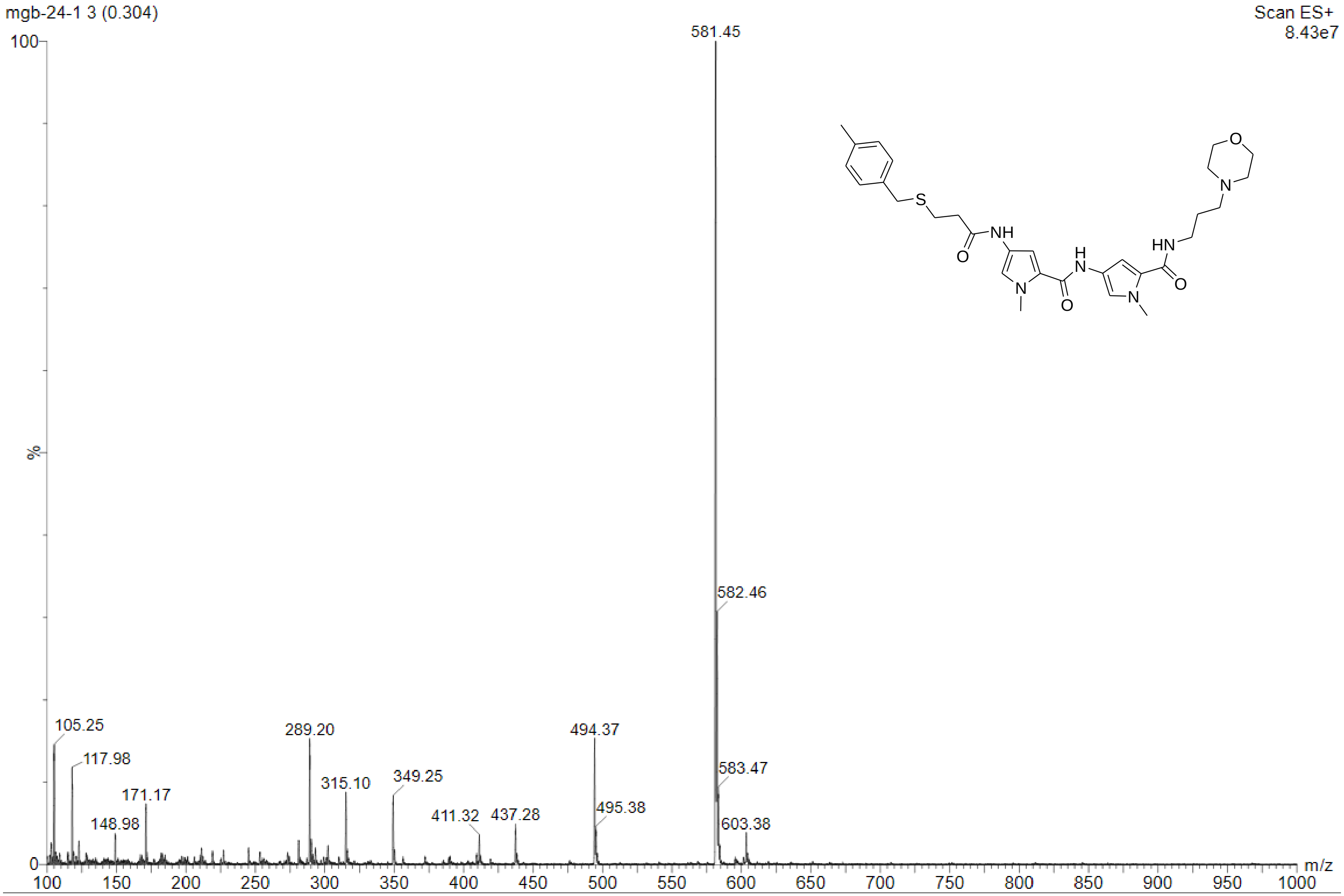

^{1}H NMR (ACETIC ACID-d$_4$) δ 2.94 (6H, s, NCH$_3$), 3.27 (2H, t, NCH$_2$), 3.45 (2H, q, CONH--CH$_2$), 3.89 (3H, s, NCH$_3$), 3.95 (3H, s, NCH$_3$), 6.87 (1H, s, Ar—H), 7.02 (1H, s, Ar—H), 7.27 (1H, d, Ar—H), 7.34 (1H, d, Ar—H), 7.43 (2H, d, Ar—H), 7.44 (1H, d, Ar—H), 7.57 (2H, m, Ar--H), 7.64 (2H, d, Ar—H), 7.98 (2H, d, Ar—H), 9.25 (1H, s, CONH), 9.71 (1H, s, CONH) ; **(MGB26)**

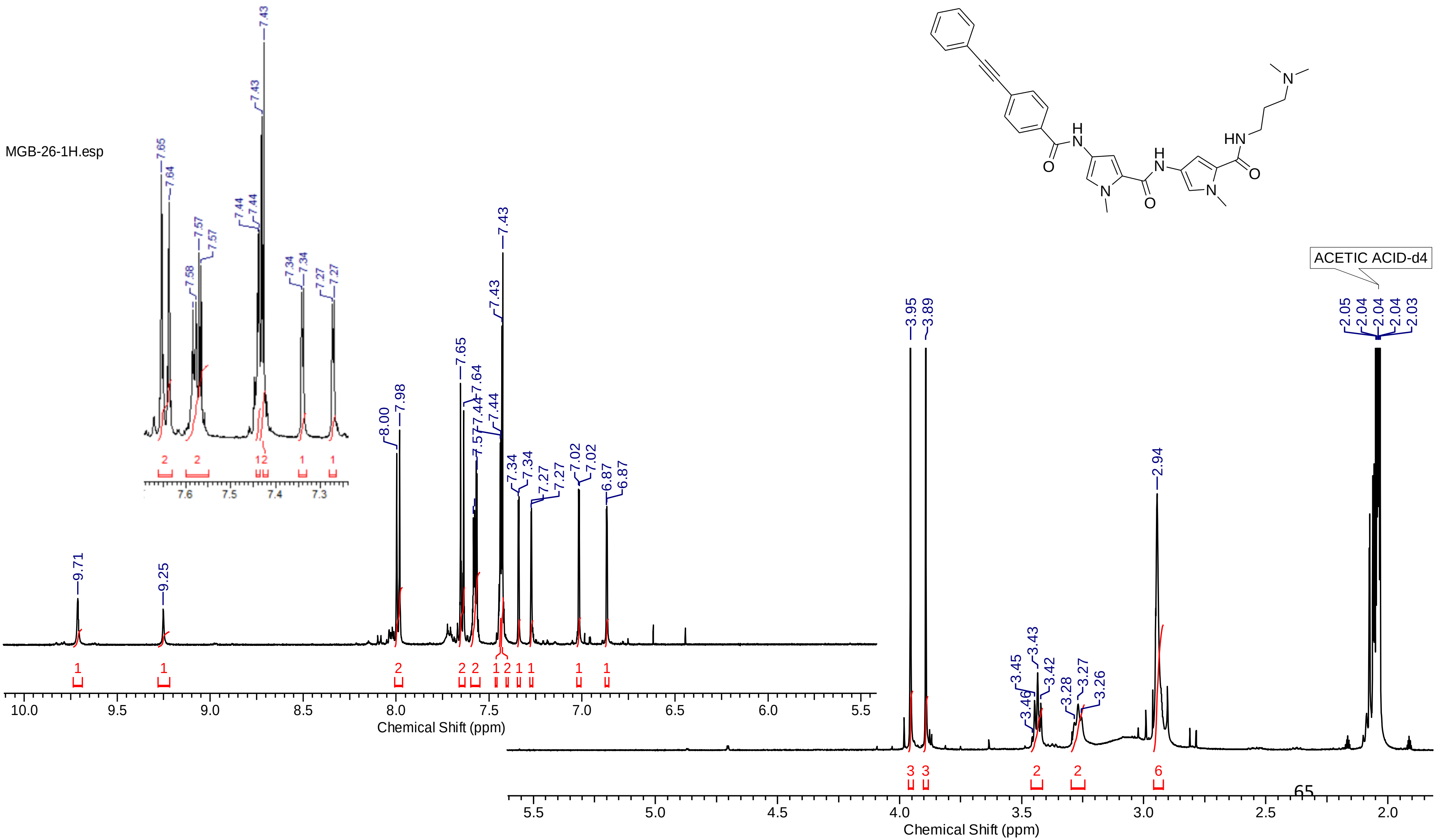

^{13}C NMR (ACETIC ACID-d_4): δ 15.94, 26.92, 27.03, 33.18, 46.04, 79.70, 82.37, 95.06, 95.18, 109.53, 109.94, 113.64, 113.79, 113.95, 117.05, 118.70, 119.86, 122.59, 122.75, 125.87, 149.84, 153.38, 154.10; **(MGB26)**

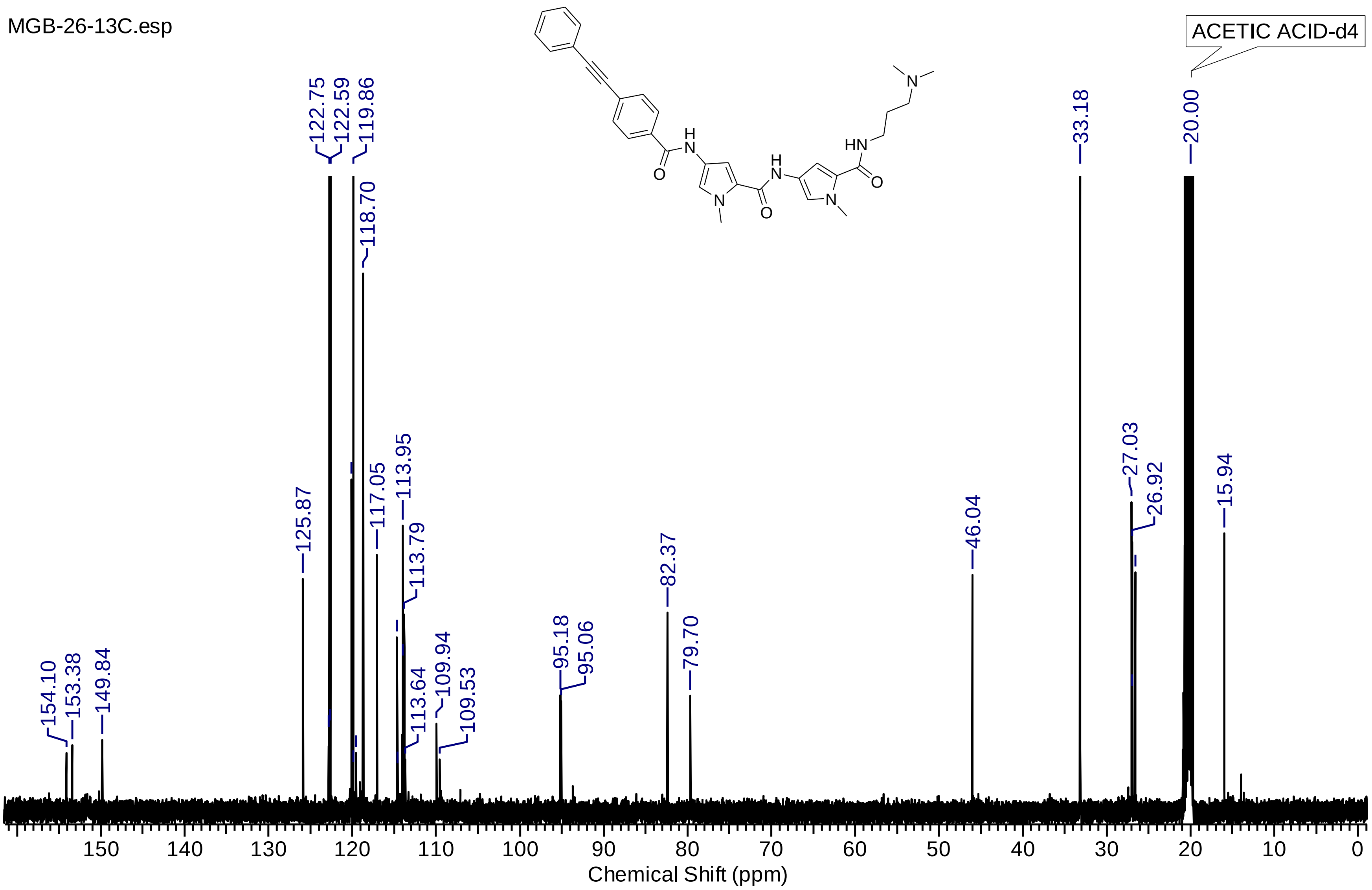

LC−MS (ESI): m/z calcd for $C_{32}H_{34}N_6O_3$, 550.27, found 551.25 [M + H]+. **(MGB26)**

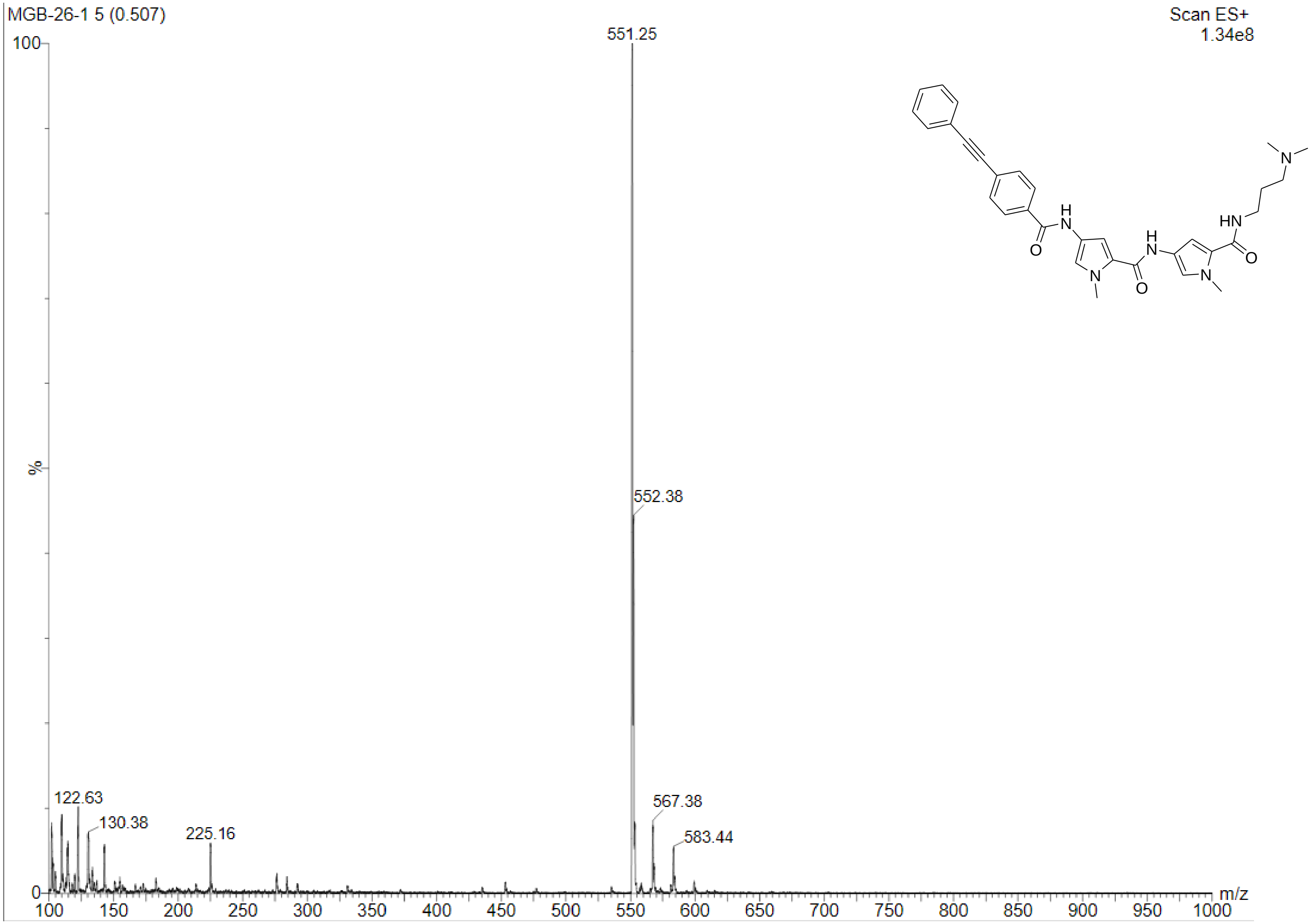

^{1}H NMR (ACETIC ACID-d_4) δ 2.10 (2H, m, CH_2), 3.16 (4H,s, NCH_2), 3.29 (2H, t, NCH_2), 3.46 (2H, q, CONH—CH_2), 3.58 (2H, s, OCH_2), 3.89 (3H, s, NCH_3), 3.95 (3H, s, NCH_3), 4.00 (2H, s, OCH_2), 6.88 (1H, s, Ar—H), 7.01 (1H, s, Ar—H), 7.27 (1H, d, Ar—H), 7.33 (1H, s, Ar—H), 7.43 (2H, d, Ar—H), 7.44 (1H, d, Ar—H), 7.57 (2H, m, Ar--H), 7.64 (2H, d, Ar—H), 7.98 (2H, d, Ar—H), 9.25 (1H, s, CONH), 9.70 (1H, d, CONH) ; **(MGB28)**

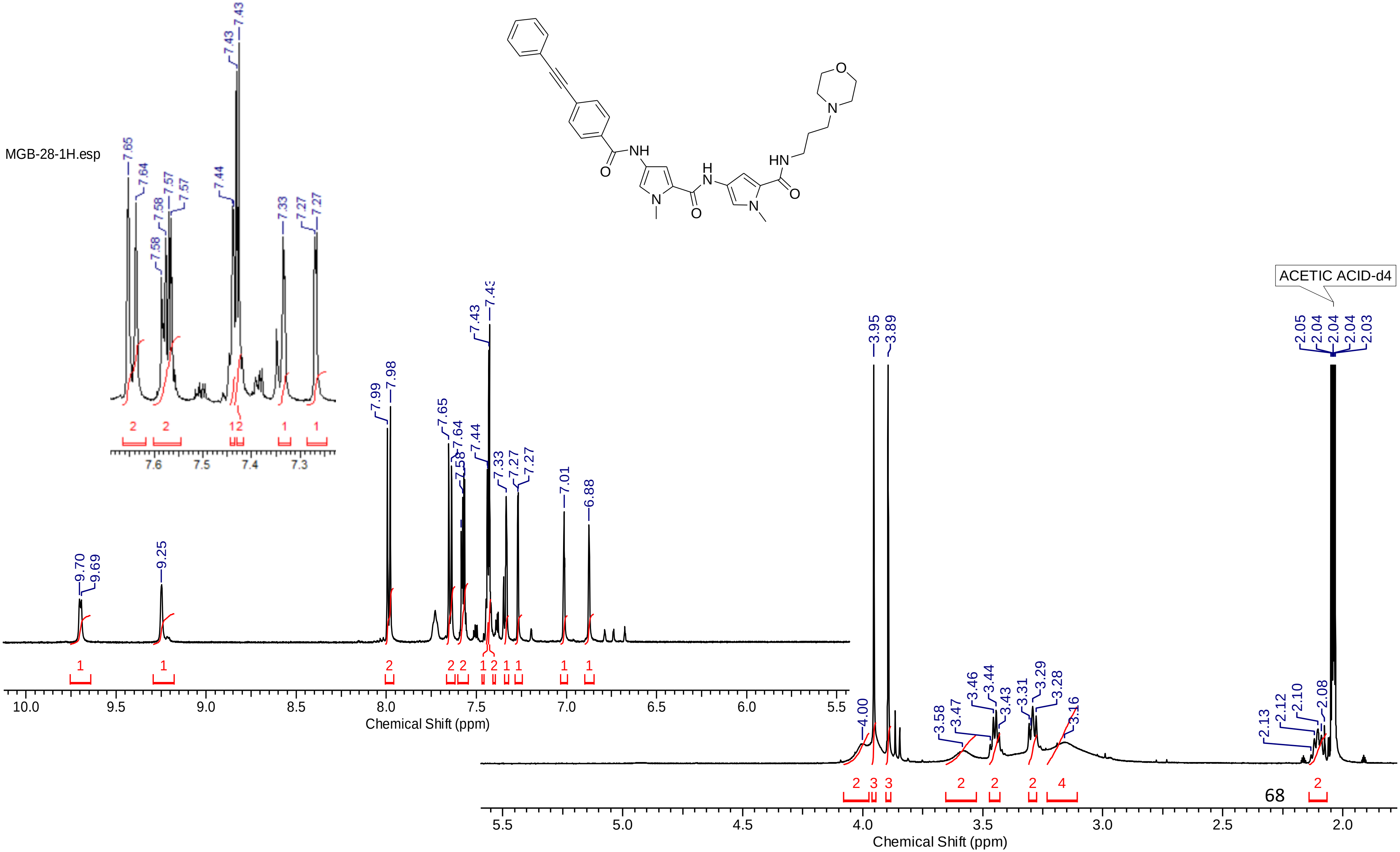

^{13}C NMR (ACETIC ACID-d_4): δ10.99, 14.69, 26.49, 26.65, 26.69, 27.01, 42.41, 45.38, 54.55, 79.38, 82.07, 94.80, 94.85, 103.79, 109.29, 109.61, 113.32, 113.43, 113.63, 116.75, 118.38, 119.56, 122.29, 122.44, 125.55, 128.94, 149.53, 153.14, 153.79; **(MGB28)**

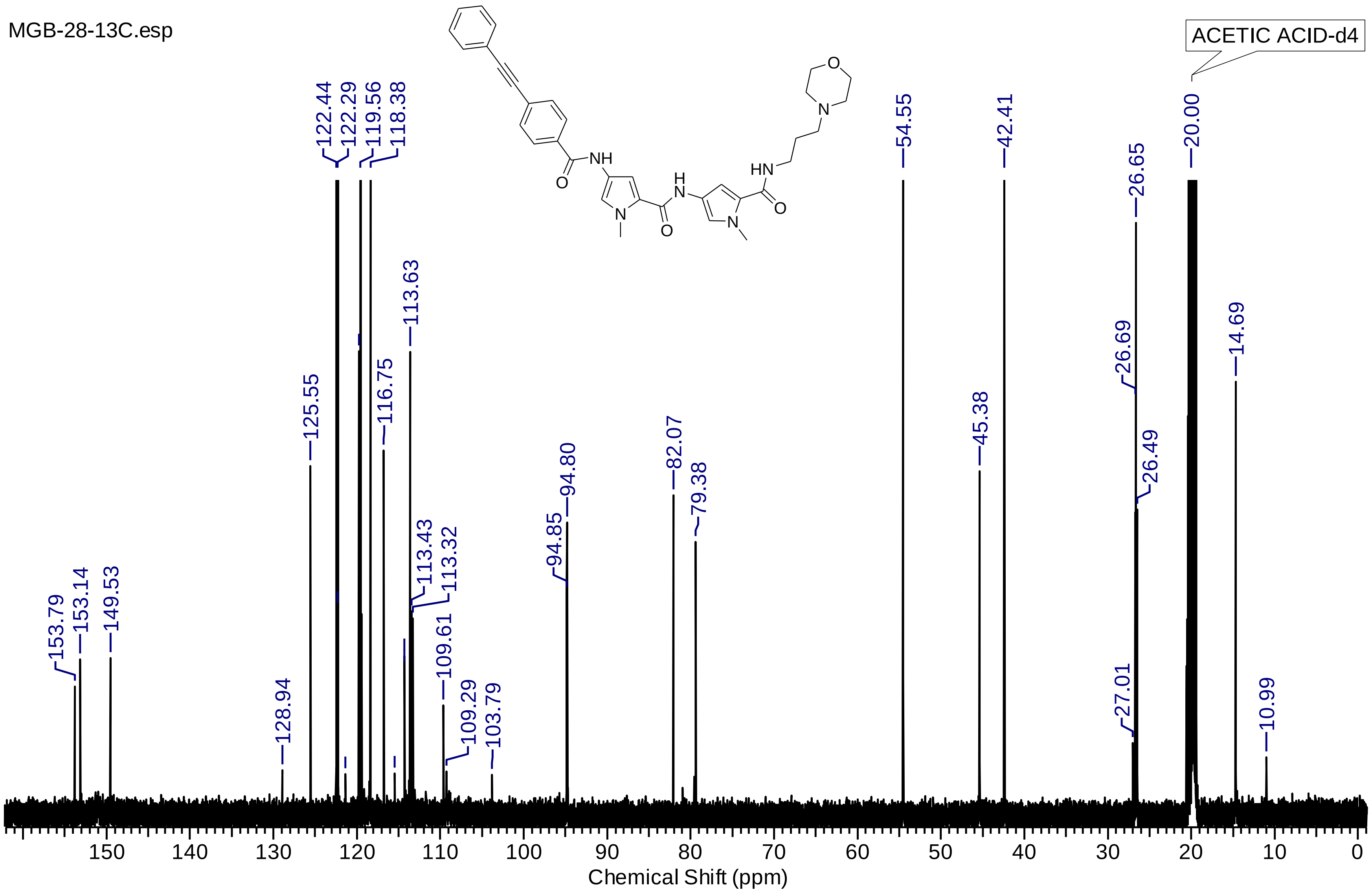

LC−MS (ESI): m/z calcd for $C_{34}H_{36}N_6O_4$, 592.28, found 593.49 [M + H]+. **(MGB28)**

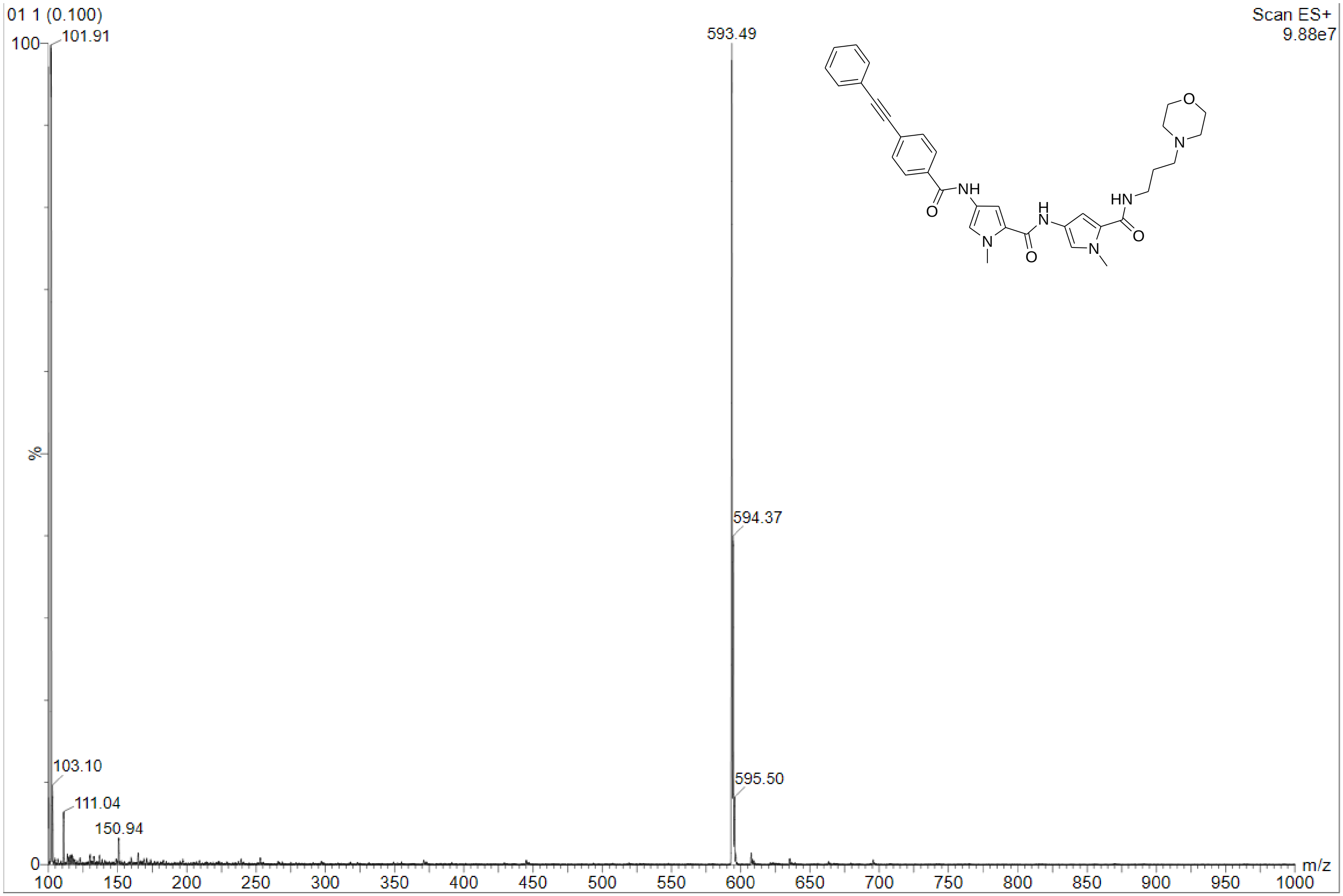

^{1}H NMR (ACETIC ACID-d_4) δ 2.08 (2H, m, CH_2), 2.95 (6H, s, NCH_3), 3.26 (2H, t, NCH_2), 3.44 (2H, q, CONH--CH_2), 3.89 (3H, s, NCH_3), 3.95 (3H, s, NCH_3), 6.88 (1H, s, Ar—H), 7.02 (1H, s, Ar—H), 7.28 (2H, d, Ar—H), 7.30 (1H, d, Ar—H), 7.35 (2H, m, Ar—H), 7.37 (1H, s, HC=CH), 7.38 (1H, d, HC=CH), 7.63 (2H, d, Ar—H), 7.71 (2H, d, Ar—H), 7.97 (2H, d, Ar—H), 9.27 (1H, d, CONH), 9.67 (1H, d, CONH) ; **(MGB30)**

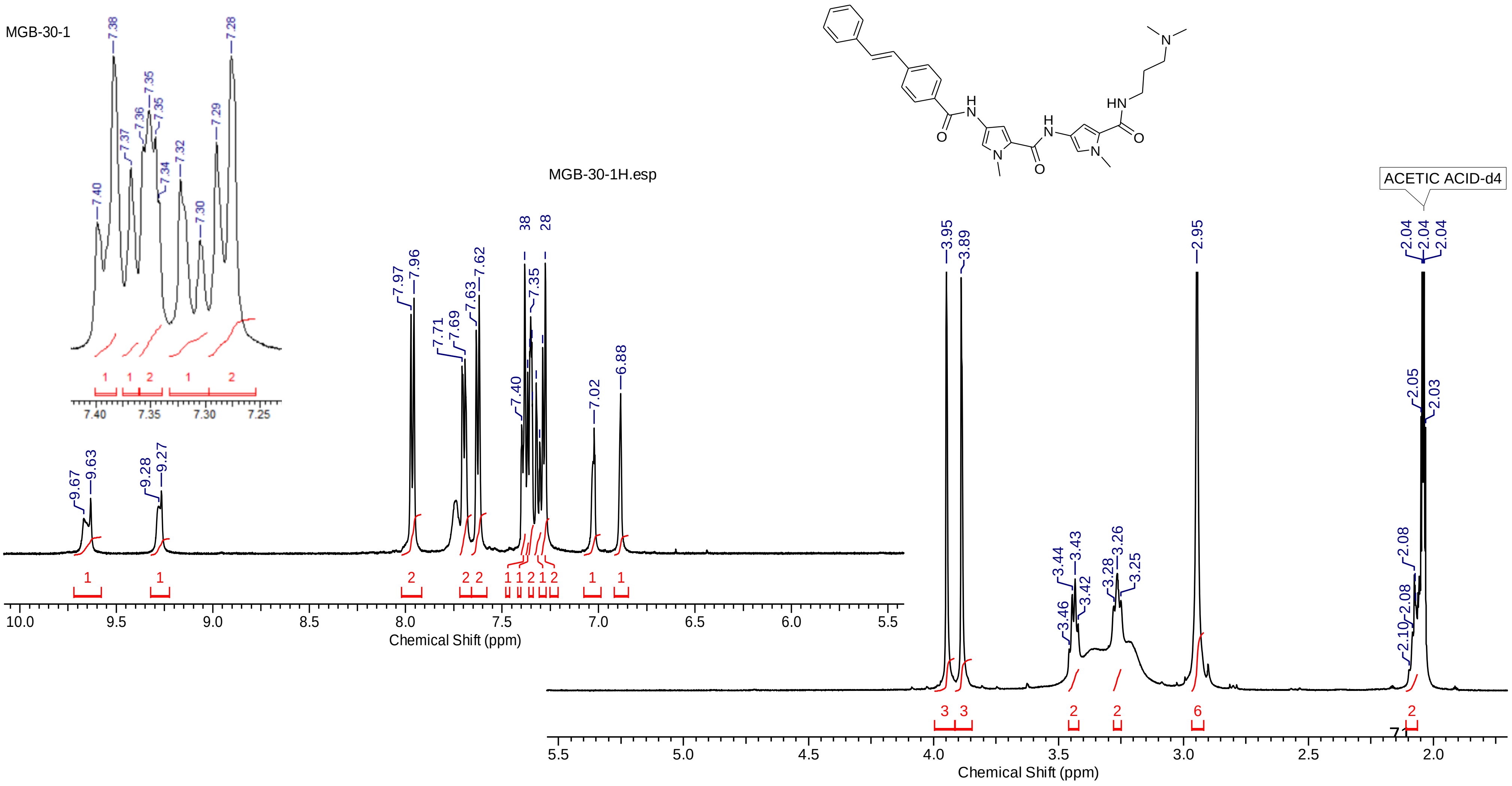

^{13}C NMR (ACETIC ACID-d_4): δ 15.65, 26.28, 26.62, 26.67, 32.89, 45.74, 94.79, 94.89, 113.46, 113.48, 114.20, 117.26, 117.61, 119.62, 121.29, 124.67, 128.06, 131.21, 149.57, 153.13, 154.22; **(MGB30)**

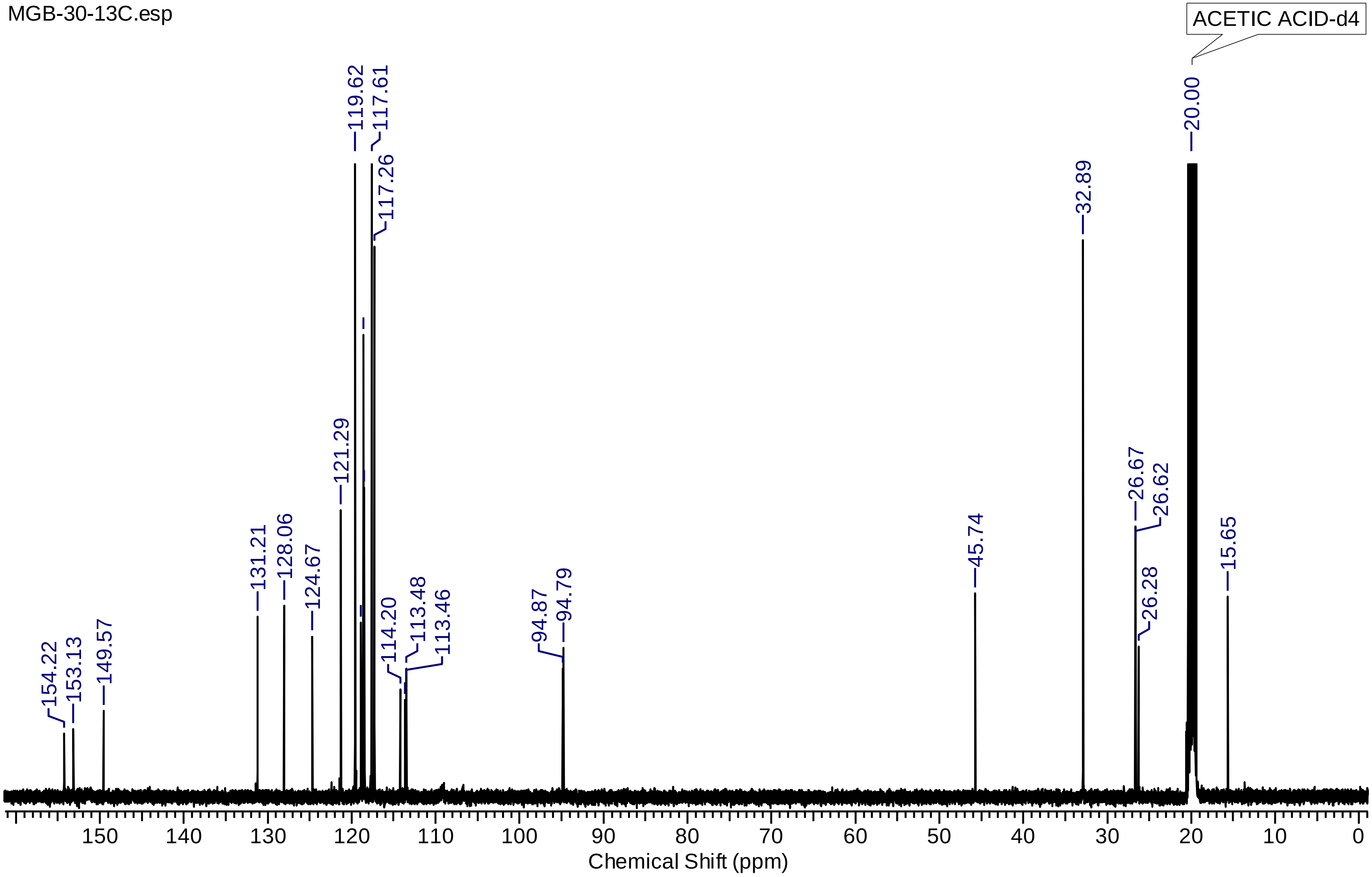

LC−MS (ESI): m/z calcd for $C_{32}H_{36}N_6O_3$, 552.28, found 553.29 [M + H]+. **(MGB30)**

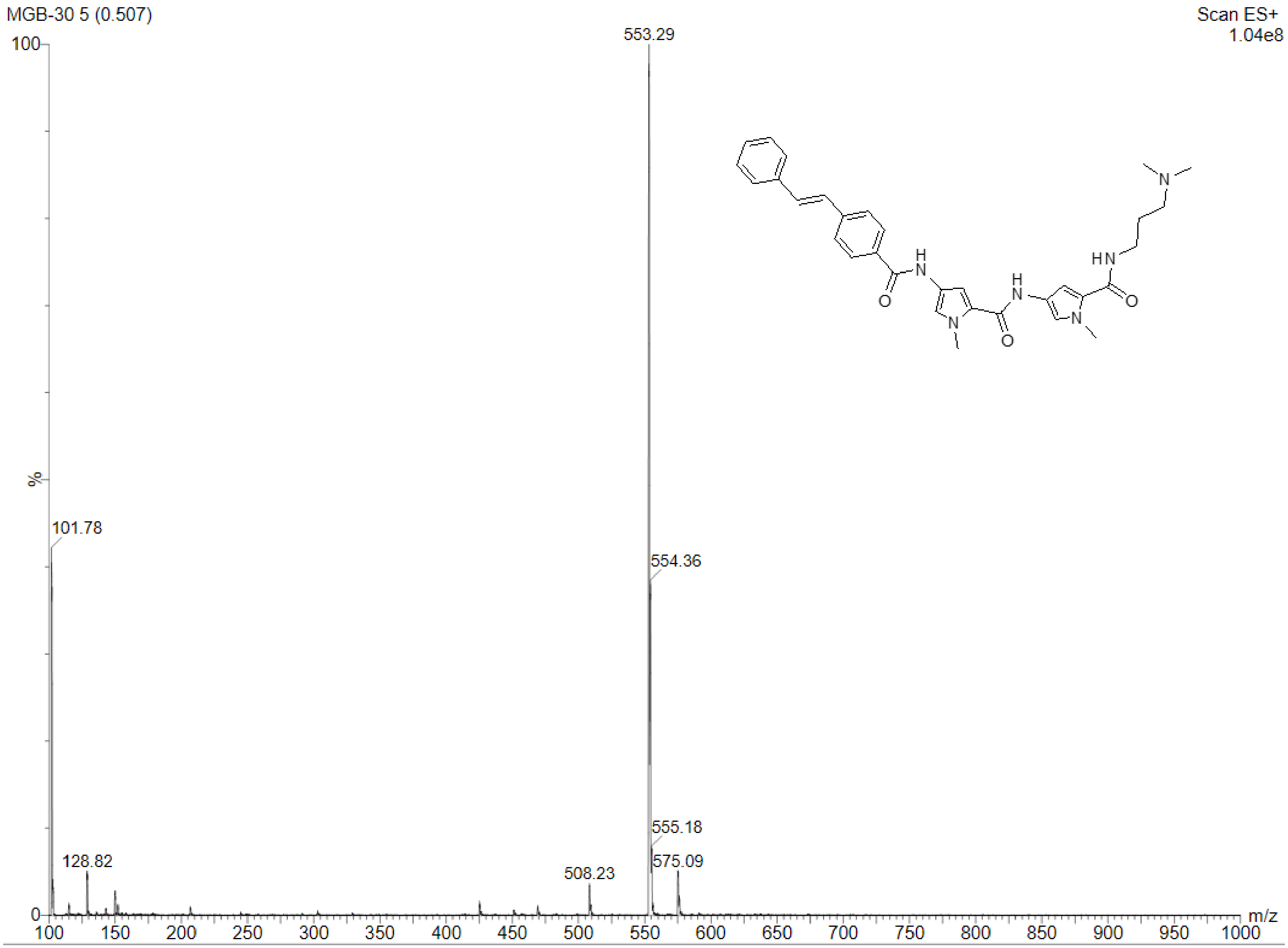

^{1}H NMR (ACETIC ACID-d_4) δ 2.11 (2H, m, CH_2), 3.10 (4H, s, NCH_2), 3.30 (2H, t, NCH_2), 3.46 (2H, q, CONH—CH_2), 3.59 (2H, s, OCH_2), 3.89 (3H, s, NCH_3), 3.95 (3H, s, NCH_3), 4.01 (2H, s, OCH_2), 6.88 (1H, s, Ar—H), 7.01 (1H, s, Ar—H), 7.27 (1H, s, Ar—H), 7.31 (1H, d, Ar—H), 7.33 (2H, s, Ar—H), 7.36 (1H, d, Ar—H), 7.39 (1H, s, HC=CH), 7.40 (1H, d, HC=CH), 7.62 (2H, d, Ar--H), 7.71 (2H, d, Ar—H), 7.97 (2H, d, Ar—H), 9.25 (1H, s, CONH), 9.60 (1H, s, CONH) ; **(MGB32)**

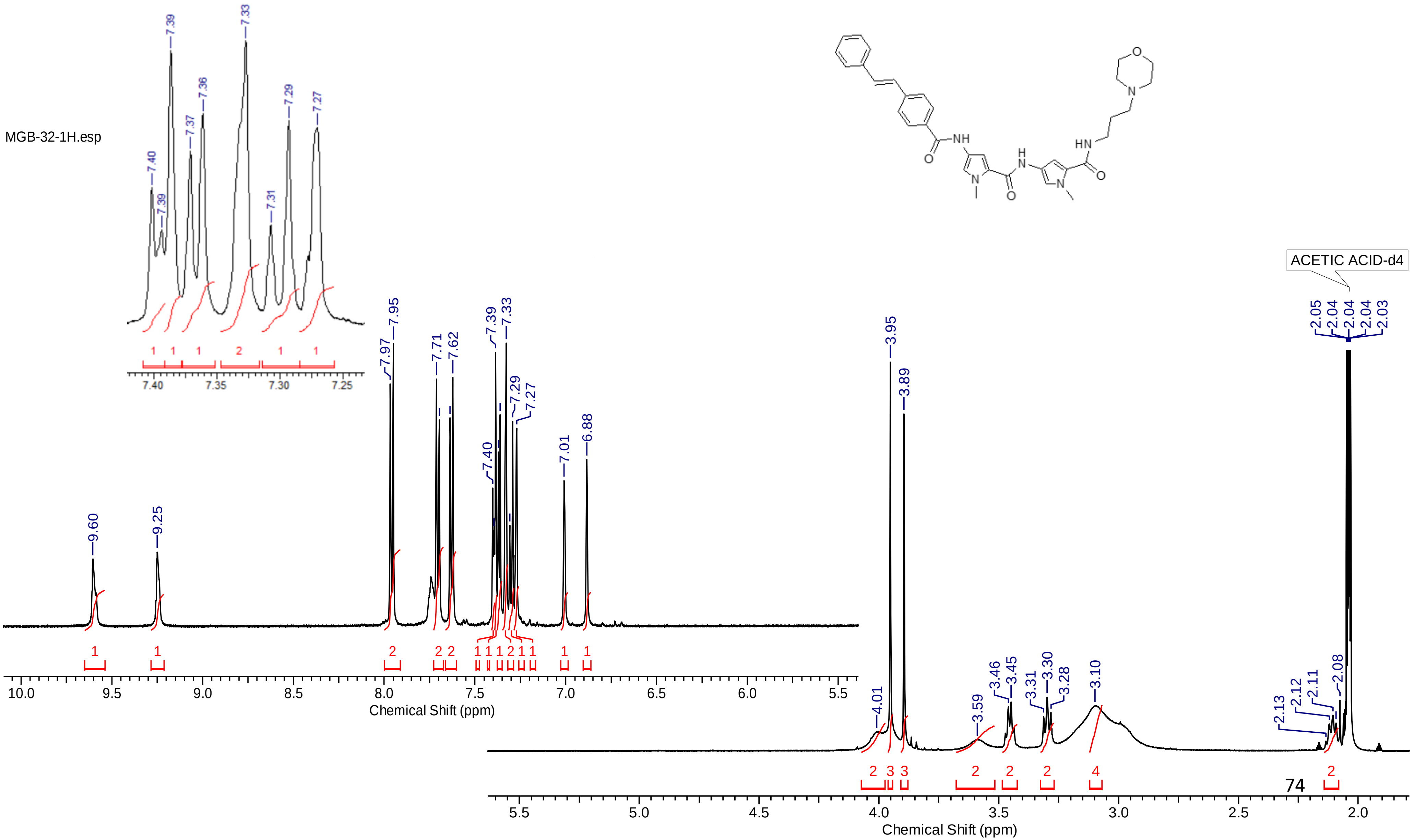

^{13}C NMR (ACETIC ACID-d_4): δ 14.84, 26.64, 26.81, 26.83, 42.57, 45.54, 54.70, 94.96, 95.00, 113.62, 113.63, 114.36, 117.42, 117.77, 119.78, 121.46, 124.83, 128.21, 131.38, 149.72, 153.31, 154.36; **(MGB32)**

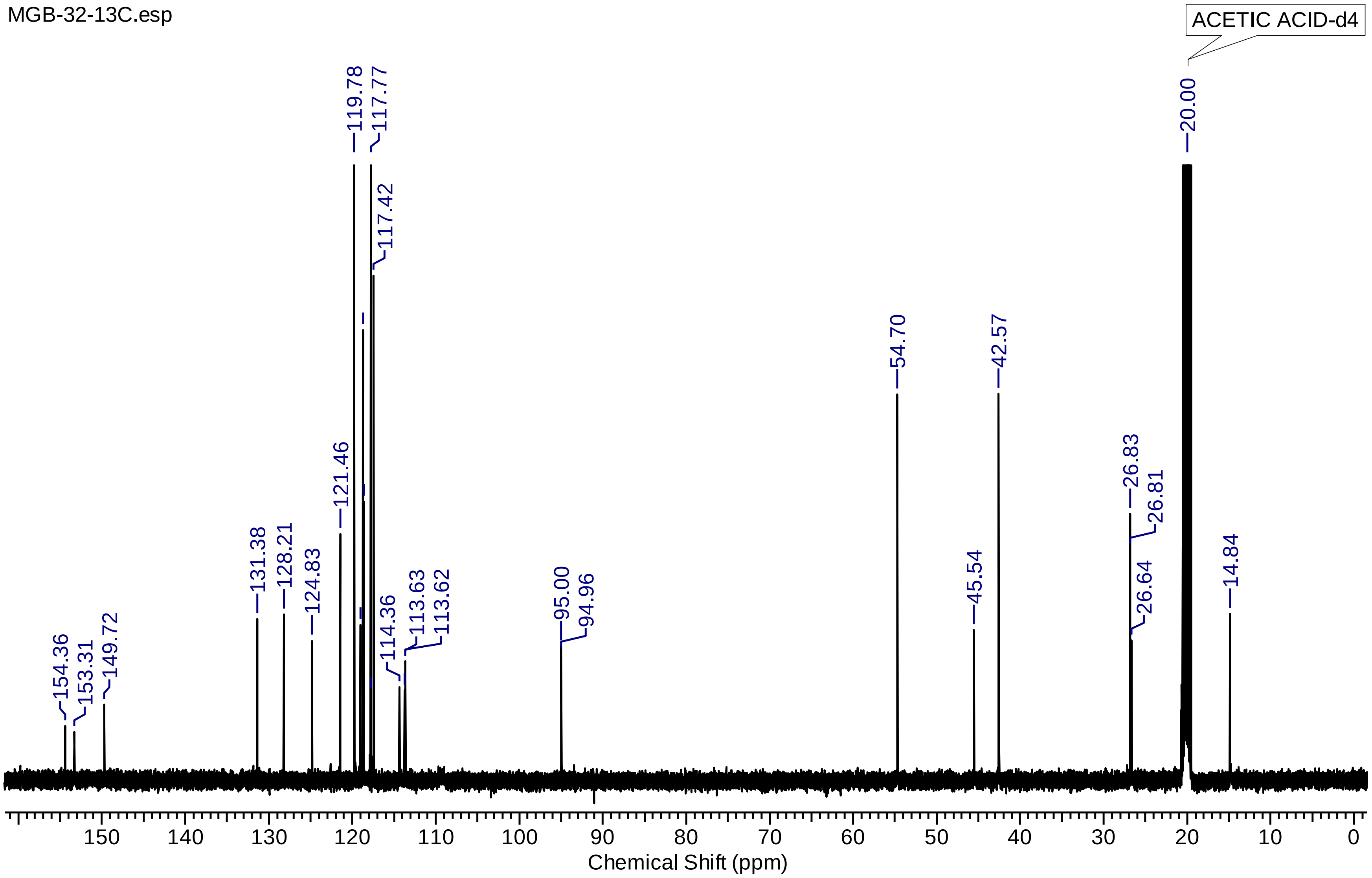

LC−MS (ESI): m/z calcd for $C_{34}H_{38}N_6O_4$, 594.3, found 595.44 [M + H]+. **(MGB32)**

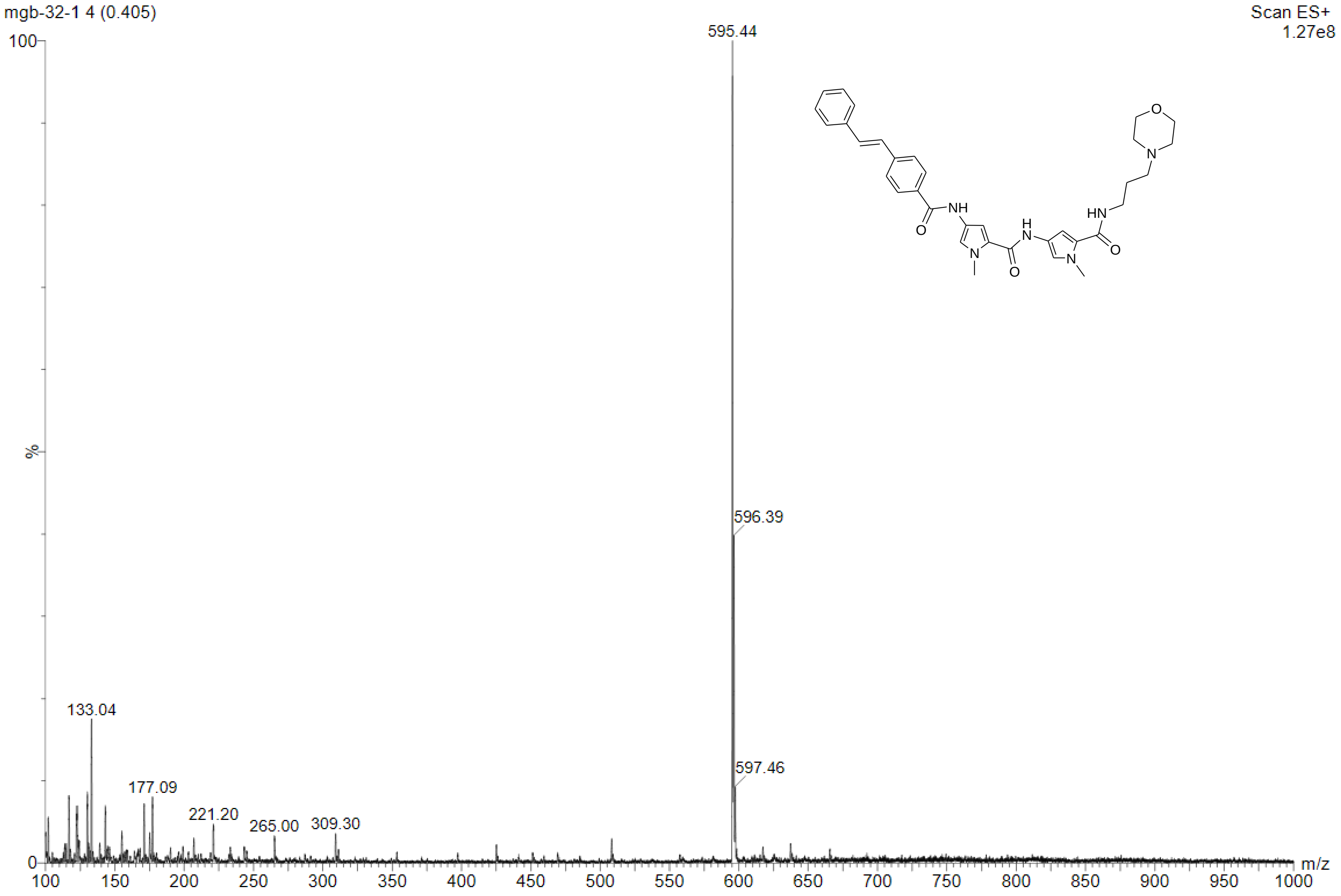

Supplement: Supplementary file 1 [file antibiotics-11-00935-s001.zip › antibiotics-1771743-supplementary.pdf]
